# Global analysis of protein degradation reveals instability of diverse regulators in *Escherichia coli*

**DOI:** 10.1073/pnas.2515265123

**Published:** 2026-03-03

**Authors:** Elliot J. MacKrell, Brett Lomenick, Yanping Qiu, Hannah Jeckel, Jeff Jones, Tsui-Fen Chou, David A. Tirrell

**Affiliations:** ^a^Division of Chemistry and Chemical Engineering, California Institute of Technology, Pasadena, CA 91125; ^b^Proteome Exploration Laboratory, Beckman Institute, California Institute of Technology, Pasadena, CA 91125; ^c^Division of Biology and Biological Engineering, California Institute of Technology, Pasadena, CA 91125; ^d^Division of Physics, Mathematics and Astronomy, California Institute of Technology, Pasadena, CA 91125

**Keywords:** *Escherichia coli*, protein degradation, proteomics, BONCAT, machine learning

## Abstract

Regulated protein degradation underlies the timely execution of essential gene expression programs in bacteria. Here, we deployed time-resolved chemoproteomics, text mining of the PubMed and EcoCyc knowledge bases, and machine learning classification to identify proteolytic regulation in exponential and stationary phase *Escherichia coli* cultures. We experimentally validated the instability of diverse homeostatic and stress response regulators, including the principal cyclic-di-GMP phosphodiesterase PdeH, the N-end rule substrate chaperone ClpS, and all four A-type domain iron–sulfur cluster carriers, IscA, ErpA, NfuA, and SufA. Mutagenesis of the PdeH N-terminal extension abolished ClpXP recognition, thereby impairing stationary phase depletion of PdeH and altering macrocolony biofilm surface morphology. Unstable proteins synthesized in stationary phase such as the morphology regulator BolA, RNA polymerase ω subunit, and the biofilm regulator BssR were implicated in quiescence. Finally, machine learning–assisted substrate identification revealed Lon-mediated degradation of two opposing key regulators of surface adhesion, the RpoS antagonist FliZ and the major biofilm regulator CsgD, suggesting proteolysis may hasten transitions between motility and sessility. Together, these results highlight the role of regulated proteolysis in driving physiological adaptation for this model organism.

## Significance

The identification of proteins regulated by proteolysis is important for defining homeostatic, developmental, and stress response pathways in bacteria. We present an experimental and computational analysis of protein degradation that reveals protease substrates among nascent proteins in exponential and stationary phase *Escherichia coli* cells. We validate the active degradation of proteins involved in motility, biofilm development, metabolism, and proteolysis itself. Our work highlights the role of protein degradation in shaping protein abundance dynamics across protein functionalities and physiological states and identifies opportunities for further investigation of this essential aspect of proteostasis.

Protein degradation is essential for ameliorating proteotoxicity and achieving full spatiotemporal precision in posttranslational regulation. In bacteria, ATP-dependent protein degradation proceeds through a sequential process of substrate recognition, unfolding, translocation, and subsequent peptide bond cleavage by an AAA+ (ATPases associated with diverse cellular activities) protease (1). Substrate recognition requires the presence of one or more amino acid sequences or structural motifs, or degrons, in the substrate and may further demand the activity of adaptor proteins, which chaperone substrates to their cognate protease(s) under permissive conditions, or endoproteolytic cleavage, which may expose cryptic internal degrons (2–4). *Escherichia coli* bears one essential membrane-bound AAA+ protease, FtsH, and four nonessential cytosolic AAA+ proteases: Lon, ClpXP, ClpAP, and HslUV. Seminal radiolabeling studies demonstrated that a small proportion (ca. 5%) of nascent protein is rapidly degraded under conditions of exponential growth, revealing the rarity of proteolysis in this organism as reflected in its high expression of chaperones dedicated to renaturation rather than elimination of potentially harmful misfolded proteins (5–7). Yet protein degradation is a demonstrated necessity in a broad range of homeostatic, adaptational, and developmental programs, thus establishing a need for identifying protease substrates in faithfully mapping regulatory networks (8–10).

Several strategies exist for uncovering proteolytic regulation. Overexpression of catalytically inactivated AAA+ proteases for affinity purification and identification of trapped substrates has proven successful in several species of bacteria (11, 12). Indeed, a seminal ClpXP trapping analysis under SOS response induction revealed DNA damage–associated substrate enrichment, suggesting the active pool of protease substrates can vary with cell phenotype (13). However, artificially elevated protease abundances may invoke toxicity or perturb endogenous protein abundances or substrate–protease interactions, and such experiments inherently limit the scope of substrate identification and kinetic analysis (14). Alternatively, two recent studies have globally profiled protein degradation kinetics in *E. coli*, though researchers primarily investigated the protein attributes and physiological conditions governing proteolysis rather than validating and exploring the regulatory implications of select substrates’ instabilities (15, 16). Additionally, while the ubiquity and clinical relevance of growth arrest have garnered significant attention, the study of the nascent proteome in quiescent bacterial cells is complicated by low metabolic activity, which biases protein identification toward the preexisting proteome and thereby limits conventional isotopic labeling strategies (17). More broadly, though developments in acquisition and fractionation strategies have considerably advanced quantitative accuracy and proteome coverage, bottom–up mass spectrometry–based proteomics remains biased toward abundant species with permissive physicochemical attributes, which precludes full proteome coverage (18).

**Fig. 1.**
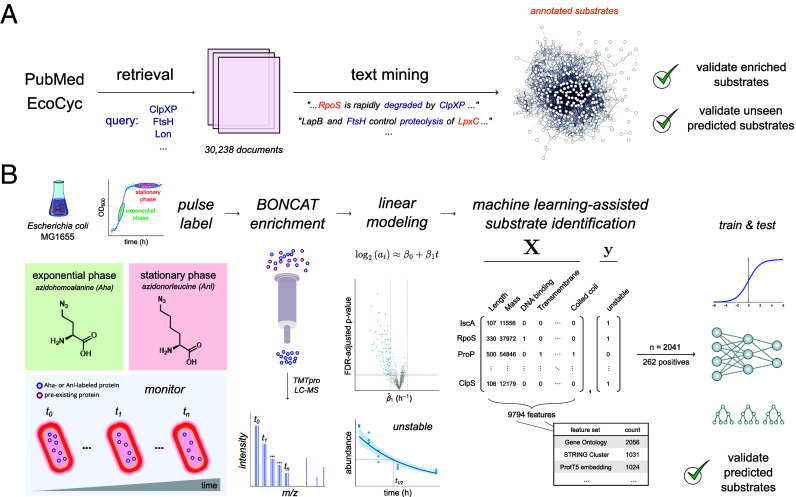
Graphical outline of the experimental procedures in this work. (*A*) Outline of the text mining procedures used in this work. Publication abstracts from PubMed and gene summaries from EcoCyc are queried for terms related to protein degradation in *Escherichia coli*. The publications and summaries returned by this query are searched for sentences that report the proteolytic instability of proteins in the *E. coli* proteome. The proteins yielded by this strategy are examined for functional enrichment and used as a confirmation set for our chemoproteomic analysis and machine learning approach. (*B*) Outline of the substrate identification procedures used in this work. Cells are metabolically labeled in exponential phase or stationary phase with Aha or Anl, respectively. Labeled proteins are then subjected to a chemoproteomic enrichment strategy and analyzed via LC–MS. The mass spectra are then searched against the *E. coli* MG1655 reference proteome to identify proteins that exhibit time-series decay, and their abundance traces are fit to an exponential decay model. The estimated rate coefficients are used to classify proteins as stable or unstable, and these labels are then used to train a machine learning model for predicting in vivo protein stability after sourcing predictive features from public knowledge bases. The trained model is then used to predict protein stability, with predicted candidates examined for instability via immunoblotting stability analysis.

To address these challenges, we validated and expanded the application of bioorthogonal noncanonical amino acid tagging (BONCAT), a time-resolved chemical labeling method, in the analysis of bacterial protein stability (Fig. 1). The BONCAT method operates through the treatment of cells with an azide- or alkyne-bearing amino acid surrogate for azide-alkyne cycloaddition–based “click” conjugation to enrichment resins, thereby enabling time-resolved chemoproteomic identification of labeled proteins (19). Owing to its sensitivity in labeling nascent proteomes, BONCAT has demonstrated potential in dissecting microbial dormancy through the analysis of protein synthesis in quiescent cells in vitro, in vivo, and in situ (20–22). Further, a BONCAT analysis in mammalian cells revealed substantial heterogeneity in the degradation kinetics of nascent proteins, demonstrating the utility of this method in proteome-wide degradation profiling (23). While the most widespread adoption of the BONCAT method involves the treatment of cells with azidohomoalanine (Aha), the mutant methionyl-tRNA synthetase NLL-MetRS efficiently charges tRNA^Met^ with azidonorleucine (Anl), providing elevated methionine replacement rates and enabling state- or cell-selective analysis (24–26). Here, we leveraged the temporal resolution and sensitivity of these labeling strategies in combination with multiplexed isobaric tandem mass tag (TMT) labeling to profile the stability of nascent proteins in both exponential and quiescent stationary phase cells. To support our identifications, we confirmed the enrichment of established substrates among proteins identified in our screens by comparing their identities with those sourced by text mining public databases. We identified instability in a diverse panel of homeostatic and stress response regulators and mapped select substrates to their cognate proteases. We then used our identifications to train and validate a machine learning classifier for predicting in vivo protein stability, identifying the active degradation of key regulators that drive motility and surface adhesion. Taken together, our observations expand the catalog of established ATP-dependent degradation across diverse domains of microbial physiology.

## Results

### Text Mining Establishes a Reference Set of Actively Degraded Proteins.

While the study of regulated proteolysis in bacteria is an established field, substrate identifications are not thoroughly reflected in existing knowledge bases. To consolidate knowledge on this topic, we first reviewed the literature to manually curate the identities of proteins bearing direct or inferential evidence of regulated proteolysis, which we refer to hereafter as annotated substrates. We then constructed and queried an integrated corpus of 30,238 documents containing PubMed abstracts or EcoCyc gene summaries to augment our manual curation (27). Sentences were searched for *E. coli* gene names and terms describing protein degradation to yield candidate annotations that were then approved or rejected in a specialized Shiny application upon manual inspection of the identified terms and their corresponding publications (*SI Appendix,* Fig. S1*A* and Dataset S1) (28). Text mining expanded our set of manually curated substrate annotations by 38%, with each knowledge base yielding unique identifications (*SI Appendix,* Fig. S1*B*). Frequently identified genes in this query were classically associated with protein degradation (*SI Appendix*, Fig. S2*A*). Concatenation of text-mined and manually curated annotations yielded a dense network of 364 proteins enriched in computational and experimental interactions, suggesting these proteins are functionally associated (*SI Appendix*, Fig. S2*B*). Enriched annotations from the UniProt and Kyoto Encyclopedia of Genes and Genomes (KEGG) knowledge bases included functions that are characteristic of regulated proteolysis in bacteria such as DNA repair, metal homeostasis, and sigma factor activity in addition to others such as RNA degradation and acetylation that are not broadly recognized in reviews of regulated proteolysis in bacteria (*SI Appendix,* Fig. S2*C*) (7, 29–32).

### BONCAT Identifies Proteolytic Degradation in Exponential Phase.

**Fig. 2.**
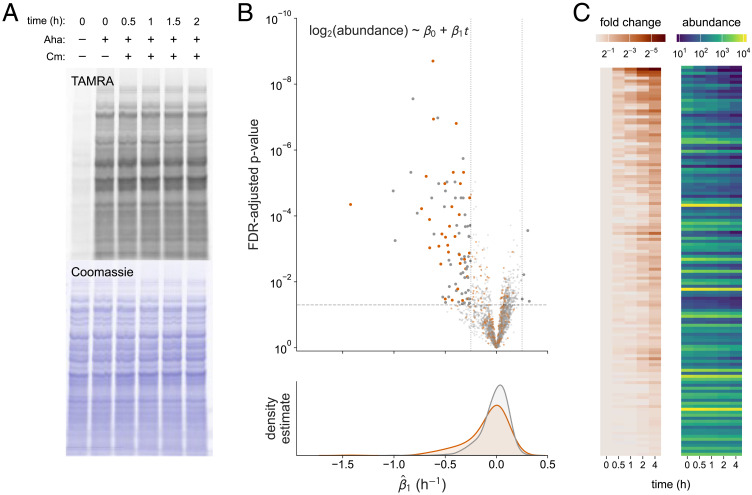
Global stability analysis in exponential phase cells. (*A*) In-gel fluorescence of labeled proteins conjugated to TAMRA-alkyne. Cells were collected at the indicated time points during the observation window after treatment with Aha for 30 min and subsequent treatment with chloramphenicol to inhibit protein synthesis. Coomassie staining reports total loading. (*B*) Statistical significance and kernel density estimates of fitted degradation rates (n = 1,810 proteins) stratified by annotation status. Vertical dotted lines indicate a rate constant that yields a fold change of two over the course of the experiment (estimated half-life < 4 h), and the horizontal line indicates an adjusted *P*-value < 0.05, with 34 annotated substrates and 54 unannotated substrates exceeding these thresholds. Orange shading represents annotated substrates, and gray shading indicates a lack of evidence of instability in the literature. (*C*) Heatmaps reporting mean abundances for proteins with evidence of exponential decay (estimated half-life < 4 h, FDR-adjusted *P*-value < 0.05, R^2^ > 0.5) in the screen with (*Left*) and without (*Right*) normalization to their initial abundance.

We next sought to expand our annotation set by profiling the stability of nascent protein populations with the BONCAT technique. We treated exponential phase *E. coli* MG1655 cells with Aha for 30 min to label nascent proteins in this physiological state. To eliminate the limiting effect of growth on the quantitative dynamic range attributable to proteolysis in subsequent proteome-wide analysis, we employed the bacteriostatic translation inhibitor chloramphenicol as a postlabeling treatment in an analogous manner to cycloheximide treatments in eukaryotic proteome turnover analyses (33, 34). Lysates of cells harvested at selected time points following Aha labeling and chloramphenicol treatment were treated with tetramethylrhodamine alkyne (TAMRA-alkyne) under copper-catalyzed click conditions, and proteins were resolved by mass via SDS-PAGE for in-gel fluorescence detection (Fig. 2*A*). The stability of TAMRA-conjugated proteins over the observation window suggested these Aha incorporation levels are not broadly destabilizing, as previously observed in mammalian cells, and recapitulate observations that most nascent *E. coli* proteins are stable (19, 23).

To profile global protein degradation kinetics, we prolonged the observation window (0, 0.5, 1, 2, and 4 h) in a large-scale experiment and enriched labeled proteins after covalent binding to a dibenzocyclooctyne agarose (DBCO-agarose) resin. Enriched proteins were then digested and TMT labeled for liquid chromatography–tandem mass spectrometry (LC–MS/MS) analysis coupled with high-field asymmetric waveform ion mobility spectrometry (FAIMS) separation. We quantified 1,810 proteins and fit their normalized time-series abundances to an exponential model, extracting the rate constant as an estimator for proteolytic instability (Fig. 2*B* and Dataset S2). Density estimates of the rate constant distribution stratified by substrate annotation status derived from text mining as described earlier exhibited an expected negative skew consistent with a minority of proteins experiencing degradation. We identified an enrichment of annotated substrates (Fisher’s exact test; odds ratio = 4.8, *P*-value = 2.9 × 10^−10^) among a set of 88 proteins with pronounced instability in our screen (estimated half-life < 4 h; FDR-adjusted *P*-value < 0.05), suggesting our method identifies established protease substrates. Degradation rate constant estimates were moderately correlated with those of reported unstable proteins from two other analyses of exponential growth cells in minimal medium (*SI Appendix*, Fig. S3). Importantly, we did not identify a bias toward periplasmic proteins among substrate candidates in our analysis as reported in a recent proteome-wide analysis that evaluated a chloramphenicol treatment (*SI Appendix*, Fig. S4) (16). However, we note that chloramphenicol treatment elicited an elevation of ATP independent of treatment duration in ATP luminescence assays (*SI Appendix*, Fig. S5) as previously observed, which we expect may accelerate degradation rates of canonical substrates by enhancing AAA+ protease processivity but not artifactually expand substrate scope, as the latter effect has been suggested to arise from low ATP conditions (35, 36). Time-series abundance decreases were seen across the quantitative dynamic range, with many high-confidence candidate substrates exhibiting consistent chronological decreases in abundance (Fig. 2*C*).

### The c-di-GMP Phosphodiesterase PdeH Is Destabilized by ClpXP.

**Fig. 3.**
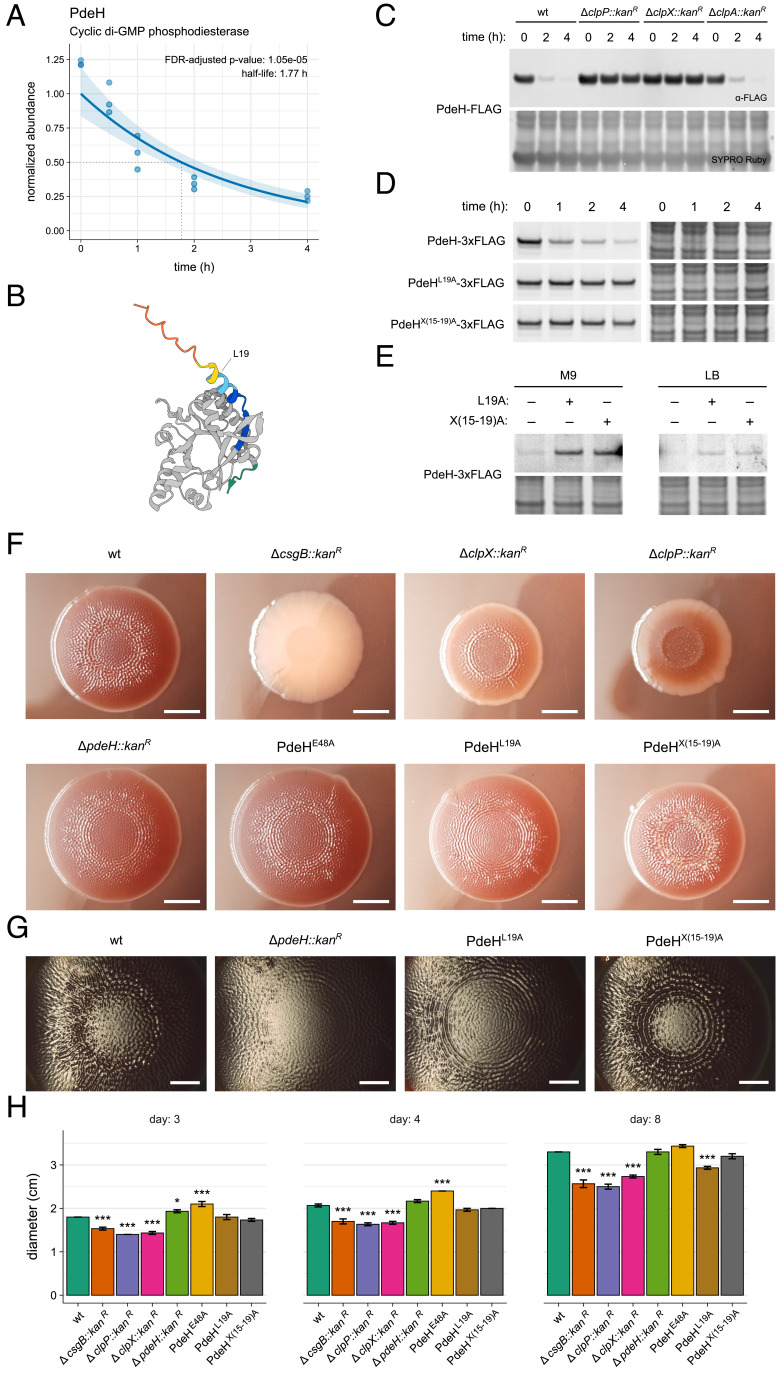
ClpXP-mediated degradation of PdeH. (*A*) Exponential fit of the PdeH abundance trace from our BONCAT analysis. The shaded region indicates a 95% CI for the predicted values. (*B*) AlphaFold2 approximation of PdeH structure. The conserved c-di-GMP EAL domain is shaded in gray, and the NTE is colored by the prediction’s per-residue model confidence score. The yellow and orange regions indicate low and very low model confidence scores, respectively. The C terminus of PdeH is excluded from the EAL domain and labeled in green. (*C*) Immunoblotting stability analysis of PdeH-FLAG expressed from a plasmid (pBAD33) in exponential phase cells across wild-type or protease deletion mutant strains. Cells were collected at the indicated time point after induction of protein expression and subsequent treatment with spectinomycin. Full membranes with molecular weight annotations are reported in *SI Appendix*, Fig. S20*A*. (*D*) Immunoblotting stability analysis of wild-type or mutant PdeH-3xFLAG expressed from the *pdeH* locus in exponential phase cells. Cells were collected after the indicated timepoint after treatment with spectinomycin. Full membranes with molecular weight annotations are reported in *SI Appendix*, Fig S20*B*. (*E*) Immunoblotting of wild-type or mutant PdeH-3xFLAG expressed from the *pdeH* locus in stationary phase cells. Cells were cultured in M9 glycerol or LB medium. Full membranes with molecular weight annotations are reported in *SI Appendix*, Fig. S20*C*. (*F*) Representative macrocolony biofilms grown for three days from deletion mutant strains or strains bearing mutations to the chromosomal *pdeH* locus. Biofilm deposition is reported by Congo red staining. CsgB is essential for curli fiber nucleation. The E48A active site mutation disrupts the catalytic activity of PdeH. (Scale bar, 5 mm.) (*G*) Stereomicrographs of the representative macrocolony biofilms grown for three days from strains bearing a deletion or stabilizing mutation at the chromosomal *pdeH* locus. (Scale bar, 2.5 mm.) (*H*) Macrocolony biofilm diameters at the indicated day of observation. Bars report the mean diameter value for each strain (n = 3), and error bars report the SEM. Asterisks report statistical significance (*P-value < 0.05; **P-value < 0.01; ***P-value < 0.001; two-sided Student’s *t* test) for changes in macrocolony biofilm diameters relative to that of wild-type MG1655 on each day.

The second messenger cyclic di-GMP (c-di-GMP) exerts transcriptional, translational, and posttranslational regulation on its effectors to orchestrate diverse cellular processes such as motility, surface adhesion, virulence, and cell division in bacteria (37). In *E. coli*, c-di-GMP primarily influences motility and the production of the biofilm matrix components curli fiber and phosphoethanolamine cellulose, and the antagonistic activities of diguanylate cyclases (DGCs) and c-di-GMP phosphodiesterases (PDEs) determine steady-state c-di-GMP levels. We identified the PDE that exclusively licenses motility, PdeH, as a candidate substrate exhibiting a half-life of approximately 1.77 h (Fig. 3*A*). PdeH bears the simplest domain architecture of all 13 PDEs in *E. coli* MG1655, lacking accessory domains for localization or allosteric regulation and instead harboring solely a conserved EAL domain with an N-terminal extension (NTE) poorly predicted by the AlphaFold2 model (Fig. 3*B*) (38, 39). To validate the instability of PdeH, we inserted its coding sequence fused to the FLAG epitope at either terminus into a medium-copy expression plasmid for immunoblotting stability analysis (40). The presence of a C-terminal ssrA-like tripeptide (LAL) in PdeH suggested proteolysis may initiate at this terminus. However, either blocking the N terminus with a FLAG tag or introducing successive N-terminal truncations stabilized PdeH, suggesting its N terminus mediates protease recognition (*SI Appendix,* Fig. S6 *A* and *B*). Ectopically expressed PdeH-FLAG in wild-type MG1655 cells exhibited degradation that was nullified in *clpP* and *clpX* backgrounds, establishing PdeH as a client of ClpXP (Fig. 3*C*).

As a class III gene in the flagellar regulatory cascade, *pdeH* is transcriptionally activated by the ClpXP substrate and master regulatory complex FlhDC, thus rendering ClpXP a regulator of both synthesis and degradation of PdeH (41–44). We imagined that the identification and subsequent mutagenesis of ClpXP recognition determinants in PdeH would allow us to isolate the role of proteolysis in shaping the endogenous PdeH abundance. Therefore, we subjected residues 2 to 21 of PdeH to alanine scanning or alanine stretch mutagenesis. The L19A point mutant and the X(8-13)A and X(15-19)A stretch mutants exhibited near-complete stabilization, further suggesting that the NTE confers ClpXP recognition (*SI Appendix,* Fig. S7).

To ascertain the physiological relevance of this proteolytic recognition, we used a scarless Cas9 cloning system to generate strains bearing stabilizing mutations in addition to a C-terminal 3xFLAG tag at the *pdeH* locus (45). PdeH-3xFLAG at endogenous expression levels exhibited instability consistent with our proteomic analysis, whereas the L19A and X(15-19)A mutants were completely stabilized (Fig. 3*D*). As a decrease in stationary phase PdeH abundance drives sessility and expression of the central biofilm regulator CsgD, we then monitored the abundance of PdeH in stationary phase (46). Cells bearing an L19A mutation at the *pdeH* locus exhibited an elevated abundance of PdeH in stationary phase when cultured in M9 or LB media (Fig. 3*E*). We conclude that the PdeH N terminus is a destabilizing regulatory motif that facilitates ClpXP-mediated depletion of this enzyme at growth arrest.

To evaluate whether PdeH destabilization influences an observable phenotype, we then constructed strains bearing single mutations to the *pdeH* locus without a C-terminal 3xFLAG tag and grew them as macrocolony biofilms on salt-free LB agar supplemented with Congo red to report curli fiber deposition (Fig. 3*F* and *SI Appendix,* Fig. S8). The MG1655 strain produced biofilms consisting of an outer growth zone and interior ridges and wrinkles characteristic of *E. coli* K-12 macrocolonies, whereas the strain carrying a deletion of *csgB*, essential for the nucleation of curli fiber, was devoid of surface morphology and curli deposition (47). In line with the role of ClpXP in regulating growth arrest, *clpP* and *clpX* deletion strains exhibited gross defects in curli fiber deposition and surface morphology. Deletion of PdeH or disruption of its catalytic activity through an E48A mutation to the conserved EAL domain active site residue elicited finer ridges and wrinkling, consistent with observations that PdeH deletion predominantly affects surface morphology in macrocolony biofilms (46, 47). The stabilizing L19A or X(15-19)A mutations induced defined, compacted rings and a roughened surface, respectively, visible in stereomicrographs along with alterations in the relative size of the flat inner zone of the biofilms and, in the case of L19A, a reduced macrocolony size (Fig. 3 *G*-*H* and *SI Appendix,* Fig. S9). However, we note that these effects were limited in comparison to those elicited by *clpX* or *clpP* deletion. While destabilization of PdeH may modestly influence macrocolony biofilm development, these results more broadly demonstrate that proteolytic regulation by ClpXP is essential for achieving canonical architectural features during macrocolony biofilm maturation.

### The ClpAP Modulator and N-Degron Recognin ClpS Is Degraded by ClpAP.

**Fig. 4.**
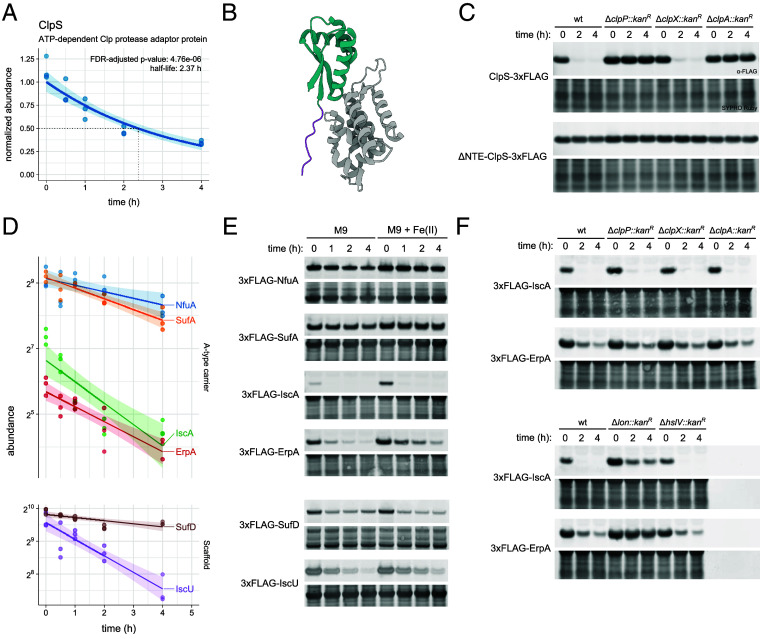
Degradation of the N-degron recognin ClpS and Fe-S cluster assembly components. (*A*) Exponential fit of the ClpS abundance trace from our BONCAT analysis. The shaded region indicates a 95% CI for the predicted values. (*B*) Crystal structure of ClpS (green) bound to ClpA (gray) (53). Residues 16 to 25 of the unstructured ClpS NTE (purple) are indicated. (*C*) Immunoblotting stability analysis of ClpS-3xFLAG and ∆NTE-ClpS-3xFLAG expressed from a plasmid (pBAD33) in exponential phase cells. Cells were collected at the indicated time point after induction of protein expression and subsequent treatment with spectinomycin. Full membranes with molecular weight annotations are reported in *SI Appendix*, Fig. S21. (*D*) Exponential fits of abundance traces from our BONCAT analysis for A-type carriers NfuA, ErpA, SufA, and IscA (*Upper* panel) and scaffold components IscU and SufD (*Lower* panel). The shaded regions indicate a 95% CI for the predicted values. (*E*) Immunoblotting stability analysis of substrate candidates bearing an N-terminal 3xFLAG tag expressed from a vector (pBAD33) in exponential phase cells grown with or without addition of 20 µM FeSO_4_ to the growth medium. Cells were collected at the indicated time point after induction of protein expression and subsequent treatment with spectinomycin. Full membranes with molecular weight annotations are reported in *SI Appendix*, Fig. S22. (*F*) Immunoblotting stability analysis of 3xFLAG-IscA and 3xFLAG-ErpA expressed from a vector (pBAD33) in exponential phase cells across wild-type or protease deletion mutant strains. Cells were collected at the indicated time point after induction of protein expression and subsequent treatment with spectinomycin. Full membranes with molecular weight annotations are reported in *SI Appendix*, Fig. S23.

The N-end rule pathway causally links N-terminal residue identity to proteolysis in bacteria and eukaryotes (48, 49). We observed degradation of the highly conserved N-degron recognin ClpS with a half-life of approximately 2.37 h (Fig. 4*A*) (50). ClpS chaperones N-end rule substrates for ClpAP-mediated degradation and modulates ClpAP activity toward ssrA-tagged substrates, protein aggregates, and ClpA itself (3, 50). ClpS comprises a substrate- and ClpA-binding core domain preceded by an unstructured NTE that reportedly moderates the ATPase activity and ssrA selectivity of ClpA by entering the ClpAP pore as a degron mimic (Fig. 4*B*) (51–53). In contrast with the documented in vitro stability of ClpS in ClpAP degradation reactions, immunoblotting stability analysis, and protease mapping of ectopically expressed ClpS-3xFLAG demonstrated its in vivo ClpAP-mediated degradation (Fig. 4*C*) (50). Truncation of the NTE abolished degradation, suggesting engagement of the ClpAP pore by ClpS licenses its destruction. In support of this notion, double mutation of P24A and P25A in the ClpS NTE renders the protein susceptible to ClpAP-mediated degradation, suggesting certain conditions admit recognition of ClpS for ClpAP degradation (54).

### Fe-S Cluster Assembly Proteins are Differentially Stable.

*E. coli* stores approximately 30% of cellular iron in low-spin ferrous heme centers and prosthetic iron–sulfur (Fe-S) clusters, which are assembled on scaffold proteins and transferred to carrier proteins for allocation to their cognate apoproteins (55, 56). We observed instability across all four Fe-S cluster carrier proteins featuring the A-type domain, or A-type carriers (ATCs), including IscA, ErpA, SufA, and NfuA, the last of which bears an A-type domain with mutations at conserved Fe-S cluster–liganding cysteines (Fig. 4*D*). We also observed instability in two components of the ISC and SUF Fe-S cluster biogenesis pathway scaffold complexes, IscU and SufD, which are essential for the activity of their respective scaffolds.

We fused the 3xFLAG epitope to either terminus of these ATCs and scaffold proteins and inserted each fusion into a plasmid for ectopic expression for immunoblotting stability analysis. N-terminal 3xFLAG fusions reproduced the abundance rank order and differential stability observed in our proteomic analysis (*SI Appendix,* Fig. S10). While metal coordination is thought to influence protease engagement, the instabilities of IscA and the principal aerobic A-type carrier ErpA were independent of Fe^2+^ repletion (Fig. 4*E*) in M9 medium, which is deficient in iron (57–59). We selected these two ATCs for protease mapping and found partial and complete stabilization in a *lon* background for IscA and ErpA, respectively (Fig. 4*F*). We conclude that the A-type domain may endow a carrier with Lon recognition that is moderated by its sequence context.

### BONCAT Enables Global Protein Stability Profiling in Stationary Phase.

**Fig. 5.**
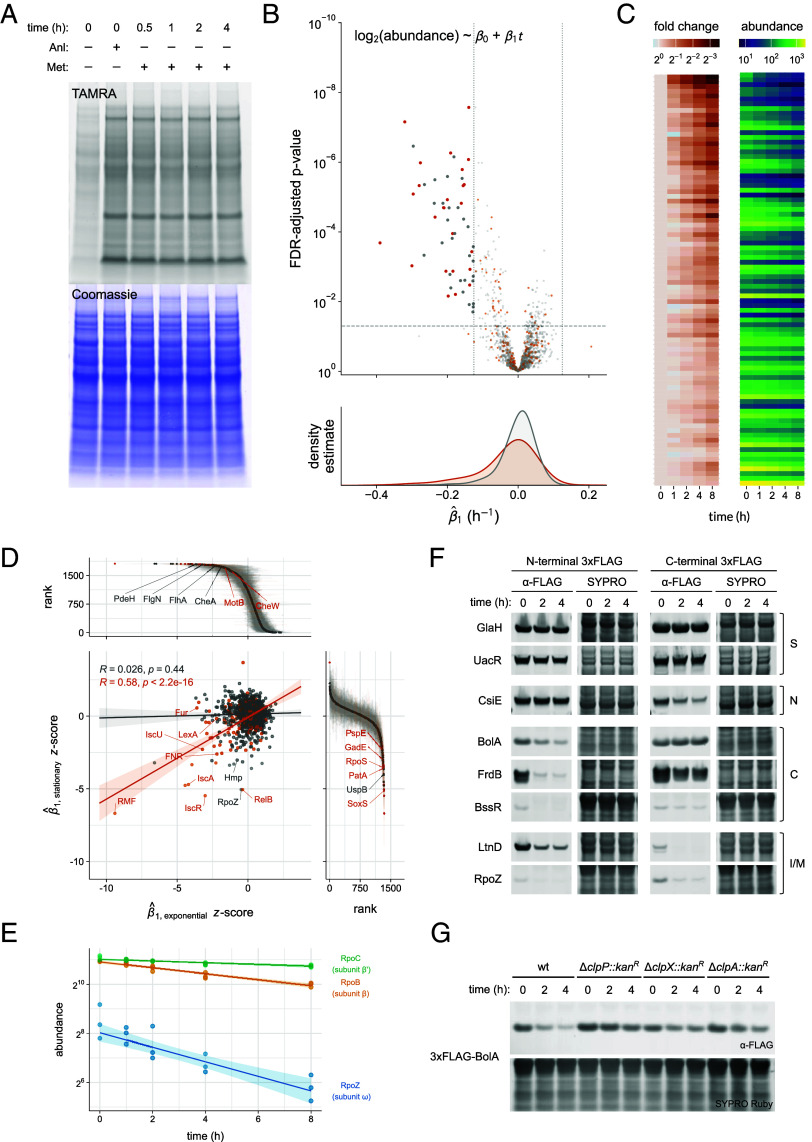
Global stability analysis in stationary phase cells reveals active degradation of stress response and catabolism proteins. (*A*) In-gel fluorescence of labeled proteins conjugated to TAMRA-alkyne. Cells treated with Anl for 4 h were collected at the indicated time point after a medium shift to spent medium supplemented with 1 mM Met. Coomassie staining reports total loading. (*B*) Statistical significance and kernel density estimates of fitted degradation rates (n = 1,339 proteins) stratified by annotation status. Vertical dotted lines indicate a rate constant that yields a fold change of two over the course of the experiment (estimated half-life < 8 h), and the horizontal line indicates an adjusted *P*-value < 0.05, with 25 annotated substrates and 31 unannotated substrates exceeding these thresholds. Orange shading represents annotated substrates, and gray shading indicates a lack of evidence of instability in the literature. (*C*) Heatmaps reporting mean abundances for proteins with evidence of exponential decay (estimated half-life < 8 h, FDR-adjusted *P*-value < 0.05, R^2^ > 0.5) in the screen with (*Left*) and without (*Right*) normalization to their initial abundance. (*D*) Joint plot comparing degradation rates across mutually identified proteins in the scatter plot panel. The marginal rate constant distributions report all quantified proteins in each condition with select exclusively identified proteins labeled by protein name. (*E*) Model fits for the subunits of RNAP exhibiting instability in the stationary phase screen. The shaded regions indicate a 95% CI for the predicted values. (*F*) Immunoblotting stability analysis of substrate candidates identified in the stationary phase screen bearing an N- or C-terminal 3xFLAG tag expressed from a vector (pBAD33) in exponential phase cells. Cells were collected at the indicated time point after induction of protein expression and subsequent treatment with spectinomycin. Candidates were assigned as stable (S) or unstable based on the potential presence of an N-degron (N), C-degron (C), or an internal degron or multiple degrons (I/M). The potential presence of an N-degron, C-degron, or internal degron was determined by differing effects of C- or N-terminal 3xFLAG tags on the fusion protein’s observed stability. Full membranes with molecular weight annotations are reported in *SI Appendix*, Fig. S24. (*G*) Immunoblotting stability analysis of 3xFLAG-BolA expressed from a vector (pBAD33) in exponential phase cells across wild-type and protease deletion mutant backgrounds. Cells were collected at the indicated time point after induction of protein expression and subsequent treatment with spectinomycin. Full membranes with molecular weight annotations are reported in *SI Appendix*, Fig. S25.

Having established the fidelity of BONCAT in identifying protease substrates in bacteria, we next leveraged the sensitivity of this method to conduct proteomic profiling of nascent protein stability in stationary phase, a model of microbial quiescence sharing gene expression commonalities with mature biofilms (60). We found that labeling stationary phase cultures with Aha produced insufficient methionine replacement for proteomic analysis on the basis of comparable intensity between Aha-labeled and unlabeled samples for many proteins in our in-gel fluorescence detection (*SI Appendix,* Fig. S11*B*). To achieve adequate labeling, we transcriptionally fused a stringently regulated ribosomal RNA promoter, *rrnB* P1, to a gene encoding NLL-MetRS to enable its accumulation during exponential phase for Anl labeling in stationary phase (61). Cells expressing the cassette from a low-copy plasmid were cultured for 24 h to stationary phase, pulse labeled with Anl for 4 h, resuspended in sterile-filtered spent medium collected from untreated cultures, and supplemented with Met as a chase treatment. We observed no growth resumption in stationary phase cultures upon treatment with Met or Anl with this protocol and did not detect a perturbation to viability during the observation window as assessed by an ATP luminescence assay (*SI Appendix,* Figs. S12 and S13). Lysates of cells collected during the chase were treated with TAMRA-alkyne under copper-catalyzed click conditions, and proteins were then resolved by mass with SDS-PAGE for in-gel fluorescence visualization (Fig. 5*A* and *SI Appendix*, Fig. S11*C*). We again observed widespread stability of the TAMRA-conjugated proteins visualized over the observation window, suggesting Anl, in a similar fashion to Aha, does not broadly destabilize recipient proteins at these incorporation rates (62).

We expanded this analysis for proteome-wide measurement by again scaling up this experiment and collecting cells over a prolonged observation window (0, 1, 2, 4, 8 h) for lysis. The Anl-labeled proteins were enriched, digested, and TMT labeled in an analogous manner to the exponential phase screen. Samples in this protocol were analyzed by the RTS-SPS-MS3 method to alleviate ratio compression arising from precursor ion cofragmentation (63). Analysis of the enriched peptides yielded 1,339 quantified proteins, and abundance traces again were fit to an exponential decay model for degradation rate constant estimation (Fig. 5*B* and Dataset S3). Degradation rates were generally slower in stationary phase than in exponential phase, consistent with reported differential instability measurements across a limited panel of model substrates (35). We again observed an enrichment of established protease substrates (Fisher’s exact test; odds ratio = 5.0, *P*-value = 7.0 × 10^−8^) among 56 candidates with pronounced evidence of instability (estimated half-life < 8 h; FDR-adjusted *P*-value < 0.05) along with chronological abundance decreases across the quantitative dynamic range for high-confidence candidates (Fig. 5*C*).

### Stationary Phase Substrates are Implicated in Stress Response and Catabolism.

Among candidates exhibiting instability in either growth phase, we observed modest overlap in the identities of annotated substrates, whereas unannotated substrate candidates were relatively distinct to each growth phase (*SI Appendix*, Fig. S14). Degradation rates for annotated substrates across these physiological states were correlated (Pearson’s *R* = 0.58), suggesting that both labeling strategies produce comparable measurements and that many established substrates are not conditionally unstable (Fig. 5*D*). Candidate substrates identified exclusively in the stationary phase dataset were often implicated in stress response, including the oxidative stress regulator SoxS and the canonical N-end rule substrate PatA, whereas those identified exclusively in exponential phase were associated with motility and flagellar assembly, which terminate in stationary phase (4, 46, 64). One of the most destabilized stationary phase candidates we observed was RpoZ, the nonessential ω subunit of RNA polymerase (RNAP) implicated in biofilm production and sigma factor selectivity (Fig. 5*E*) (65). In addition, we observed instability of subunits β and β’, which were previously identified as unstable in stationary phase (66).

We selected stationary phase substrate candidates for immunoblotting stability analysis in exponential phase cells as our attempts to establish a validation strategy in stationary phase cells revealed significant challenges in protein expression and protease assignment (Fig. 5*F* and *SI Appendix*, Fig. S15). Proteins involved in catabolism such as FrdB, CsiE, and LtnD exhibited instability in one or more fusions. We also identified instability in N-terminal 3xFLAG fusions of BolA, which mediates stationary phase morphology and was previously seen to be proteolytically unstable, and BssR, a regulator of biofilm production linked to quorum sensing and indole transport (67–69). Protease mapping suggested BolA may be recognized by multiple proteases including ClpXP, ClpAP, and Lon (Fig. 5*G* and *SI Appendix,* Fig. S16).

### Machine Learning–Assisted Substrate Identification Uncovers Proteolysis.

To address the abundance bias and physicochemical limitations that preclude full coverage in mass spectrometry–based proteomics, we sought to build and validate a predictive classification model for determining which proteins among those not identified in our proteomic profiling may also be subject to proteolytic regulation. Our training data consisted of 2,041 proteins quantified in our proteomic analyses, and 262 of these proteins bearing evidence of instability served as positive examples. A total of 9,794 distinct features were sourced from the UniProt, EcoCyc, STRING, and AlphaFold databases or computed from primary amino acid sequences (*SI Appendix*, Table S1).

Four models were considered for this classification task: logistic regression with L1 regularization, random forest, artificial neural network (ANN), and gradient-boosted logistic regression with XGBoost (70). Models were evaluated by subjecting training data to repeated nested cross-validation with stratification and sample weighting to address class imbalance, optimizing for mean average precision. On average, the XGBoost model achieved the highest mean average precision (0.359), representing a 2.8-fold increase over the baseline precision for this dataset (0.128), and the highest area under the curve (AUC) for the receiver operating characteristic (ROC) curve (0.746) (*SI Appendix,* Fig. S17). This represents an improvement over an existing model in discriminating unstable from stable proteins irrespective of degradation rate (ROC AUC ≈ 0.6) (15). We therefore selected the XGBoost model for its actionable performance and interpretability and used the fully trained model to assign probabilities of instability to 2,093 unseen proteins not identified in our proteomic analyses (Dataset S4). We note that training on a reduced set of 1,093 physicochemical attributes and predicted properties yielded reduced but nonetheless deployable performance, with the XGBoost model achieving the highest mean average precision (0.327), suggesting that this approach may generalize to less-annotated species (*SI Appendix,* Fig. S18).

**Fig. 6.**
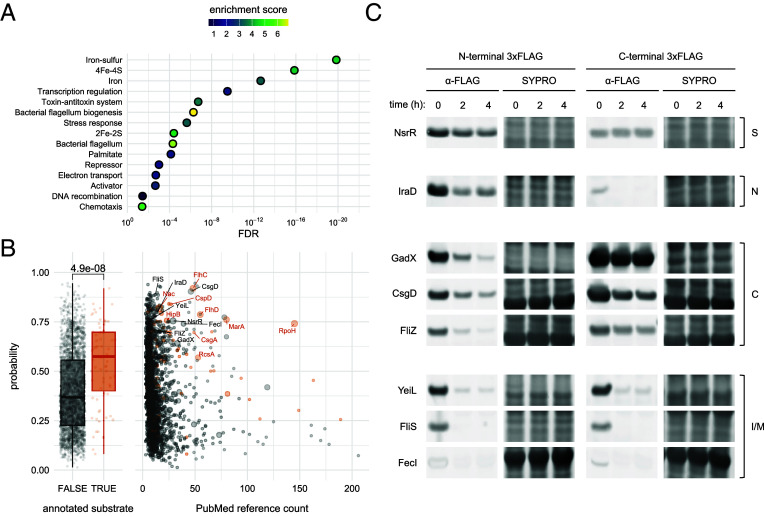
Prediction and validation of instability in unseen proteins. (*A*) Enriched UniProt keywords from competitive functional enrichment analysis of unseen proteins ranked by their assigned probability of instability. (*B*) Instability probabilities stratified by annotation status and PubMed reference count for proteins unseen by the model. Scatter point size in the second panel is proportional to the size of the indicated protein’s regulon. (*C*) Immunoblotting stability analysis of selected candidate substrates predicted as unstable by the model. Substrates were fused with the 3xFLAG epitope at either terminus and expressed from a medium-copy plasmid (pBAD33) in exponential phase cells. Cells were collected at the indicated time point after induction of protein expression and subsequent treatment with spectinomycin. Candidates were assigned as stable (S) or unstable based on the potential presence of an N-degron (N), C-degron (C), or an internal degron or multiple degrons (I/M). The potential presence of an N-degron, C-degron, or internal degron was determined by differing effects of C- or N-terminal 3xFLAG tags on the fusion protein’s observed stability. Full membranes with molecular weight annotations are reported in *SI Appendix*, Fig. S26.

We ranked unseen proteins by their assigned probability of instability for competitive enrichment analysis on the STRING database server, which yielded functionalities associated with proteolysis in the literature and in our proteomic profiling (Fig. 6*A*). Our model tended to assign higher probabilities to unseen proteins identified as protease substrates in the literature (two-sided Mann–Whitney U test; *P*-value = 4.9 × 10^−8^) than to proteins lacking documented evidence of instability in our curation, which were deemed on average as unstable or stable, respectively (Fig. 6*B*). Established protease substrates such as the heat shock sigma factor RpoH, antibiotic resistance regulator MarA, and FlhC and FlhD subunits of the FlhDC complex were assigned to the proteolytically unstable class by the model (42, 64, 71).

We then investigated the stability of unannotated predicted substrates chosen for their representation across predictive features and for their known biological functions. Immunoblotting stability analysis yielded time-series reduction in the abundance of two sigma factor regulators, IraD and FliS, which bind the RpoS adaptor RssB and the FliA anti–sigma factor FlgM, respectively; the iron-responsive extracytoplasmic sigma factor FecI; FNR homolog YeiL; a transcriptional activator of the acid resistance system, GadX; the RpoS antagonist and motility regulator FliZ; and the biofilm regulator CsgD, which drives surface adhesion by activating the production of curli fiber and phosphoethanolamine cellulose (Fig. 6*C*). The instability of IraD observed here supports a brief comment regarding proteolysis of the protein (72). The nitrite-sensitive repressor NsrR did not exhibit substantive time-series decay and was determined to be a predicted substrate that did not validate in our screen.

### Lon-Mediated Proteolysis of FliZ and CsgD.

**Fig. 7.**
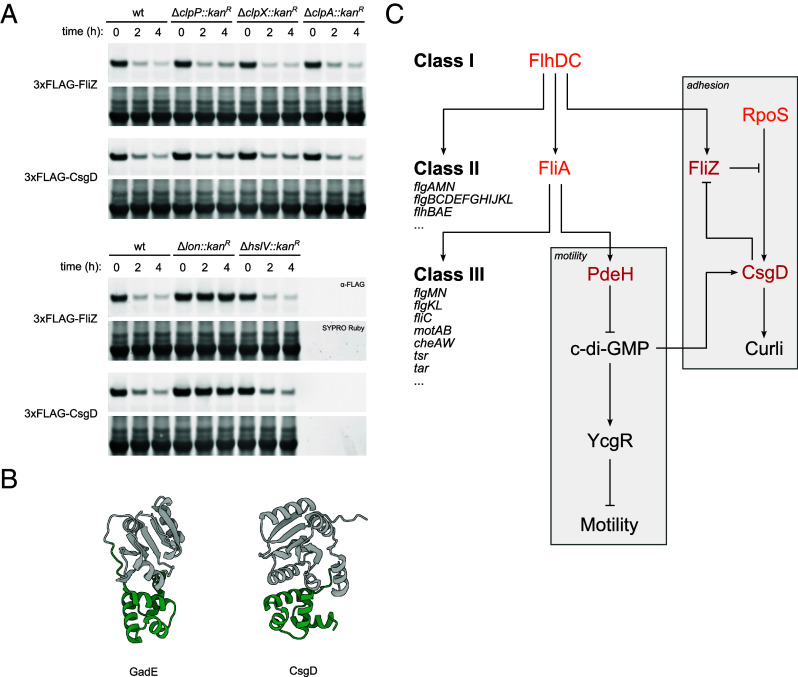
Lon-mediated proteolysis of FliZ and CsgD. (*A*) Immunoblotting stability analysis of 3xFLAG-FliZ and 3xFLAG-CsgD expressed from a vector (pBAD33) in exponential phase cells across wild-type and protease deletion mutant backgrounds. Cells were collected at the indicated time point after induction of protein expression and subsequent treatment with spectinomycin. Full membranes with molecular weight annotations are reported in *SI Appendix*, Fig. S27. (*B*) AlphaFold2 approximations of Lon substrate GadE and CsgD. The C-terminal LuxR-type helix–turn–helix domains are highlighted in green. (*C*) Simplified genetic circuit illustrating regulation of motility and surface adhesion, with newly identified protease substrates (red) and known substrates (orange) indicated.

Given the high representation of the flagellar regulatory cascade in our experimental and computational identifications, we selected CsgD and FliZ for protease mapping and found complete stabilization of these two substrates in a *lon* background (Fig. 7*A*). Notably, CsgD bears a C-terminal LuxR-type helix–turn–helix domain also found in Lon substrates RcsA and GadE (Fig. 7*B*) and is encoded by the most transcriptionally regulated operon (*csgDEFG*) described on the EcoCyc database, both being features deemed predictive by our model (*SI Appendix,* Fig. S19) (27).

A genetic analysis of the flagellar and curli expression cascades revealed that the class I master regulator FlhDC indirectly inhibits curli expression and maintains motility by driving expression of FliZ and PdeH as class II and class III gene products, thereby inhibiting expression of CsgD (46). Our results indicate all three of these regulators are actively degraded in vivo (Fig. 7*C*). Thus, we conclude that proteolytic degradation moderates the abundance of key regulators at each level of the flagellar gene cascade hierarchy, which may accelerate transitions between motility and sessility.

## Discussion

Much of the proteolytic degradation established here pertains to motility and c-di-GMP. As the timed destruction of PdeA drives cell cycle progression in *Caulobacter crescentus*, the regulated proteolysis of a c-di-GMP PDE is not unprecedented (73). A stabilizing point mutation in the N-terminal Per-ARNT-Sim domain of PdeA abrogated recognition by protease chaperone CpdR (74). The PdeH NTE is structurally and functionally uncharacterized, and our results do not exclude its potential role in mediating biomolecular interactions that influence ClpXP recognition of PdeH. In support of this prospect, the stabilizing L19A mutation is exceptionally far into the primary sequence relative to many other established ClpXP degrons (75). The differing effects of the L19A and X(15-19)A mutations on macrocolony biofilm physiology may further suggest the PdeH NTE may play unappreciated regulatory or functional roles. A comparative analysis identified absolute conservation of PdeH across 61 *E. coli* strains including both commensals and pathogens (38). Whether this proteolytic regulation operates and influences c-di-GMP in these and other *Enterobacteriaceae* members warrants further investigation. We also note that the PdeH-antagonizing DGC, DgcE, was reported to degrade in vivo, though the protease responsible for the observed instability could not be identified (76, 77). As c-di-GMP lies at the heart of many lifestyle changes and irreversible cell fate commitments, regulated proteolysis of PDEs and DGCs may prove to be a continuing theme in microbial physiology.

The *clpS* promoter is downregulated by low cytoplasmic Mg^2+^, and elevated ClpA abundance in stationary phase decreases the ClpS:ClpAP ratio, suggesting that ClpS abundance and relative stoichiometry are tuned to address stress (78, 79). Degradation of ClpS may contribute to this end, and its occurrence at both physiological and excess stoichiometries suggests proteolysis may proceed constitutively, though further study should probe any potential stabilizing effects of N-end rule substrate and ClpA binding. How ClpS may escape degradation by ClpAP has not been established in contemporary models across numerous biochemical, biophysical, and structural studies (52). The prospect of adaptor-substrate codegradation has been recognized as compatible with other mechanistic aspects of the ClpS-ClpAP system and is seen in the competence-regulating ClpCP adaptor MecA in *Bacillus subtilis* (80, 81). The conflicting outcomes of published in vitro and in vivo ClpS stability analyses may further suggest that an unconsidered variable or unidentified factor absent in certain reconstituted in vitro systems is involved in ClpS substrate exchange with ClpAP. Indeed, mutagenesis of the ClpS NTE has been demonstrated to elicit ClpAP recognition and degradation for this protein, suggesting some conditions may enable the destruction of ClpS by ClpAP (54). Furthermore, an unexplained in vivo stabilization of ssrA-tagged substrates against ClpAP was eventually attributed to the SspB and ClpS adaptors upon their discovery and characterization (2, 50, 82, 83).

Switching from the ISC to the SUF Fe-S biogenesis pathway has been proposed to drive antibiotic tolerance to aminoglycosides and fluoroquinolones through impaired assembly of respiratory complexes and enhanced assembly of oxidative stress response regulators, respectively (84, 85). As our exponential phase analysis reflects proteome remodeling upon aminoglycoside treatment, the differential instability of essential Fe-S scaffold complex subunits may contribute to killing dynamics during time-series viability measurements. More broadly, much remains to be learned about the relative activities of ATCs across physiological states and the role of proteolysis therein, as both the origin of iron in Fe-S assembly and its fate when bound to a proteolytically degraded carrier are not fully characterized (56). While ErpA is important for aerobic growth and thought to be a gatekeeping carrier by receiving assembled clusters from IscA, SufA, and NfuA, some notable apoproteins rely specifically on a single ATC (58, 86, 87). For example, the anaerobic transition regulator FNR relies exclusively on IscA for its Fe-S cluster (87). The role of proteolysis in determining the activities of the ISC or SUF assembly pathways, and thus the coordination status and proteolytic stability of their apoproteins, merits further analysis.

Our stability profiling of nascent proteins in stationary phase highlighted substrates associated with quiescence and catabolism, reinforcing the notion that proteolysis may accelerate physiological adaptation by eliminating specialized proteins when they are no longer needed. The most destabilized protein across both growth phases was ribosome modulation factor (RMF), which mediates the formation of inactive 100S ribosome dimers (88). This hibernation factor is subject to complex transcriptional and posttranscriptional regulation, with the *rmf* transcript being dramatically stabilized in stationary phase (89). Active degradation of RMF in growth-arrested cells may accelerate the recovery of translational capacity when growth-promoting conditions are introduced. We also observed instability of the catalytic subunits β and β’ and, most prominently, the nonessential ω subunit of RNAP. Deletion of ω perturbs biofilm formation in *E. coli* and other species, and the presence of this subunit reportedly influences sigma factor selectivity (90). Taken together, our observations suggest the role of proteolysis in shaping translational and transcriptional activity through these means may be of interest in further study of growth arrest.

Our analysis presented some notable themes in substrate attributes. The intrinsically disordered region of FtsZ contributes to its recognition and degradation by ClpXP, suggesting that an absence of defined structure may serve as a stability determinant as observed in eukaryotes (91, 92). Indeed, a recent analysis identified an enrichment of structural disorder among unstable proteins in *E. coli* (16). Some substrates in our analysis have explicitly described intrinsic disorder or conformational flexibility in the literature (e.g., ClpS and RpoZ) or alternatively possess termini that are poorly predicted by the AlphaFold2 model (e.g., PdeH and BolA), which is suggestive of intrinsic disorder (51, 93–95). Whether proteolytic degradation mediated by intrinsic disorder is leveraged for regulatory purposes may be contextual. The regulatory network also provided predictive power for several of the validated substrates. In particular, the number of transcriptional regulators mediating a given protein’s abundance served as a predictive feature, suggesting that a relationship exists between the degrees of transcriptional and posttranslational regulation governing a protein’s abundance. Furthermore, several proteins subject to active degradation in our proteomic profiling are elevated in abundance due to transposition events: differential expression of the catabolic galactitol operon genes (*gatYZABCD*) and FlhDC-regulated genes such as PdeH is well documented in comparative genetics and proteomics studies of *E. coli* K-12 lineages (96, 97). Proteolysis may in part attenuate or render dynamic the protein overexpression elicited by mobile genetic elements. We also note that the degradation rates for many proteins observed in our screen and others in *E. coli* are substantially longer than those often reported through in-vitro analysis, which suggests slow proteolysis may be an underappreciated mode of posttranslational regulation in this organism (15, 16, 49, 51). Such regulation may be important for adaptation to slow growth or growth-arrested states as the associated half-lives are significantly longer than the division times set by growth conditions in most studies. Indeed, the steady-state abundance of PdeH was not appreciably increased in exponential phase cells via stabilization (Fig. 3*D*).

We have demonstrated that stability profiling with the protein synthesis inhibitor chloramphenicol is a relatively simple method sufficient for global identification of known and novel protease substrates, with the caveat that elevated ATP from chloramphenicol treatment may accelerate degradation rates (35). While Aha labeling has proven effective in globally profiling protein degradation across multiple studies, this report also details application of Anl for time-series degradation profiling. We anticipate that this approach would similarly enable degradation profiling in other biological contexts distinguished by low translational activity. As expression of NLL-MetRS may be tied to predetermined transcriptional states, degradation profiling in defined cellular subpopulations could further elucidate differential instability of proteins across physiologies.

## Materials and Methods

Detailed descriptions of experimental procedures and data analysis are provided in *SI Appendix*, *Materials and Methods*.

### Text mining of PubMed and EcoCyc databases.

The Entrez Direct utilities were used to query PubMed for terms related to ATP-dependent protein degradation in *E. coli* (*SI Appendix*, Fig. S1). EcoCyc gene summaries were retrieved using the BioCyc web services API. Fetched PubMed abstracts or EcoCyc gene summaries were tokenized to sentences and words with the *tidytext* R package. Sentences containing query terms and an identified *E. coli* gene name were inspected in a specialized Shiny application (28). Publications with sentences describing proteolytic regulation were manually inspected for final approval of their corresponding reported annotations (Dataset S1). Protein–protein interaction and functional enrichment analysis of the 364 annotated substrates were performed on the STRING database server (98).

### Proteomic Analysis.

Chemoproteomic enrichment was performed as described in Glenn et al. (99). Enriched peptide samples were labeled with TMTpro (Thermo Fisher Scientific) for quantification by LC–MS/MS analysis.

### Bioinformatic Analysis.

All normalization and differential abundance analysis was conducted with the R package *limma* (100). Protein abundances were log_2_-transformed and normalized with the cyclic Loess method. Normalized log abundances were fit to a linear model for empirical Bayes–moderated *t*-testing of each coefficient with Benjamini–Hochberg adjustment of *P*-values for FDR control (101). Competitive functional enrichment analysis of ranked protein lists was performed on the STRING database server (98).

### Machine Learning.

Protein features were sourced from the UniProt, EcoCyc, STRING, and AlphaFold databases or computed with the Biopython package (*SI Appendix*, Table S1) (102). Degrons identified from proteomic profiling of ClpXP substrates were included as additional features (75). All proteins quantified across the exponential and stationary phase proteomics datasets were included as training examples, with proteins exhibiting statistically significant degradation rates (FDR-adjusted *P*-value < 0.05) and degradation rates verifiable by immunoblotting (log_2_ fold change < −0.5) in either dataset labeled as positive examples. Hyperparameter tuning and model evaluation were performed with 10 distinct rounds of nested cross-validation with 10 inner and outer folds, each stratified to maintain class imbalance. Hyperparameter values for grid searching during nested cross-validation were specified for each model class (*SI Appendix,* Table S2).

## Supplementary Material

Appendix 01 (PDF)

Dataset S01 (XLSX)

Dataset S02 (XLSX)

Dataset S03 (XLSX)

Dataset S04 (XLSX)

## Data Availability

LC-MS/MS proteomics data have been deposited in PRIDE (PXD062881) (103).
