# Supplementary material for: Global analysis of protein degradation reveals instability of diverse regulators in Escherichia coli
Source: Proc Natl Acad Sci U S A. 2026 Mar 3;123(10):e2515265123. doi: 10.1073/pnas.2515265123 (PMC12974527; doi:10.1073/pnas.2515265123)
Supplement: Supplementary file 1 — Appendix 01 (PDF) [file pnas.2515265123.sapp.pdf]

PNAS

# Supporting Information for

## Global analysis of protein degradation reveals instability of diverse regulators in *Escherichia coli*

Elliot J. MacKrell[1,**], Brett Lomenick[2], Yanping Qui[2,4], Hannah Jeckel[4], Jeff Jones[2,3], Tsui-Fen Chou[2,4], and David A. Tirrell[1,*]

[1]Division of Chemistry and Chemical Engineering, California Institute of Technology, Pasadena, CA, USA

[2]Proteome Exploration Laboratory, Beckman Institute, California Institute of Technology, Pasadena, CA, USA

[3]Division of Physics, Mathematics and Astronomy, California Institute of Technology, Pasadena, CA, USA

[4]Division of Biology and Biological Engineering, California Institute of Technology, Pasadena, CA, USA

**Current address: Xaira Therapeutics, South San Francisco, CA, USA

*David A. Tirrell, Elliot J. MacKrell.

**Email:** tirrell@caltech.edu ejmackrell@alumni.caltech.edu

**This PDF file includes:**

Supporting Text
Figures S1 to S29
Tables S1 to S5
Legends for Datasets S1 to S4
SI References

**Other supporting materials for this manuscript include the following:**

Datasets S1 to S4

## Supporting Information Text

## Materials and Methods

### Strain and plasmid construction

Gene deletion mutants were constructed with the Datsenko and Wanner method using template plasmid pKD13 and recombinase plasmid pKD46 as previously described (1). Kanamycin was used at 30 µg/mL for mutant selection. Deletions were verified by PCR and Sanger sequencing of the disrupted loci.

Genes encoding all substrates were amplified directly from the MG1655 genome. Genes encoding candidate substrates were inserted into a pBAD33 vector encoding the FLAG or 3xFLAG tags using Gibson assembly (2, 3). For evaluation of a stationary phase expression system for substrate validation, candidate substrates were amplified with primers bearing the 3xFLAG tag and inserted into pBbA2c using Gibson assembly (4). Vectors for alanine scanning and alanine stretch mutagenesis were constructed by site-directed mutagenesis with phosphorylated primers. Carbenicillin, kanamycin, spectinomycin, and chloramphenicol were used at 100 µg/mL, 50 µg/mL, 50 µg/mL, and 25 µg/mL, respectively, for plasmid maintenance where appropriate.

For chromosomal modification of the *pdeH* locus, mutation and epitope tag insertion with the Cas9 system from Jiang et al. were performed as described (5). The N20 sequence for the single guide RNA targeting *pdeH* was selected from a genome-scale library thresholded for strict off-target quality control with optimal CRISPR/Cas9 activity (6, 7). This sequence was inserted into pTarget with site-directed mutagenesis to construct pTarget-pdeH(N20). The donor DNA for constructing MG1655 *pdeH-3xFLAG* was assembled via Gibson assembly of fragments amplified from the MG1655 genome with primers bearing the 3xFLAG and PAM silencing mutations. The ligated product was inserted into pTarget-pdeH(N20) via Gibson assembly to construct pTarget-pdeH-3xFLAG. Site-directed mutagenesis was used to introduce the L19A mutation to this plasmid to construct pTarget-pdeH(L19A)-3xFLAG, which was then used to generate the MG1655 *pdeH(L19A)-3xFLAG* strain. The donor DNA for the alanine stretch mutant PdeH(X(15-19)A)-3xFLAG was constructed by amplification of pTarget-pdeH-3xFLAG with primers bearing the alanine stretch mutation followed by Gibson assembly of the reaction products. The ligated product was supplied as a fragment along with pTarget-pdeH(N20) for cotransformation to construct MG1655 *pdeH(X(15-19)A)-3xFLAG*. The donor DNA for PdeH(L19A) and PdeH(X(15-19)A) was constructed by Gibson assembly of fragments amplified from MG1655 *pdeH(L19A)-3xFLAG* and MG1655 *pdeH(X(15-19)A)-3xFLAG*. This donor DNA was supplied with pTarget-pdeH(N20) for cotransformation to construct MG1655 *pdeH(L19A)* and MG1655 *pdeH(X(15-19)A).* Primers encoding the E48A mutation were used to amplify a fragment of *pdeH* from pTarget-pdeH-3xFLAG that was then joined with the backbone of pTarget-pdeH-3xFLAG amplified without targeting the 3xFLAG sequence to construct pTarget-pdeH(E48A), which was then used to construct MG1655 *pdeH(E48A)*. All donor DNA incorporated 500 bp of homology flanking the desired mutation. Chromosomal insertion and sequence fidelity were verified by PCR and Sanger sequencing. The curing of pTarget and pCas was verified by recovery of antibiotic sensitivity to spectinomycin and kanamycin.

For construction of the PrrnBp1-dRBS-GFP-NLL-MetRS expression plasmid, a gene encoding 6xHis-NLL-MetRS was amplified from pBADP-NLL-MetRS (8) and inserted into pBAD33 using the KpnI and SalI restriction enzyme sites to create pBAD33-NLL-MetRS. Site-directed mutagenesis was then used to replace the 6xHis epitope with a 3xFLAG epitope and to introduce a Shine-Dalgarno sequence to construct pBAD33-SD-3xFLAG-NLL-MetRS. A gene encoding GFPmut3b was amplified from pUC18T-mini-Tn7T-PrpoS-gfp (8) and inserted into pBAD33-SD-3xFLAG-NLL-MetRS with Gibson assembly to construct pBAD33-SD-GFP-NLL-MetRS. A forward-designed ribosome binding site was then inserted with site-directed mutagenesis to construct pBAD33-dRBS-GFP-NLL-MetRS (9). The dRBS-GFP-NLL-MetRS fragment was then combined with the backbone of pBbS5k to construct pBbS5k-dRBS-GFP-NLL-MetRS using Gibson assembly (4). The expression promoter was then exchanged for the *rrnB* P1 promoter using site-directed mutagenesis to construct PrrnBp1-dRBS-GFP-NLL-MetRS.

**Media and growth conditions**

M9 glycerol medium consisted of 0.2% glycerol, 1 mM $MgSO_4$, 0.1 mM $CaCl_2$, 3 g/L $KH_2PO_4$, 0.5 g/L NaCl, 6.78 g/L $Na_2HPO_4$, and 1 g/L $NH_4Cl$ supplemented with 5 mg/L L-methionine and 40 mg/L of the remaining canonical amino acids. LB consisted of 10 g/L tryptone, 5 g/L yeast extract, and 10 g/L sodium chloride. NaCl-free LB agar consisted of 10 g/L tryptone, 5 g/L yeast extract, 18 g/L Bacto Agar (Difco) and was supplemented with 40 µg/mL Congo red and 20 µg/mL Coomassie Brilliant Blue G. Growth conditions were 37 °C at 250 RPM in an orbital shaker unless otherwise noted.

**In-gel fluorescence detection**

For the exponential phase in-gel fluorescence analysis, M9 glycerol medium was inoculated with a glycerol stock of *E. coli* MG1655 for overnight growth at 37 °C. Cells were then diluted to an $OD_{600}$ of 0.025 in fresh M9 glycerol medium and grown at 37 °C to an $OD_{600}$ of 0.75. Cells were treated with 1 mM Aha for 30 min and then treated with 100 µg/mL chloramphenicol. Cells were removed from the culture at the indicated time points, collected at 18000 RCF for 1 min, lysed in 0.5% SDS in PBS at 95 °C for 15 min, and stored at -20 °C for further processing.

For the stationary phase in-gel fluorescence analysis, M9 glycerol medium supplemented with 30 µg/mL kanamycin was inoculated from a glycerol stock to an $OD_{600}$ of 0.01, and cells were grown at 37 °C for 24 h. Cells were then treated with 1 mM Anl for 4 h. An untreated culture grown in parallel was centrifuged at 5000 RCF for 10 min, and supernatant was collected and sterile filtered to provide spent medium for the chase period. Anl-treated cells were collected at 5000 RCF for 10 min and resuspended in spent medium supplemented with 1 mM Met. Cells were then removed from the culture at the indicated chase time points, collected at 18000 RCF for 1 min, lysed in 0.5% SDS in PBS at 95°C for 15 min, and stored at -20 °C for further processing.

For dye conjugation, lysates were thawed, clarified for 20 min at 18000 RCF, and transferred to new microcentrifuge tubes. Protein concentrations were assessed with bicinchoninic assay (Thermo Scientific) and equalized with the addition of lysis buffer. Click reactions were performed as previously described with 10 µM TAMRA-alkyne, 250 µM $CuSO_4$, 1.25 mM tris(3-hydroxypropyltriazolylmethyl)amine (THPTA), 5 mM (+)-sodium L-ascorbate, and 5 mM aminoguanidine hydrochloride (10). Reactions proceeded for 1 h at 37 °C and were then combined with dithiothreitol (10X Reducing Agent, Invitrogen) and 4X LDS Fluorescence-Compatible Loading Dye (Invitrogen), heat treated at 70 °C for 10 min, and resolved by SDS-PAGE on 4-12% Bis-Tris NuPAGE gels (Invitrogen). Gels were destained in 10% acetic acid, 40% methanol, and 50% deionized water in the dark overnight. The next day, gels were rehydrated in deionized water and imaged on a Typhoon Trio (GE). Gels were then incubated in InstantBlue Coomassie Protein Stain (Expedeon) for at least 2 h and imaged again on a Typhoon Trio (GE).

**Chemoproteomic enrichment**

For the exponential phase enrichment, three 250 mL flasks containing 50 mL of M9 glycerol medium for each time point were inoculated with an overnight culture to an $OD_{600}$ of 0.01 and grown at 37 °C to an $OD_{600}$ of 0.75. Then, cultures were treated with 1 mM Aha for 30 min and subsequently with 100 µg/mL chloramphenicol. Cultures were removed from the incubator at their respective time points, centrifuged at 5000 RCF for 10 min, and then resuspended in 4% SDS in 100 mM Tris (pH 8.0) supplemented with cOmplete EDTA-free protease inhibitor (Roche) for lysis. Lysates were heat treated at 95 °C for 10 min and frozen at -80 °C for further processing.

For the stationary phase enrichment, six 250 mL flasks containing 55 mL of M9 glycerol medium supplemented with 30 µg/mL kanamycin were inoculated from individual frozen glycerol stocks to an $OD_{600}$ of 0.01 and grown at 37 °C for 24 h. Then, three cultures were treated with 1 mM Anl for 4 h. The remaining three cultures were centrifuged at 5000 RCF for 10 min, and supernatants were collected, pooled, and sterile filtered to provide spent medium for the chase period. Anl-treated cultures were centrifuged at 5000 RCF for 10 min, resuspended in 55 mL of spent medium supplemented with 1 mM Met, and returned to the incubator. At the indicated chase time points, 10 mL was removed from each

culture, centrifuged at 5000 RCF for 10 min, and resuspended in 4% SDS in 100 mM Tris (pH 8.0) supplemented with cOmplete EDTA-free protease inhibitor (Roche) for lysis. Lysates were heat treated at 95 °C for 10 min and frozen at -80 °C for further processing.

Thawed lysates were probe sonicated for 30 s at 20% amplitude (QSonica) with 2 s pulses and 1 s rests to shear chromosomal DNA. Lysates were then clarified by microcentrifugation at 18000 RCF for 20 min and transferred to new microcentrifuge tubes. Protein concentrations were assessed with bicinchoninic assay (Thermo Scientific) and equalized across samples with the addition of lysis buffer.

Lysates were brought to 500 µL by the addition of 1% SDS in PBS and then treated with 100 µL of 600 mM chloroacetamide in 0.8% SDS in PBS at 65 °C for 30 min in the dark at 1200 RPM on a thermoshaker. For each sample, 40 µL of dibenzocyclooctyne-agarose (DBCO-agarose) beads (Click Chemistry Tools) was washed thrice in 1 mL of 0.8% SDS in PBS and collected at 1500 RCF for 1 min. Lysates were then mixed with 600 µL of a solution of 8M urea and 0.85 M NaCl in PBS. Beads resuspended in 80 µL of 0.8% SDS in PBS were then added to each lysate and incubated at room temperature on a rotary wheel in the dark for 24 h.

Beads were then collected at 1500 RCF for 1 min, washed with 1 mL deionized water, and collected again at 1500 RCF for 1 min. Supernatants were then discarded, and beads were resuspended in 500 µL of 5 mM dithiothreitol (DTT) (Millipore Sigma) in 0.8% SDS in PBS and incubated for 15 min at 70 °C shaking at 1200 RPM on a thermoshaker in the dark. Supernatants were then removed after collecting beads at 1500 RCF for 1 min, and beads were resuspended in 500 µL of 40 mM chloroacetamide (Millipore Sigma) in deionized water and incubated for 30 min in the dark at room temperature on a rotary wheel.

For washing, beads were transferred to a PolyPrep gravity column (Bio-Rad) and washed ten times with 5 mL of 0.8% SDS in PBS (wash solution A), ten times with 5 mL of 8M urea in 100 mM Tris-Base (pH 8) (wash solution B), and ten times with 5 mL of 20% acetonitrile in deionized water (wash solution C). Before the second wash in each wash series, columns were capped, and beads were incubated in the wash solution for 10 min, 30 min, and 10 min for solutions A, B, and C, respectively. Columns were then uncapped and subjected to the remaining washes in each series.

Beads were resuspended in 1.5 mL of 50 mM ammonium bicarbonate (Millipore Sigma) in a 10% acetonitrile and 90% deionized water solution, collected at 1500 RCF for 1 min, and concentrated to 100 µL. Lys-C was added to 0.5 ng/µL for 4 h of digestion at 37 °C in the dark on a thermoshaker set to 1200 RPM. Trypsin was then added to 1 ng/µL for digestion overnight at 37 °C in the dark on a thermoshaker set to 1200 RPM.

The next day, samples were mixed with 100 µL of 20% acetonitrile in deionized water, and beads were collected at 1500 RCF for 1 min. Supernatants were then transferred to centrifugation columns (Pierce). This process was repeated twice with an increased volume of 150 µL of 20% acetonitrile in deionized water. Peptide solutions were vacuum concentrated (SpeedVac) to dryness and resuspended in 20 µL of 0.2% formic acid in mass spectrometry–grade water. Samples were then desalted with C18 ZipTips (EMD Millipore) per manufacturer's instructions and submitted for TMT labeling and LC-MS/MS analysis.

**LC-MS/MS analysis**

Enriched peptide samples were labelled with TMTpro (Thermo Fisher Scientific) according to the manufacturer's protocol, quenched with 5% hydroxylamine for 15 min, pooled, and lyophilized to dryness. Samples were resuspended in 5% acetonitrile/0.5% TFA, desalted on Pierce C18 spin columns (Thermo Scientific #89870), and the eluted peptides dried by lyophilization. Peptides were then resuspended in 2% acetonitrile/0.2% formic acid for LC-MS analysis.

Liquid chromatography-mass spectrometry (LC-MS) analysis was carried out on an EASY-nLC 1200 (Thermo Fisher Scientific) coupled to an Orbitrap Eclipse mass spectrometer (Thermo Fisher Scientific) equipped with a Nanospray Flex ion source. 1 µg peptides were directly loaded onto an Aurora 25cm x

75µm ID, 1.6µm C18 column (Ion Opticks, Victoria, Australia) heated to 50°C. The peptides were separated with a 180 min gradient at a flow rate of 350 nL/min as follows: 2–6% Solvent B (11 min), 6-25% B (124 min), 25-40% B (45 min), 40-98% B (1 min), and held at 98% B (15 min). Solvent A consisted of 97.8 % $H_2O$, 2% acetonitrile, and 0.2% formic acid, and solvent B consisted of 19.8% $H_2O$, 80% acetonitrile, and 0.2% formic acid.

For FAIMS analysis, FAIMS was used alternating between 3 cycles at -35V, -50V, and -65V with cycle times of 1.2, 1.0, and 0.8 seconds respectively. MS1 spectra were acquired in the Orbitrap at 120K resolution with a scan range from 350-1600 m/z, an automatic gain control (AGC) target of 1e6, and a maximum injection time of 50 ms in Profile mode. Features were filtered for monoisotopic peaks with a charge state of 2-7 and minimum intensity of 2.5e4, with dynamic exclusion set to exclude features after the first observation for 45 seconds with a 5-ppm mass tolerance. HCD fragmentation was performed with fixed collision energy of 32% after quadrupole isolation of features using an isolation window of 0.5 m/z, an AGC target of 5e4, and maximum injection time of 86 ms. MS2 scans were then acquired in the Orbitrap at 50k resolution in Centroid mode with first mass fixed at 110.

For RTS-SPS-MS3 analysis, MS1 spectra were acquired in the Orbitrap at 120K resolution with a scan range from 350-2000 m/z, an AGC target of 1e6, and a maximum injection time set to Auto in Profile mode. Features were filtered for monoisotopic peaks with a charge state of 2-7 and a minimum intensity of 1e4, with dynamic exclusion set to exclude features after the first observation for 45 seconds with a 5-ppm mass tolerance. CID fragmentation was performed with collision energy of 35%, activation time of 10 ms, and activation Q of 0.25 after quadrupole isolation of features using an isolation window of 0.7 m/z, an AGC target of 1e4, and a maximum injection time of 45 ms. MS2 scans were then acquired in the ion trap at rapid rate in Centroid mode with normal mass range and Auto scan range mode. RTS using the *E. coli* K-12 MG1655 UniProt reference proteome (UP000000625) was performed with carbamidomethylation of cysteines and TMTpro modification of lysines and peptide N-termini set as static modifications while methionine oxidation and deamidation of asparagine and glutamine were set as dynamic modifications. The maximum number of missed cleavages was set to 1, maximum variable mods was set to 2, and FDR filtering was not enabled. TMT SPS MS3 mode was used, and maximum search time was set to 35 ms. Top-20 SPS matches were used with a precursor selection range of 300-2000 m/z, precursor ion exclusion of -50 to +5 m/z, and isobaric tag loss exclusion reagent set to TMTpro. MS3 scans were then performed in the Orbitrap at 50k resolution in Centroid mode with a scan range of 100-500 m/z, using an MS isolation window of 1.2 m/z and an MS2 isolation window of 2 m/z, followed by HCD fragmentation with a fixed collision energy of 55%. The MS3 AGC target was set at 2.5e5 and maximum injection mode set to Auto. The mass spectrometry proteomics data have been deposited to the ProteomeXchange Consortium via the PRIDE partner repository with the dataset identifier PXD062881.

Analysis of LC-MS data was performed in Proteome Discoverer 3.0 (Thermo Scientific). Raw files were searched using the SequestHT search algorithm and the UniProt *E. coli* K-12 MG1655 reference proteome (UP000000625). Percolator FDR thresholds were 0.05 (relaxed) and 0.01 (strict). Trypsin was set as the cleavage enzyme, and the missed cleavage tolerance was set to 2. Methionine oxidation was set as a dynamic modification. Precursor ion mass tolerance was set to 10 ppm. Fragment ion mass tolerance was set to 0.02 Da for HCD FTMS2 scans and 0.6 Da for CID ITMS2 scans. Static modifications were set as N-terminal TMTpro modification, TMTpro modification of lysine, and carbamidomethylation of cysteine. The minimum peptide length was set to 6, and peptide and protein FDRs were set to 0.05 (relaxed) and 0.01 (strict). The co-isolation threshold for reporter ion quantification was set to 50 with a minimum average signal to noise threshold of 10. For SPS MS3 quantification, the SPS mass match threshold was set to 65%.

**Immunoblotting**

For immunoblotting stability analysis of candidate substrates, overnight cultures of cells carrying a plasmid for expression of an epitope-tagged substrate inoculated from glycerol stocks were diluted to $OD_{600}$ 0.025 in fresh M9 glycerol medium. For screening the stability of Fe-S cluster biogenesis pathway substrates, the medium was supplemented with 20 µM $FeSO_4$ for the iron replete condition. Cultures were

grown at 37 °C to an $OD_{600}$ of 0.5 and then induced for substrate expression with the addition of 0.2% arabinose for 30 min. Spectinomycin was added to 200 µg/mL in the culture medium, and aliquots of cells were withdrawn from the culture at the indicated time points. Cells were collected at 18000 RCF for 1 min, lysed in 0.5% SDS in PBS, heat-treated at 95 °C for 15 min, and stored at -20 °C for further processing.

For screening the stability of PdeH produced from the endogenous locus, M9 glycerol medium was inoculated from a glycerol stock for overnight growth at 37 °C. The next day, cells were diluted to an $OD_{600}$ of 0.025 in fresh medium and grown at 37 °C to an $OD_{600}$ of 0.5. Cells were then treated with 200 µg/mL spectinomycin, and aliquots of cells were withdrawn from the culture at the indicated time points. Cells were collected at 18000 RCF for 1 min, lysed in 0.5% SDS in PBS, heat-treated at 95 °C for 15 min, and stored at -20 °C for further processing.

For screening the abundance of PdeH produced from the endogenous locus in stationary phase, M9 glycerol or LB medium was inoculated from a glycerol stock for overnight growth at 37 °C. The next day, cells were diluted to an $OD_{600}$ of 0.025 in fresh M9 glycerol or LB medium. Cells were incubated at 37 °C for 24 h, collected by centrifugation at 18000 RCF for 1 min, lysed in 0.5% SDS in PBS, heat-treated at 95 °C for 15 min, and stored at -20 °C for further processing.

Protein lysates were thawed, clarified for 20 min at 18000 RCF, and transferred to new microcentrifuge tubes. Protein concentrations were assessed with bicinchoninic assay (Thermo Scientific) and equalized with the addition of lysis buffer. Lysates were then mixed with dithiothreitol (10X Reducing Agent, Invitrogen) and 4X LDS Fluorescence-Compatible Loading Dye (Thermo Fisher), heat treated at 70 °C for 10 min, and resolved by SDS-PAGE on 4-12% Bis-Tris NuPAGE gels (Invitrogen). Proteins were transferred to nitrocellulose membranes (0.2-µm pore size, Thermo Fisher Scientific) with the iBlot 2 Dry Blotting System (Invitrogen). Protein loading was assessed by staining with SYPRO Ruby (Thermo Fisher) per manufacturer instructions and imaging the stained membranes on a Typhoon Trio (GE). Membranes were then blocked with 5% milk in PBST for 1 h and treated with anti-FLAG M2 antibody (Millipore Sigma) diluted 1:5000 overnight at 4 °C on a rocker. Membranes were washed in four consecutive rounds with 20 mL of PBST for 5 min each, treated with Goat anti-Mouse Alexa Fluor 647 antibody (Invitrogen #A32728) diluted 1:10000 for 1.5 h, washed again in four consecutive rounds with 20 mL of PBST for 5 min each, and then imaged on a Typhoon Trio (GE). The SYPRO Ruby signal reported in all figures corresponds to the same region of the membrane reported in the FLAG channel.

**Macrocolony biofilm assay**

For the macrocolony biofilm assay, cells from overnight cultures grown in M9 glycerol medium at 37 °C were diluted to an $OD_{600}$ of 1 in fresh M9 glycerol medium and then dispensed in 5 µL drops onto salt-free LB agar plates supplemented with 40 µg/mL Congo red and 20 µg/mL Coomassie Brilliant Blue. The inocula were air dried for 20 min, and plates were then sealed with Parafilm. Colonies were incubated at 30 °C for 3 days and then photographed using both a standalone digital camera and a digital camera coupled to a stereomicroscope for surface morphology assessment. The radius of the flat inner zone was determined by displaying the image in MATLAB and using the "drawcircle" function to manually annotate the flat area, which was visually distinguished from the outer colony zone by the absence of ridges. The order in which images were shown to the annotator was randomized to avoid unconscious bias.

**ATP luminescence assay**

For the exponential phase ATP luminescence and optical density analysis, M9 glycerol medium was inoculated with a glycerol stock of *E. coli* MG1655 for overnight growth at 37 °C. Cells were then diluted to an $OD_{600}$ of 0.025 in fresh M9 glycerol medium and grown at 37 °C to an $OD_{600}$ of 0.5. Cells were treated with 1 mM Aha for 30 min and then treated with 100 µg/mL chloramphenicol. At the indicated timepoints, cells were collected for OD600 measurement and ATP concentration assessment with the BacTiter-Glo viability assay (Promega) following manufacturer's protocols.

For the stationary phase ATP luminescence and optical density analysis, M9 glycerol medium supplemented with 30 µg/mL kanamycin was inoculated from a glycerol stock to an $OD_{600}$ of 0.01, and

cells were grown at 37 °C for 24 h. Cells were then treated with 1 mM Anl or 1 mM Met for 4 h. For each culture, an untreated culture grown in parallel was centrifuged at 5000 RCF for 10 min, and supernatant was collected and sterile filtered to provide spent medium for the chase period. Untreated, Anl-treated, or Met-treated cells were collected at 5000 RCF for 10 min and resuspended in spent medium supplemented with 1 mM Met. Cells were then removed from the culture at the indicated chase time points and collected for $OD_{600}$ measurement and ATP concentration assessment with the BacTiter-Glo viability assay (Promega) following manufacturer's protocols.

**Machine learning model training**

Average precision was used as the performance metric for model selection. Training for the L1-regularized logistic regression and random forest model classes was conducted with the scikit-learn Python module with balanced class weights to address class imbalance (11). Neural networks were constructed with PyTorch and evaluated with the scikit-learn API provided by the skorch Python library (12). As two or more hidden layers provided no performance improvement at the cost of computation time, only networks with one hidden layer were considered. The BCEWithLogitsLoss loss function was used with positive weighting given by the class ratio to address class imbalance, and the loss function was optimized with the Adadelta optimizer using a batch size of 128 (13). Max epochs were set to 20 with early stopping set to a patience window of 3 monitoring the validation loss function. XGBoost was operated in linear mode with positive instances weighted according to the class ratio to address class imbalance, and the model was evaluated with the scikit-learn API of the XGBoost Python package. Performance metrics were assessed with the scikit-learn model selection routines. ROC and PR curves were averaged across all evaluated test sets, and PR curves were interpolated. PubMed reference counts for each protein were acquired from the Full Report entries on NCBI Gene with the Entrez Direct utilities. SHAP values for feature importance were calculated as previously described for linear models (14).

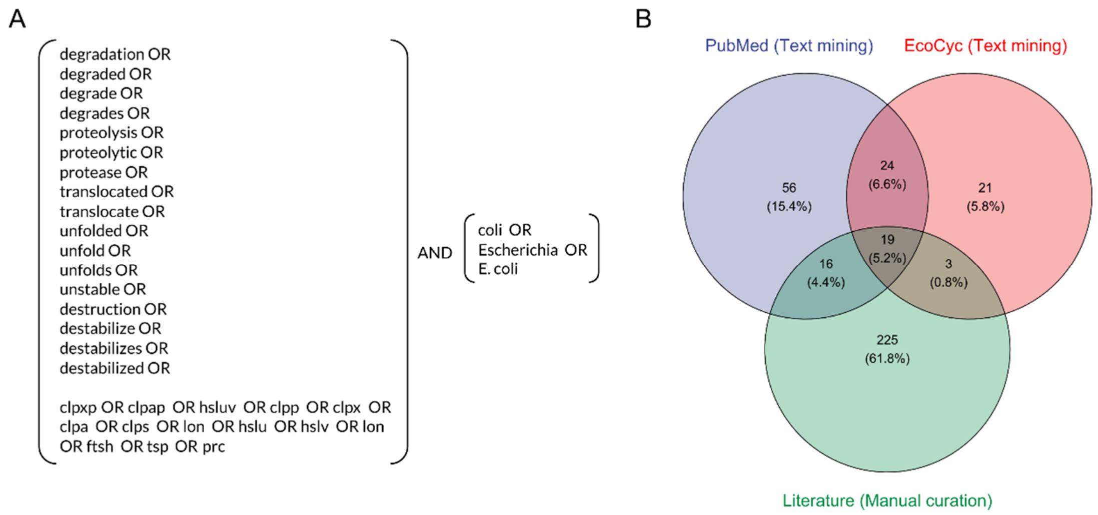


**Figure S1. Text mining of the PubMed and EcoCyc databases**. (A) Illustration of the Boolean query submitted to NCBI PubMed via the command line Entrez Direct utilities. The query yielded 25501 PubMed IDs that were then submitted to the PubMed2XL utility to retrieve article titles, abstracts, and metadata. This same query with the species specification removed was used to identify sentences among 4737 gene summaries of the EcoCyc database that may describe proteolytic regulation in *E. coli*. (B) Venn diagram depicting substrate annotations retrieved from the PubMed or EcoCyc databases via text mining or from manual curation of the research literature. Text mining grew the set of protease substrate annotations by 38%, with the mined knowledge bases providing distinct identifications.

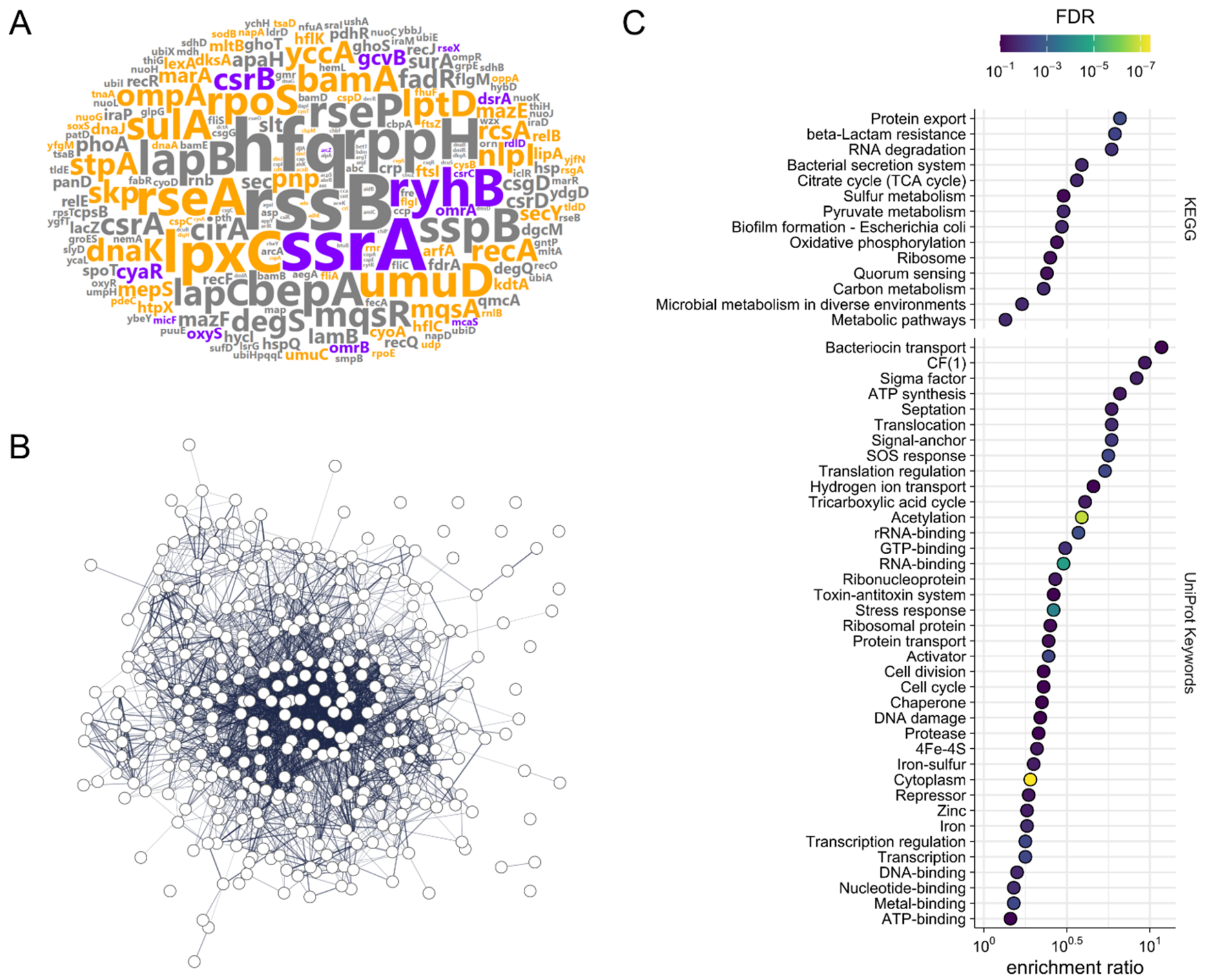


**Figure S2. Text mining of the PubMed and EcoCyc databases**. (A) Word cloud summarizing the gene counts across the query-satisfying sentences of the EcoCyc gene summaries. Gene names are scaled by their proportional representation. Orange names report annotated substrates, grey names indicate a lack of evidence of proteolytic degradation in the literature, and purple names report noncoding RNAs. (B) Protein-protein interaction (PPI) network for the 364 proteins bearing evidence of instability in the literature (observed number of edges: 3226; expected number of edges: 2029; PPI enrichment *p*-value < $1\times10^{-16}$). The expected number of edges was determined by the background interaction probability for all described *E. coli* MG1655 proteins on the STRING database server (15, 16). Edges indicate computational and experimental evidence of association. (C) Functional enrichment analysis in the UniProt and KEGG databases for annotated proteins. Enrichment is quantified as the ratio between the number of proteins described by the term in the network and the expected number of proteins described by the term across random networks of the same size.

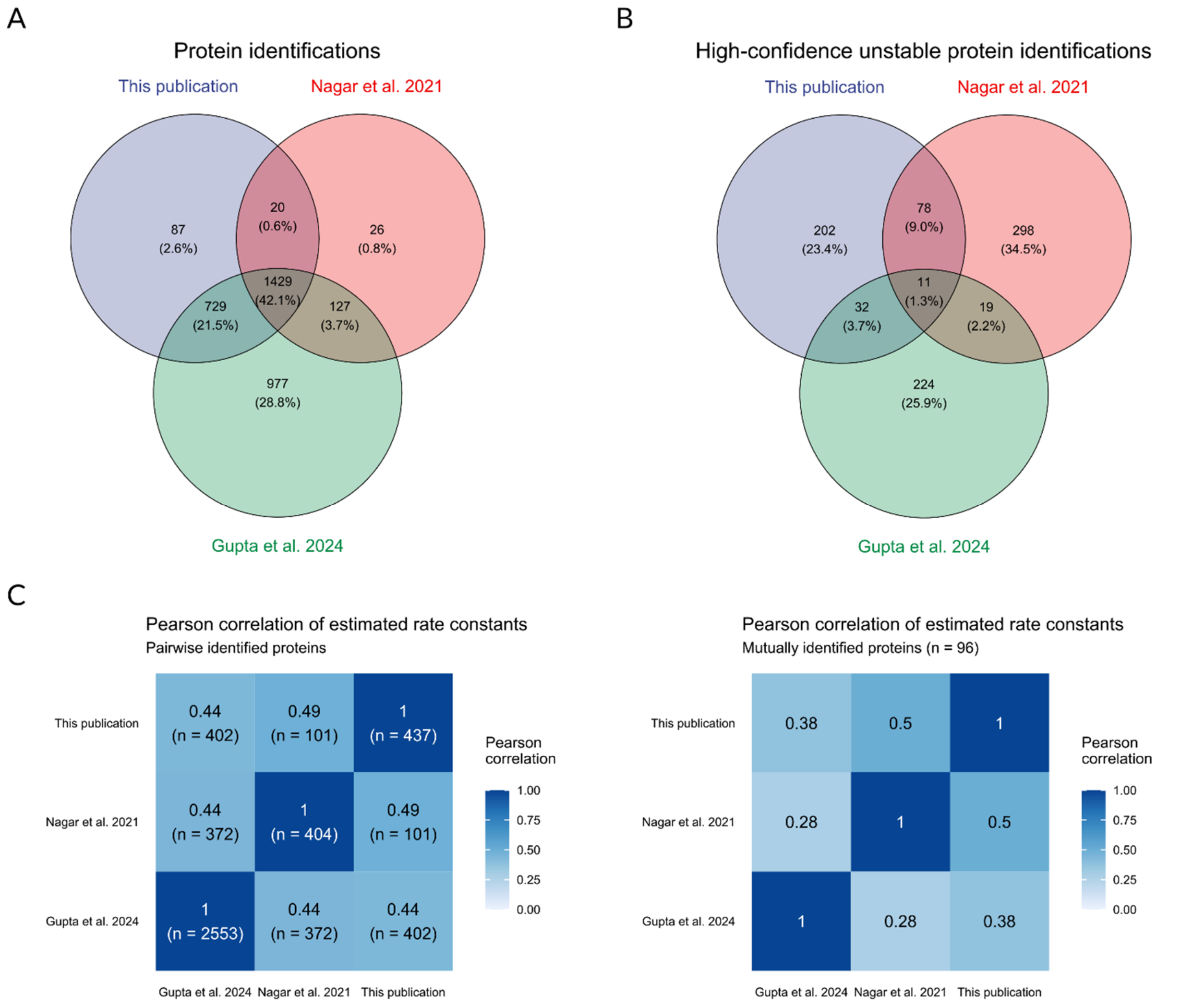


**Figure S3. Comparison to previously published kinetic analysis in *Escherichia coli*.** (A) Venn diagram of the protein identifications by UniProtKB accession ID from our study and from two previously published analyses. (B) Venn diagram of the high-confidence substrate identifications from statistical analysis of biological replicates by UniProtKB accession ID in our study and in two previously published analyses. For Nagar et al. comparisons, proteins reported as unstable (FDR-adjusted p-value < 0.05) by the authors were used. For Gupta et al. comparisons, proteins reported as confidently degrading from biological replicate analysis (one-sided *t*-testing with *p*-value < 0.05) by the authors were used. From this study, proteins exhibiting instability with FDR-adjusted p-value < 0.05 were used. Differences in experimental conditions, sample preparation, and computational methods deployed in one or more of the compared analyses may preclude broad consensus of substrate identities. (C) Pearson correlation coefficients of the rate constants for mutually identified unstable proteins between pairs of studies indicated by the x- and y-axes (left) or the mutually identified unstable proteins across all three studies (right). The rates reported for exponential phase cells in minimal medium were used from the Gupta et al. 2024 study and from this study (FDR-adjusted p-value < 0.05) to enable the most direct comparison between the three studies.

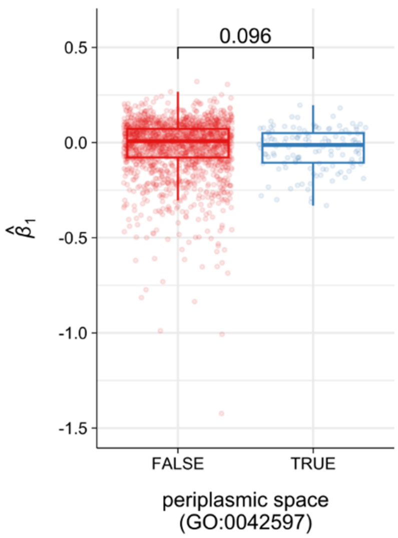


**Figure S4. Enrichment analysis for periplasmic proteins in the exponential phase degradation profiling.** Pulse labeling of exponential phase cells with Aha followed by treatment with 100 µg/mL chloramphenicol did not lead to an enrichment of this category of proteins among proteins exhibiting degradation in our proteomic profiling (two-sided Kolmogorov-Smirnov test; *p*-value = 0.096). We also did not observe an enrichment of periplasmic proteins among those exhibiting strong evidence of instability (estimated half-life < 4 h, FDR-adjusted *p*-value < 0.05) in the screen (Fisher's exact test; odds ratio = 0.79, *p*-value = 0.83).

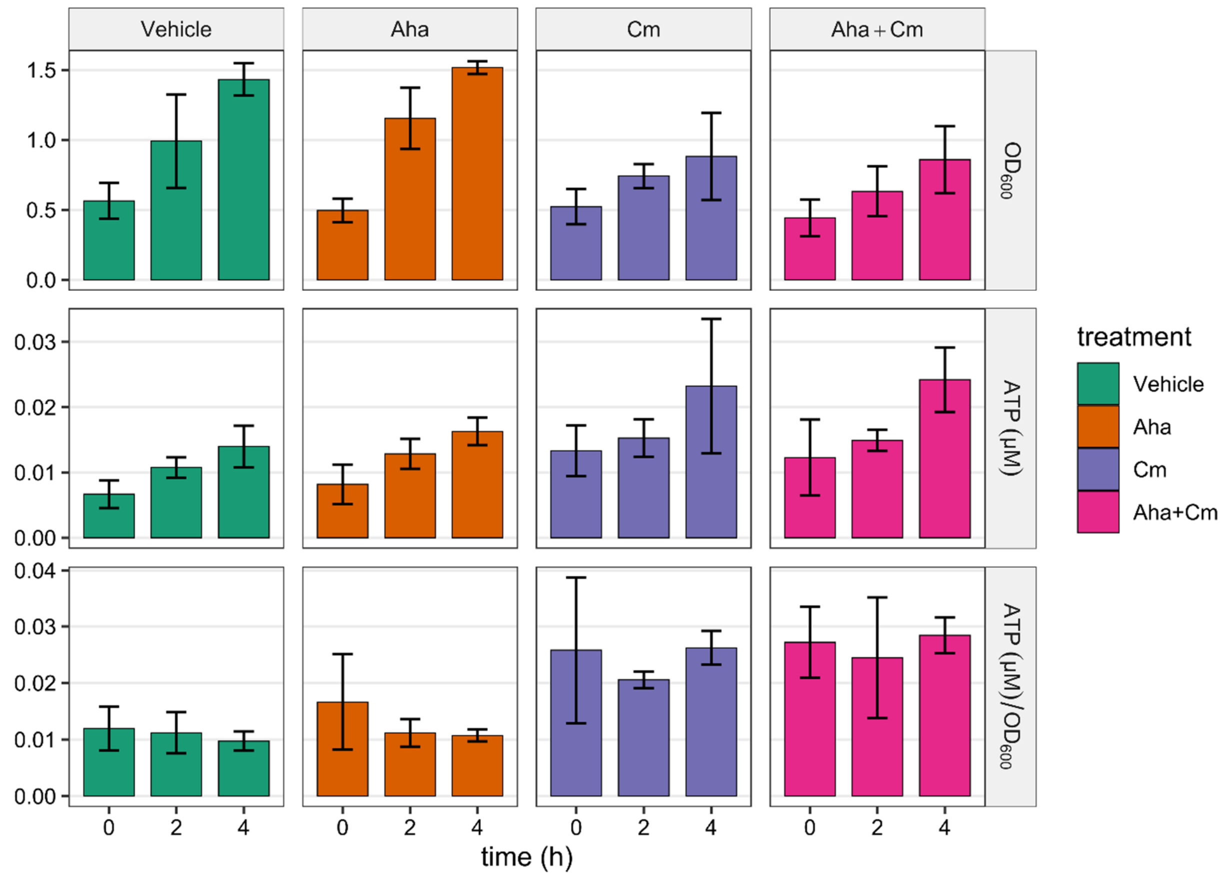


**Figure S5. Quantification of ATP in exponential phase cells treated with Cm or Aha.** Cells were cultured under the experimental procedures used in our exponential phase screen, subjected to the indicated treatment, and harvested at the indicated timepoints for cell density measurements and luminescence-based quantification of ATP. Bars report the mean value (n = 4), and error bars report 95% confidence intervals calculated using the *t*-distribution. When fitting a full factorial linear mixed-effects model to ATP normalized to $OD_{600}$ with time, Aha, and Cm covariates as fixed effects and replicate identifier as a random effect, we observe one significant treatment effect of a time-independent, general elevation for Cm-treated cells (β: 0.014 ± 0.002 ATP (µM) $OD_{600}^{-1}$, p-value = $4.22 \times 10^{-6}$; two-sided Student's *t*-test).

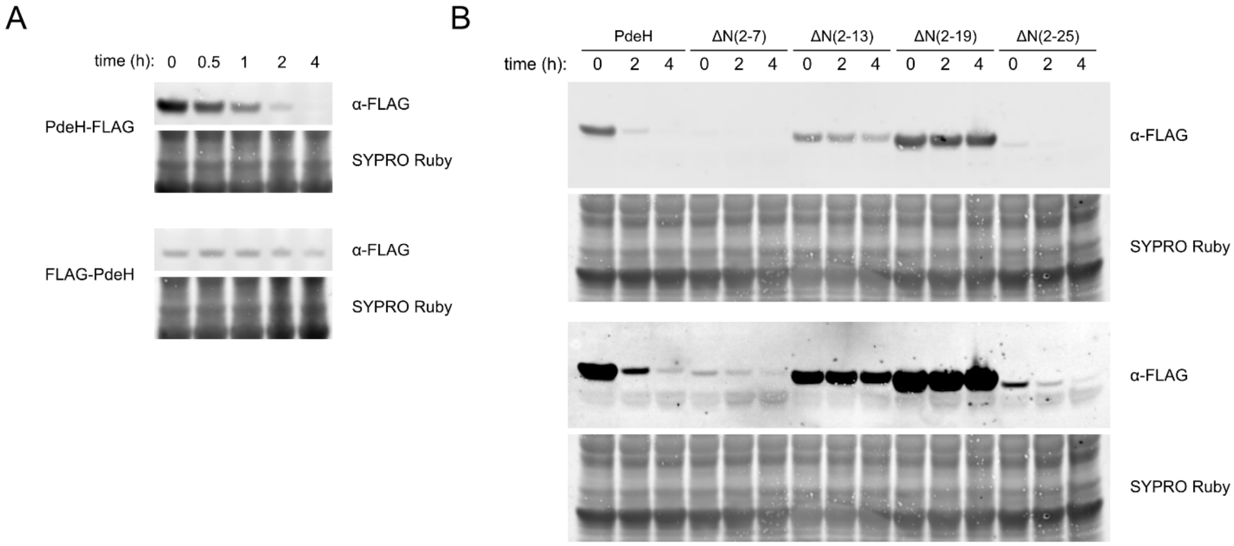


**Figure S6. Stability of FLAG-tagged PdeH and the N-terminal truncations of PdeH-FLAG.** (A) Immunoblotting stability analysis of PdeH-FLAG and FLAG-PdeH. While the C-terminal FLAG tag did not interfere with PdeH instability, blocking of the N terminus with the FLAG sequence stabilized PdeH. Expression of the N-terminal fusion may be lower due to altered translation initiation efficiency from sequence perturbation near the Shine-Dalgarno sequence. Full membranes with molecular weight annotations are reported in *SI Appendix,* Fig. S28A. (B) Immunoblotting stability analysis of PdeH-FLAG modified by introducing N-terminal truncations. Successive deletions of six-residue stretches in the PdeH N terminus yielded a stabilized PdeH-FLAG mutant with the removal of 12 or 18 amino acids. The bottom panel is the same blot with narrowed contrast for visualizing bands with reduced signal. Full membranes are reported in *SI Appendix*, Fig. S28B.

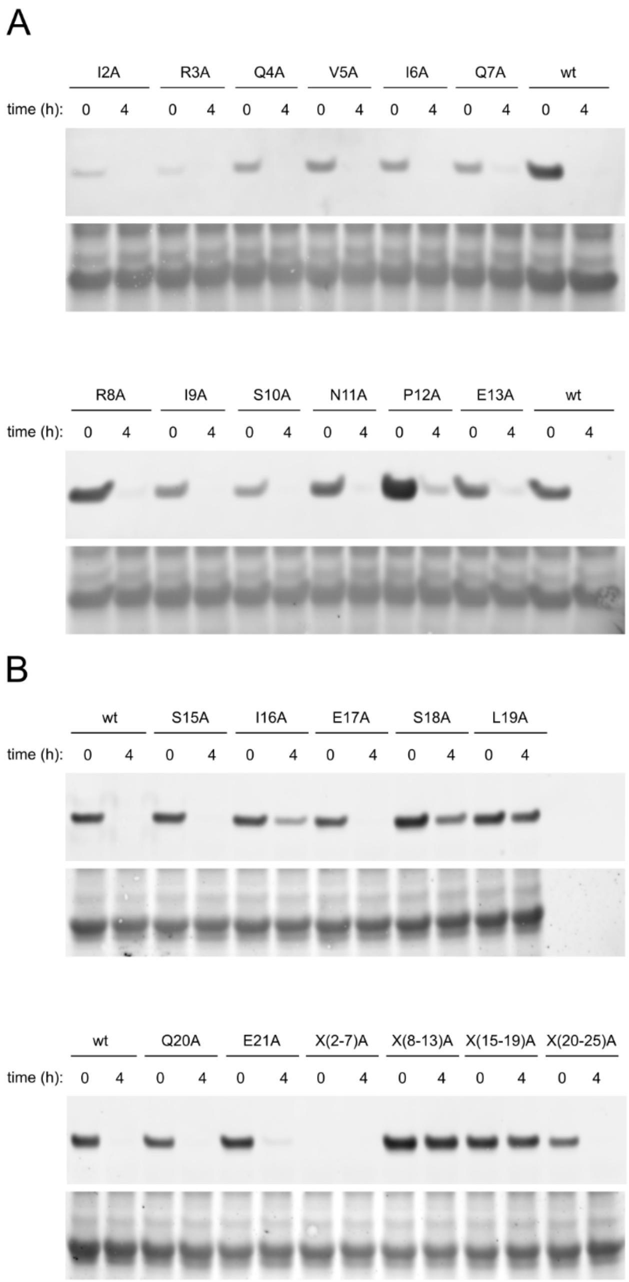


**Figure S7. Immunoblotting stability analysis of N-terminal alanine scanning and stretch mutants of PdeH-FLAG.** (A) Alanine scanning mutagenesis of residues 2-13 of the PdeH N terminus. Full membranes with molecular weight annotations are reported in *SI Appendix,* Fig. S29A. (B) Alanine scanning mutagenesis of residues 15-21 and alanine stretch mutagenesis of residues 2-7, 8-13, 15-19, and 20-25 of the PdeH N terminus. Alanine point or stretch mutation across the PdeH NTE yielded stabilized L19A, X(8-13)A, and X(15-19)A PdeH-FLAG mutants. Full membranes with molecular weight annotations are reported in *SI Appendix*, Fig. S29B.

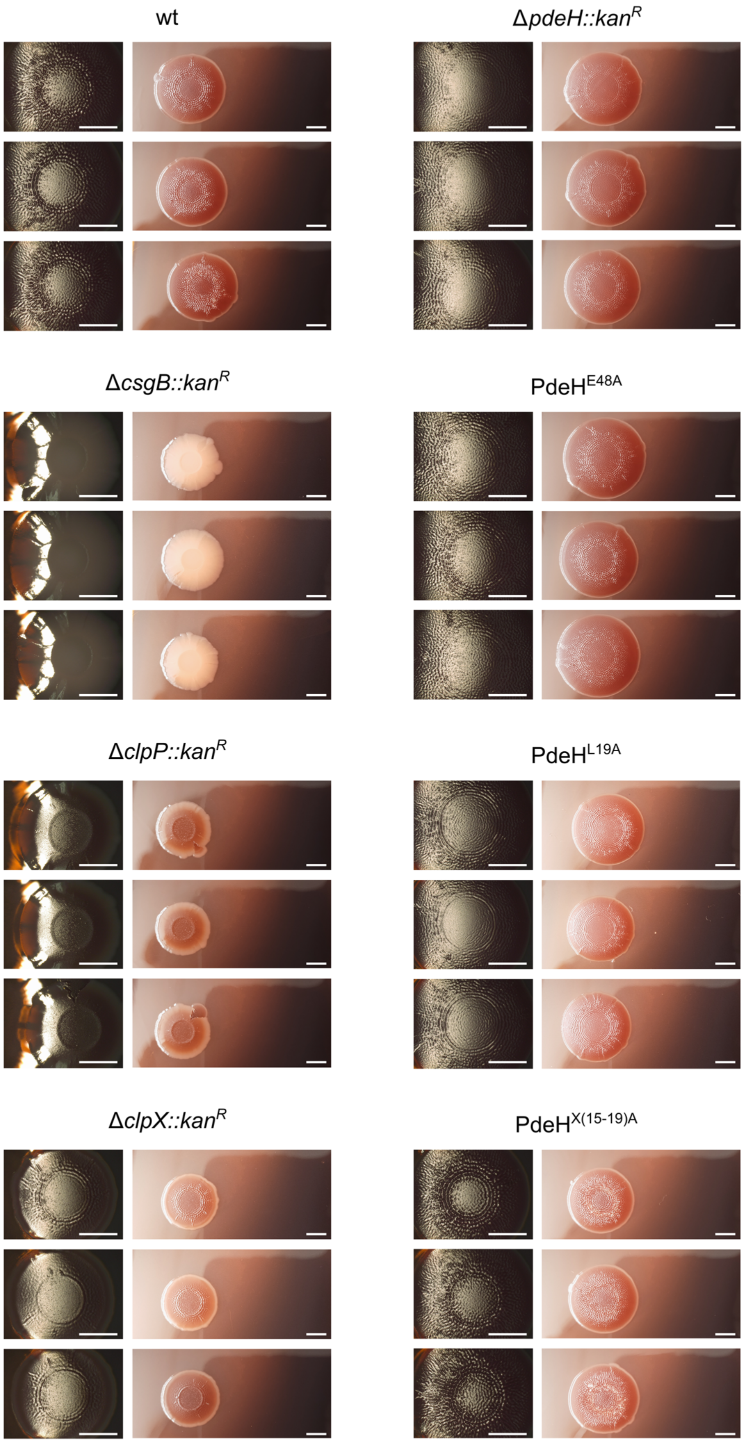


**Figure S8. Macrocolony biofilms of deletion and PdeH stabilization strains.** Photographs acquired by a digital camera with (left) or without (right) coupling to a stereomicroscope. Scale bar reports 5 mm.

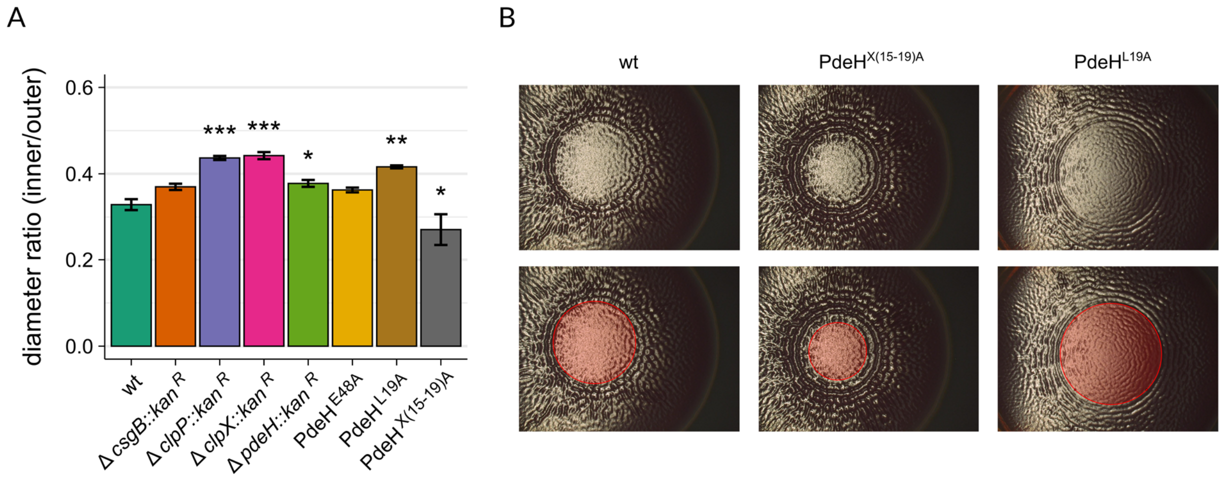


**Figure S9. Macrocolony diameter ratios for deletion and PdeH stabilization strains.** (A) Ratio of the inner diameter of the flat region of each macrocolony biofilm to the full diameter of the biofilm after three days of growth. Bars report the mean diameter value for each strain (n = 3), and error bars report the standard error of the mean. Asterisks report statistical significance (*p-value < 0.05; **p-value < 0.01; ***p-value < 0.001; two-sided Student's *t*-test) for changes in diameter ratios relative to that of MG1655 on each day. (B) Representative depictions of the region of interest defining the flat interior zone of the biofilms after three days of growth in stereomicrographs.

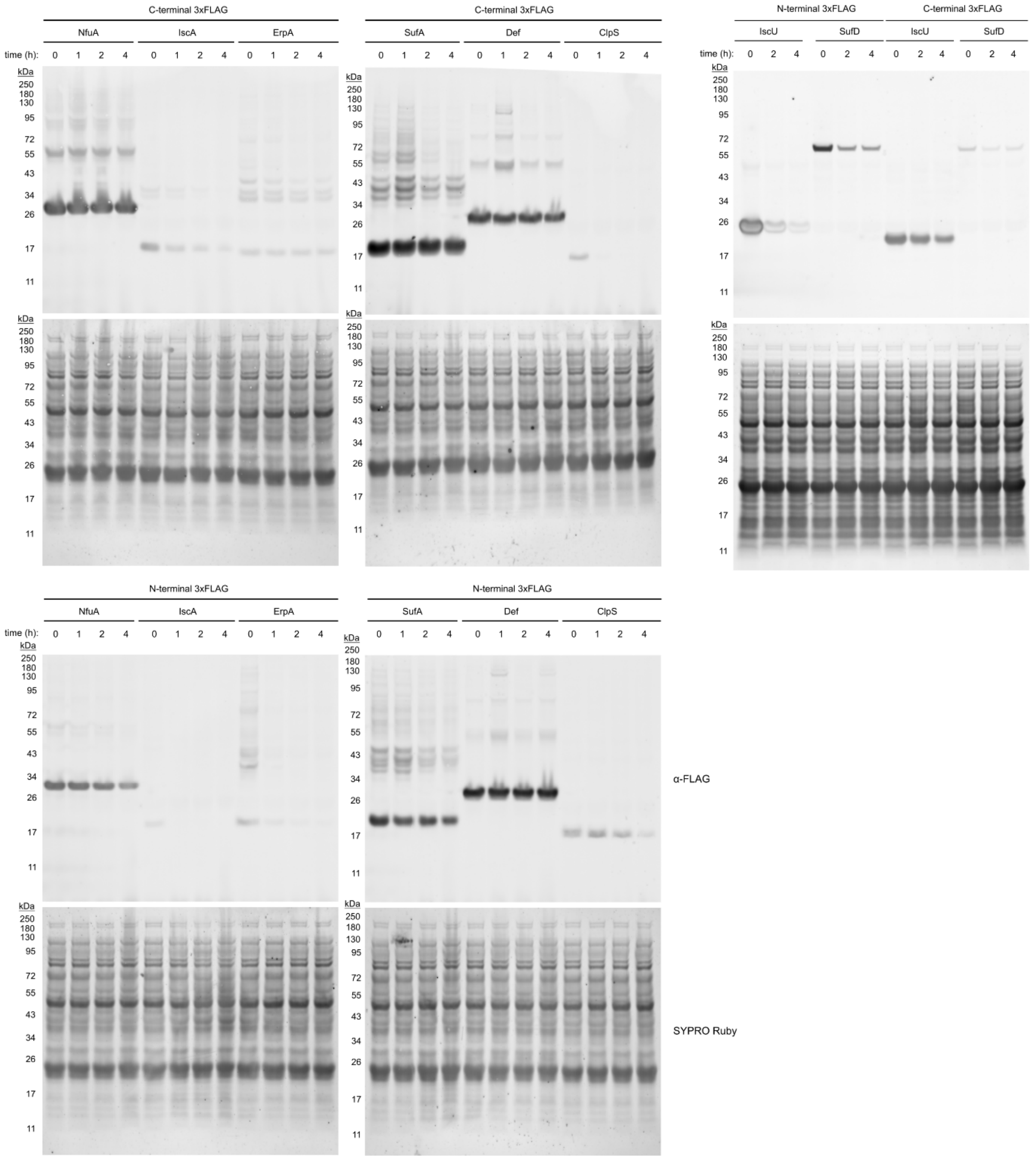


**Figure S10. Immunoblotting stability analysis of N- and C-terminally tagged Fe-S cluster biogenesis pathway proteins.** N-terminal 3xFLAG fusions recapitulated the proteolysis and rank order of expression for the A-type carriers IscA, ErpA, NfuA, and SufA and scaffold components SufD and IscU. The candidate substrate Def did not validate in this immunoblotting stability analysis. The ClpS-3xFLAG fusion displayed a degradation profile, whereas perturbation of the N-terminus of ClpS, which engages the ClpAP pore, stabilized the protein.

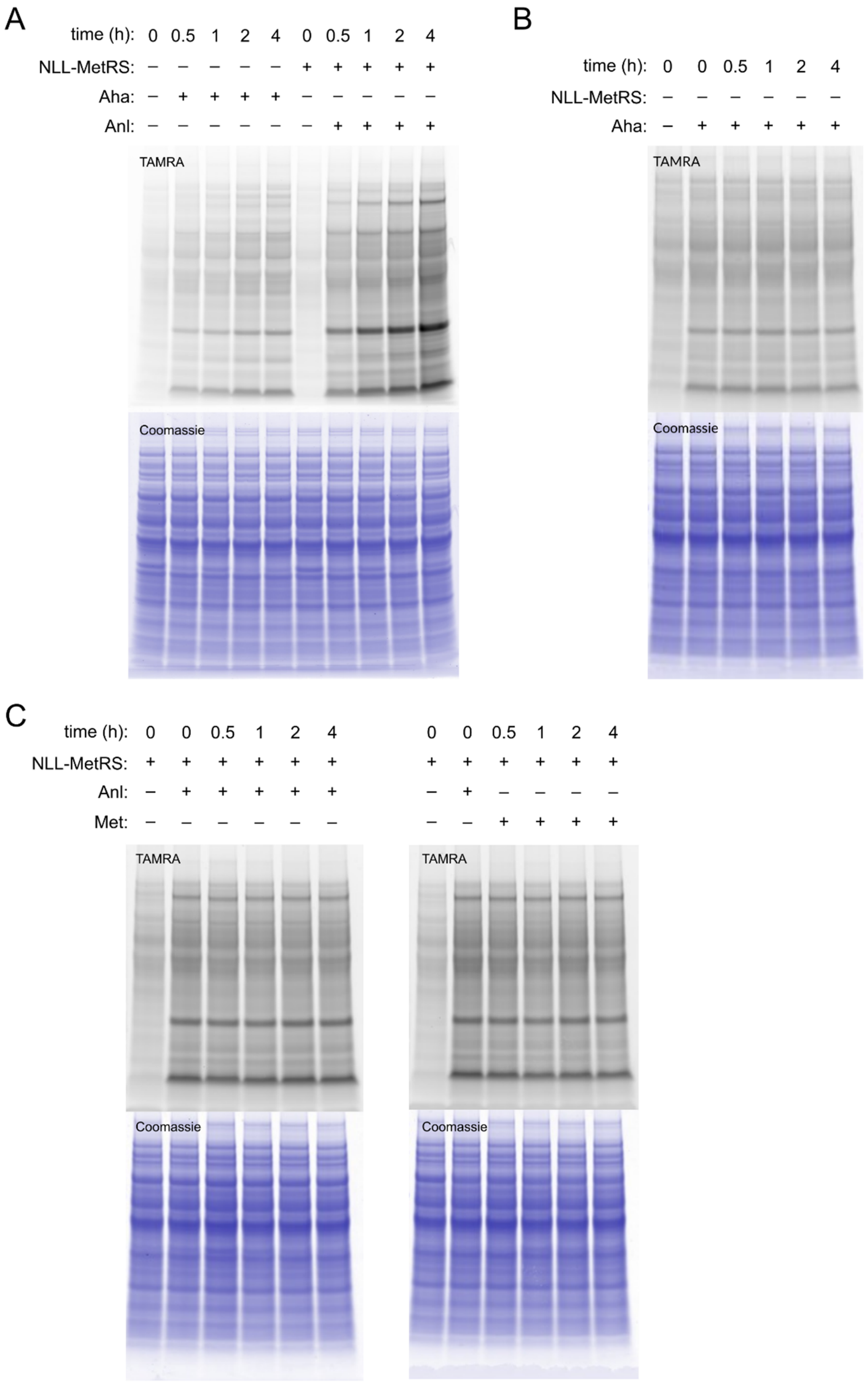


**Figure S11. In-gel fluorescence analysis of Aha and Anl incorporation in stationary phase cells.** (A) In-gel fluorescence detection to determine sufficient labeling periods with Aha or Anl. Cells from an

overnight culture of MG1655 (NLL-MetRS$^{-}$) or MG1655 harboring PrrnBp1-dRBS-GFP-NLL-MetRS (NLL-MetRS$^{+}$) in M9 glycerol medium were diluted 1:200 in fresh medium, grown 24 h, and treated with 1 mM Aha or 1 mM Anl. Aliquots were collected at the indicated time points following initiation of treatment with Aha or Anl, and cells were collected and lysed. Lysates were used in click conjugation to TAMRA-alkyne, and proteins were separated by mass via SDS-PAGE for in-gel fluorescence detection. (B) Repeated in-gel fluorescence detection of Aha labeling in stationary phase for an expanded labeling duration suggests Aha performs unreliably in stationary phase cells. MG1655 cells from an overnight culture in M9 glycerol medium were diluted 1:200 in fresh medium, grown 24 h, and treated with 1 mM Aha for 4 h. Aliquots were then collected at the indicated time points following the 4 h Aha treatment, and cells were collected and lysed. Lysates were used in click conjugation to TAMRA-alkyne, and proteins were separated by mass via SDS-PAGE for in-gel fluorescence detection. (C) Extended results from Fig. 5A including cells that were not subjected to an Anl washout in spent medium supplemented with 1 mM Met. Cells treated with Anl for 4 h were collected at the indicated time point without (left) and with (right, as presented in Fig. 5A) an intervening medium exchange to spent medium supplemented with 1 mM Met.

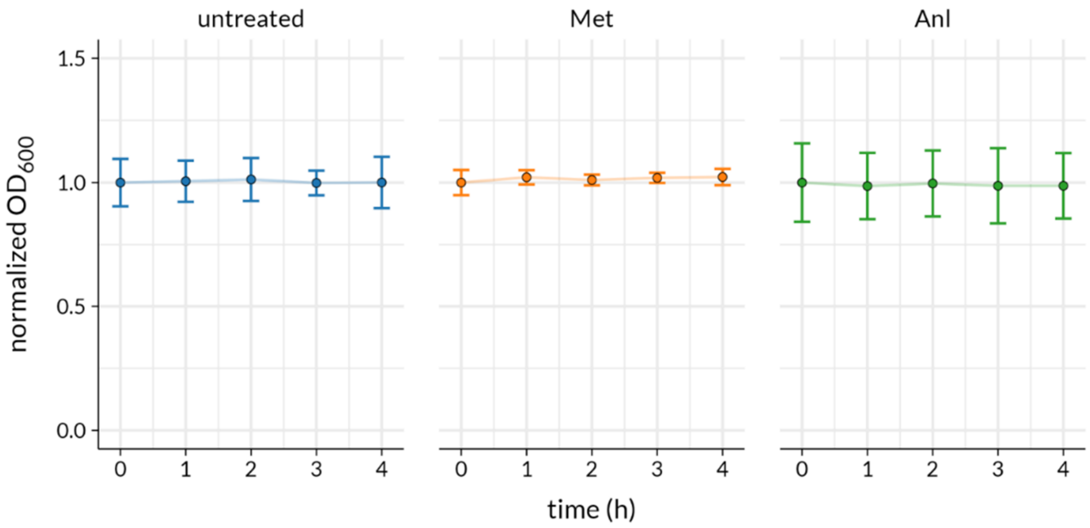


**Figure S12. Culture density measurements of stationary phase cells treated with Met or Anl.** Cells from an overnight culture in M9 glycerol medium were diluted 1:200 in fresh medium, grown 24 h, and treated with 1 mM Met or 1 mM Anl. Cells were removed from cultures at the indicated timepoints for optical density measurements. Measurements were made in triplicate and normalized to the mean optical density at the initial timepoint for each series. Error bars indicate 95% confidence intervals calculated using the *t*-distribution.

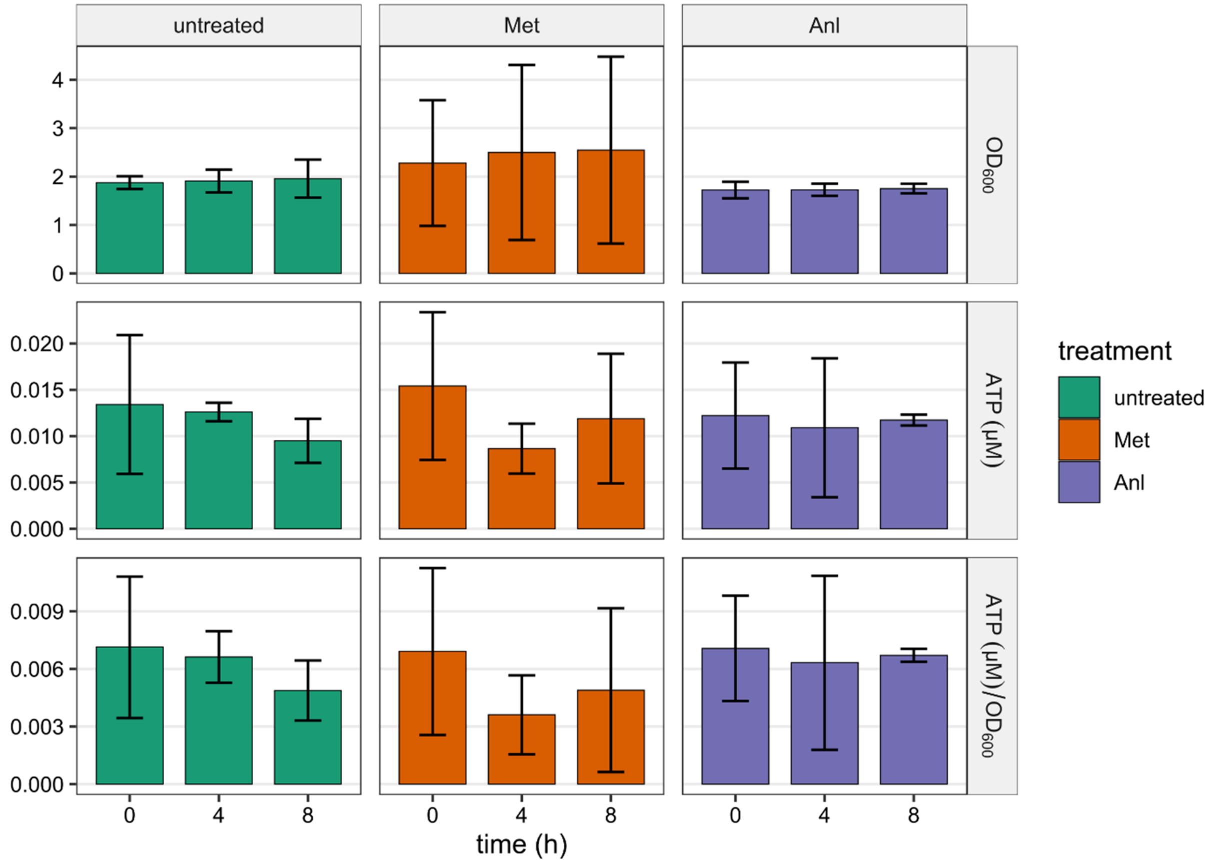


**Figure S13. ATP and culture density measurements in stationary phase cells following the medium exchange.** Cells from an overnight culture in M9 glycerol medium were diluted 1:200 in fresh medium, grown 24 h, treated with 1 mM Met or 1 mM Anl for 4 h, and resuspended in spent medium supplemented with 1 mM Met. Cells were then removed from cultures at the indicated time points during the observation window for optical density measurements and luminescence-based quantification of ATP. Bars report the mean value (n = 4), and error bars report 95% confidence intervals calculated using the *t*-distribution. When fitting a full factorial linear mixed-effects model to ATP normalized to $OD_{600}$ with time and condition (untreated, Met, or Aha) covariates as fixed effects and replicate identifier as a random effect, we observe no statistically significant effects ($\alpha = 0.05$).

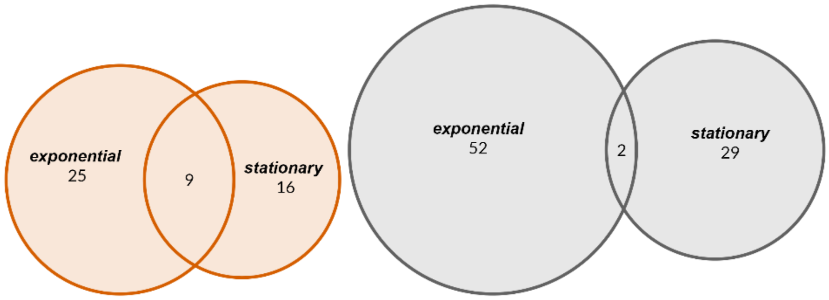


**Figure S14. Comparison of the identities of substrates identified in the two proteomic analyses across exponential and stationary phases.** Venn diagrams of substrate candidates with pronounced instability (estimated half-life corresponding to fold change decrease > 2 over the course of each experiment; FDR-adjusted *p*-value < 0.05) in each substrate annotation class. Orange shading represents annotated substrates, and grey shading indicates a lack of evidence of instability in the literature.

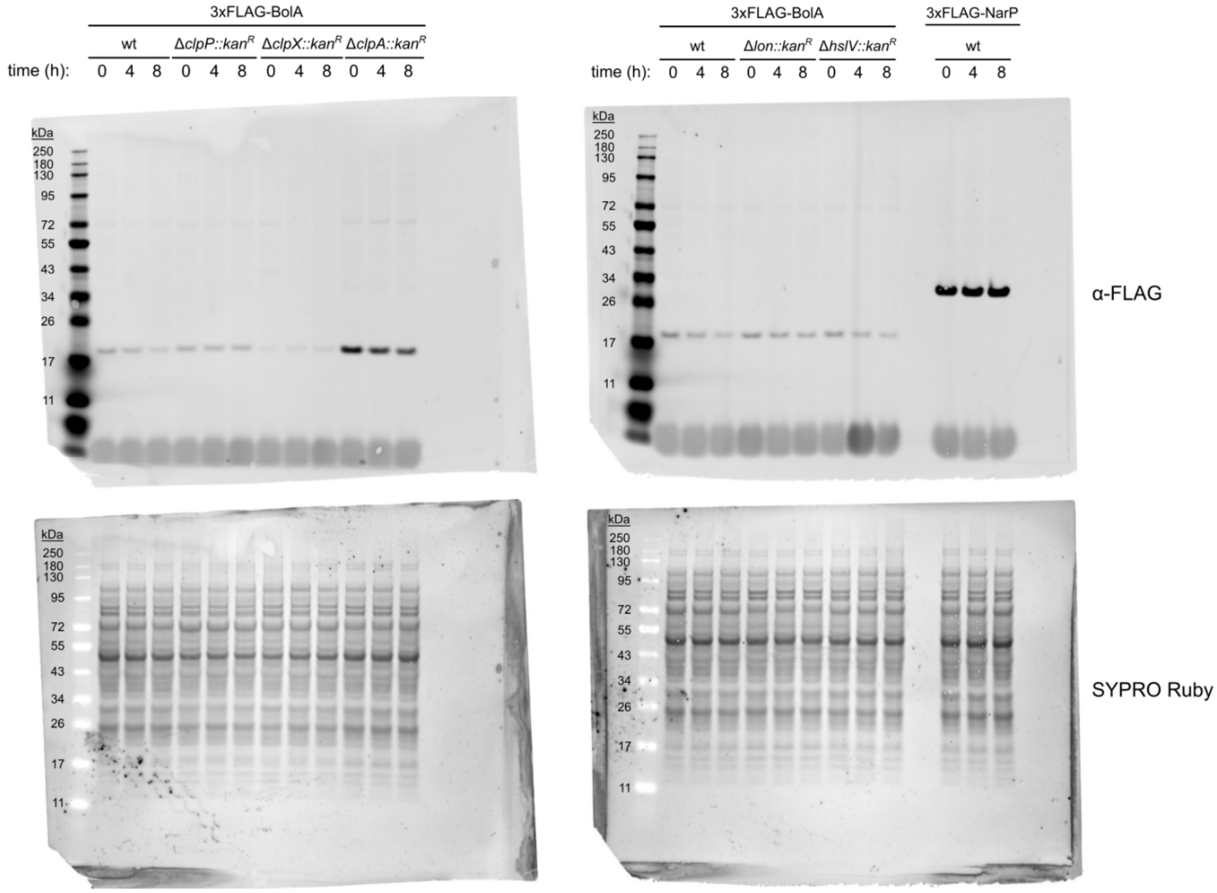


**Figure S15. Protease mapping of 3xFLAG-BolA in stationary phase cells.** Immunoblotting stability analysis of 3xFLAG-BolA expressed from a plasmid (pBbA2c) in stationary phase cells. 3xFLAG-BolA was cloned into a medium-copy pBbA2c expression vector for expression driven by the PLtetO-1 promoter upon addition of anhydrotetracycline (aTc) (4). Cells from an overnight culture in M9 glycerol medium supplemented with 25 µg/mL chloramphenicol for plasmid maintenance grown at 37 °C were diluted 1:200 in fresh M9 glycerol medium and incubated at 37 °C for 24 h. Cells were then treated with 200 nM aTc for 4 h to induce expression of the fusion protein followed by 200 µg/mL spectinomycin to inhibit protein synthesis. At the indicated time point, cells were collected at 18000 RCF for 1 min, lysed in 0.5% SDS in PBS, heat-treated at 95 °C for 15 min, and stored at -20 °C for immunoblotting. Deletion of *clpP*, *clpA*, and *lon* elicited stabilizing effects relative to the MG1655 wildtype background as seen in our exponential phase screening protocol for this same fusion (*SI Appendix*, Figs. S16 and S25). However, the low expression levels attained in stationary phase may preclude screening of other stationary phase candidate substrates, and the systematic decreases or increases in expression seen in the *clpX* and *clpA* backgrounds, respectively, suggest phenotypic effects of these deletions in stationary phase may confound protease assignment. The 3xFLAG-NarP samples were produced with the same protocol and did not exhibit instability in this screen.

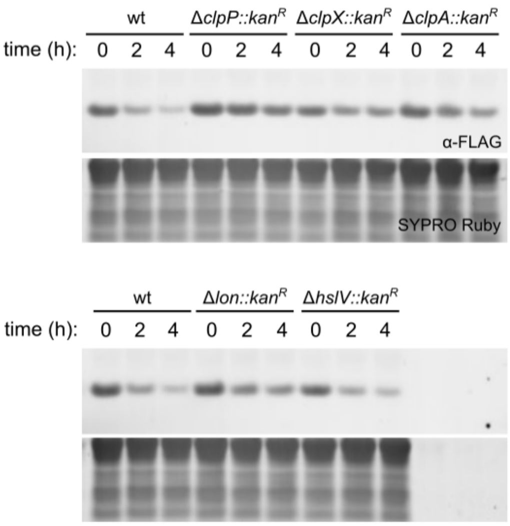


**Figure S16. Protease mapping of 3xFLAG-BolA in exponential phase cells.** Immunoblotting stability analysis of 3xFLAG-BolA expressed from a plasmid (pBAD33) in exponential phase cells resulted in variable stabilization across single deletion mutants, suggesting multiple proteases contributed to the observed instability. Full membranes with molecular weight annotations are reported in *SI Appendix*, Fig. S25.

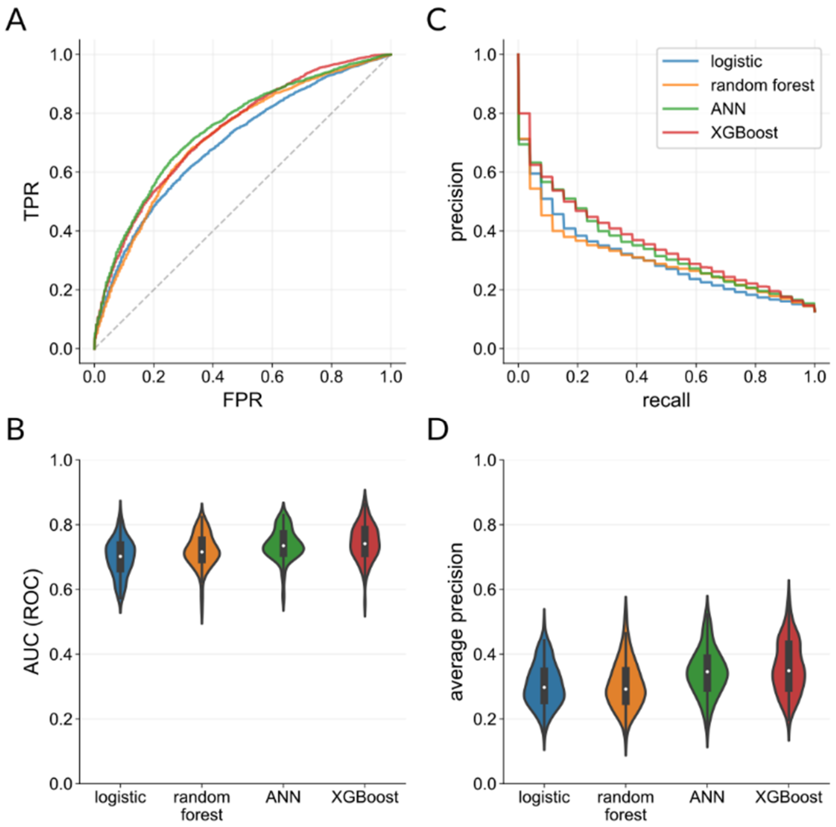


**Figure S17. An XGBoost classifier improves precision 2.8-fold over baseline.** (A) Averaged ROC curves for models across all test sets from repeated nested cross validation. (B) Violin plots of AUC calculations for ROC curves in each test set from repeated nested cross validation. (C) Averaged precision-recall curves for models across all test sets from repeated nested cross validation. (D) Violin plots of average precision in each test set from repeated nested cross validation.

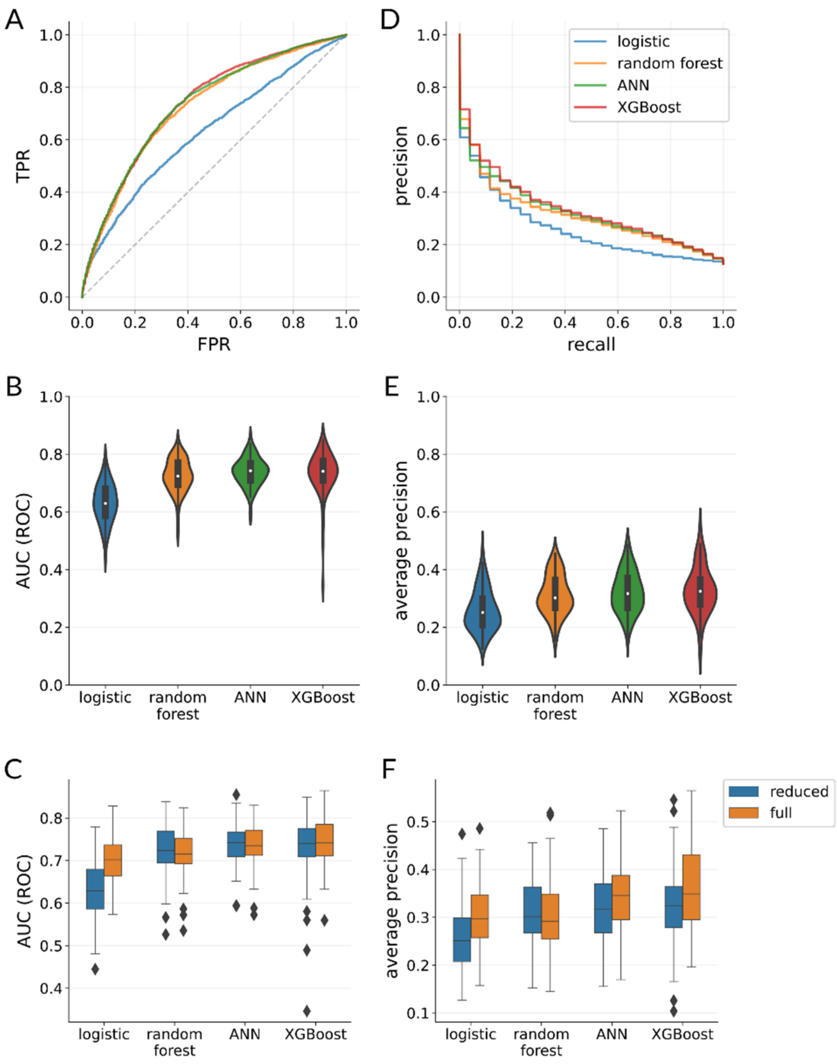


**Figure S18. Model training and cross validation with a reduced set of features.** The predictive features were reduced to a set containing physicochemical properties and predicted properties that are widely available or readily produced given primary amino acid sequences (Table S1). (A) Averaged ROC curves for models across all test sets from repeated nested cross validation. (B) Violin plots of AUC calculations for ROC curves in each test set from repeated nested cross validation. (C) Boxplots of AUC calculations for ROC curves in each test set from repeated nested cross validation stratified by feature set used for training. (D) Averaged precision-recall curves for models across all test sets from repeated nested cross validation. (E) Violin plots of average precision in each test set from repeated nested cross validation. (F) Boxplots of average precision in each test set from repeated nested cross validation stratified by feature set used for training.

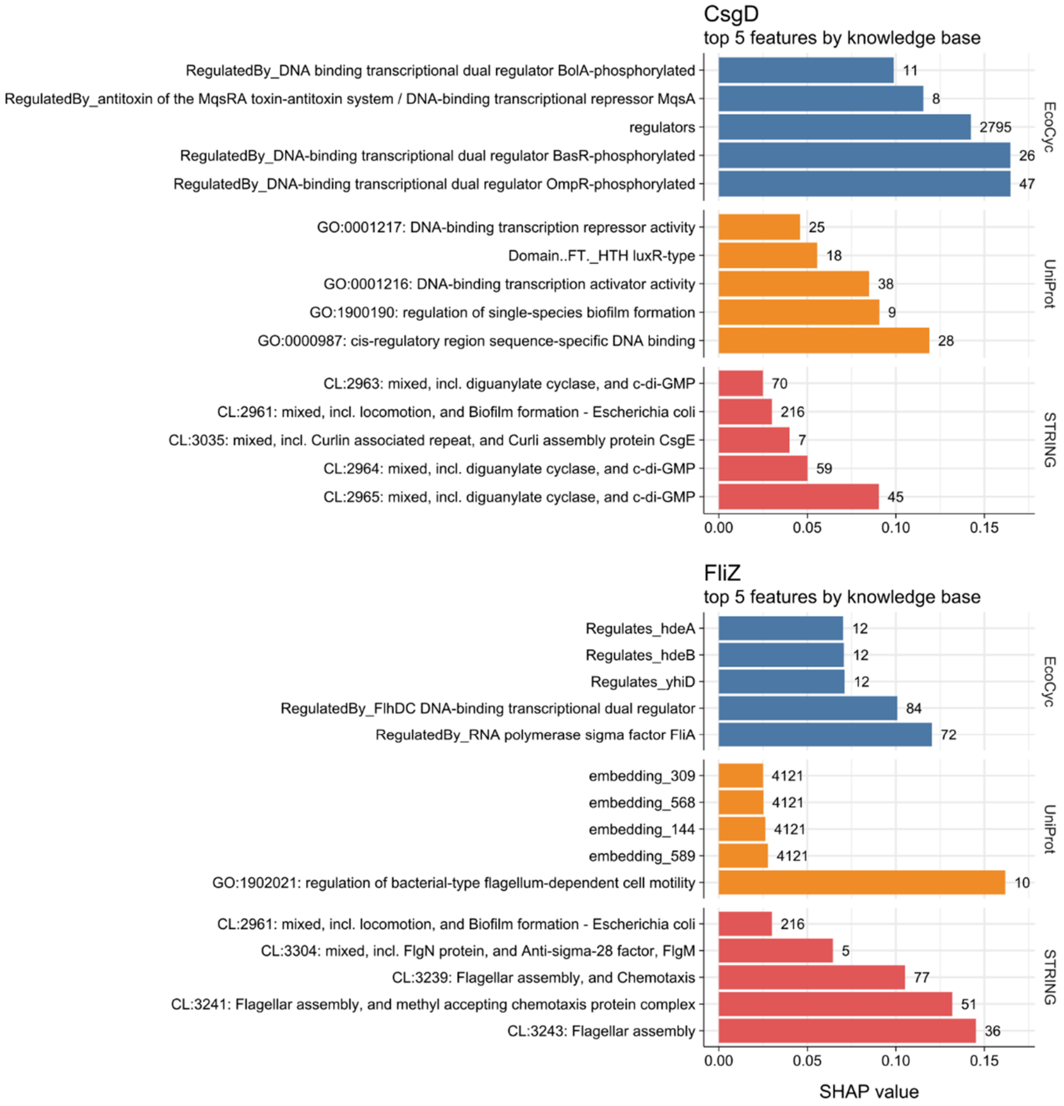


**Figure S19. Top predictive features across data sources for CsgD and FliZ.** The top 5 features across annotation databases for predicted substrates CsgD and FliZ based on SHAP value. The number of non-zero elements for each feature is offset from the reported SHAP value.

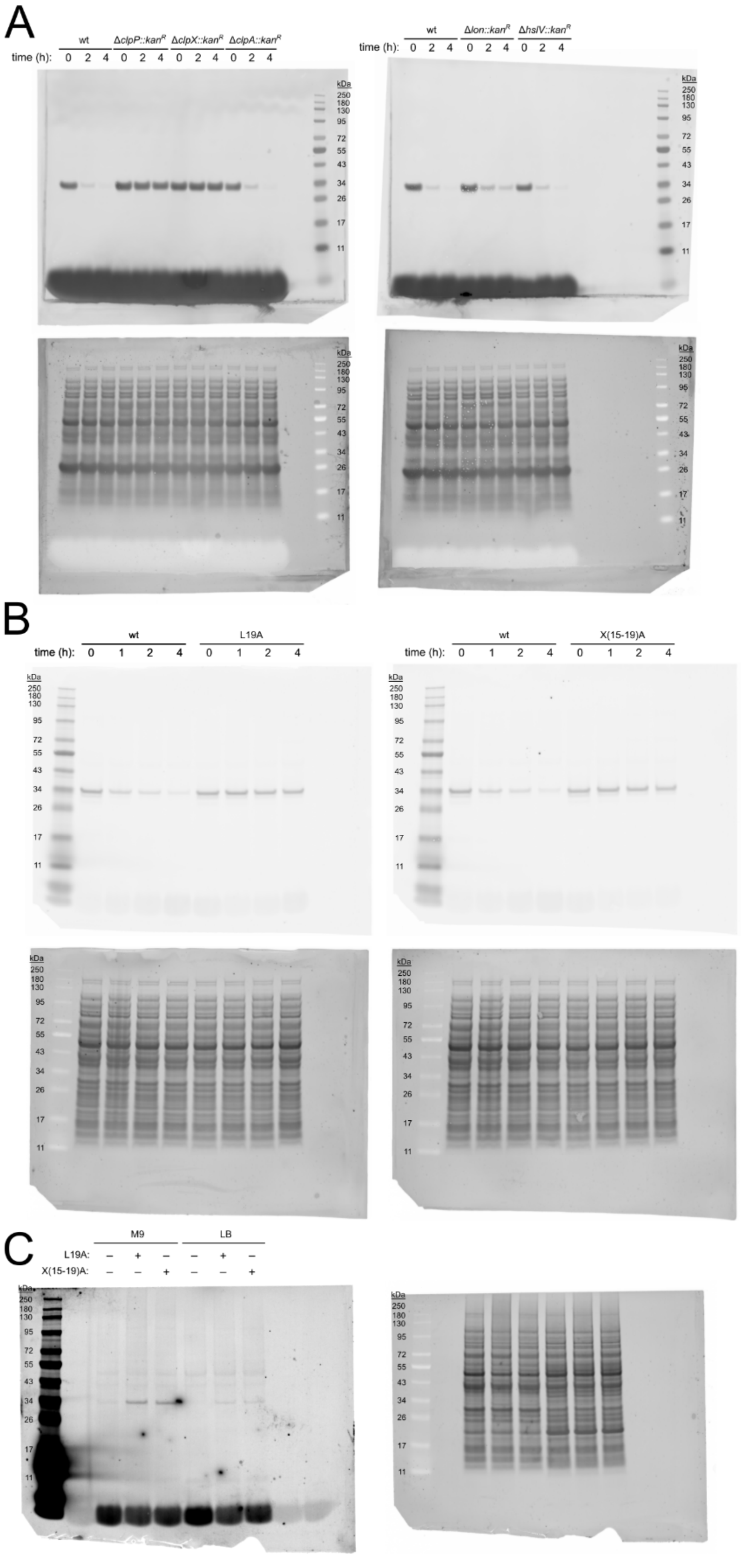


**Figure S20. Full membranes for immunoblotting featured in Figs. 3C-E.** (A) Membranes for immunoblotting stability analysis of PdeH-FLAG in wild type or protease deletion backgrounds. (B) Membranes for immunoblotting stability analysis of wild type or mutant PdeH-3xFLAG expressed from the chromosome. (C) Membranes for immunoblotting of wild type or mutant PdeH-3xFLAG expressed from the chromosome in stationary phase.

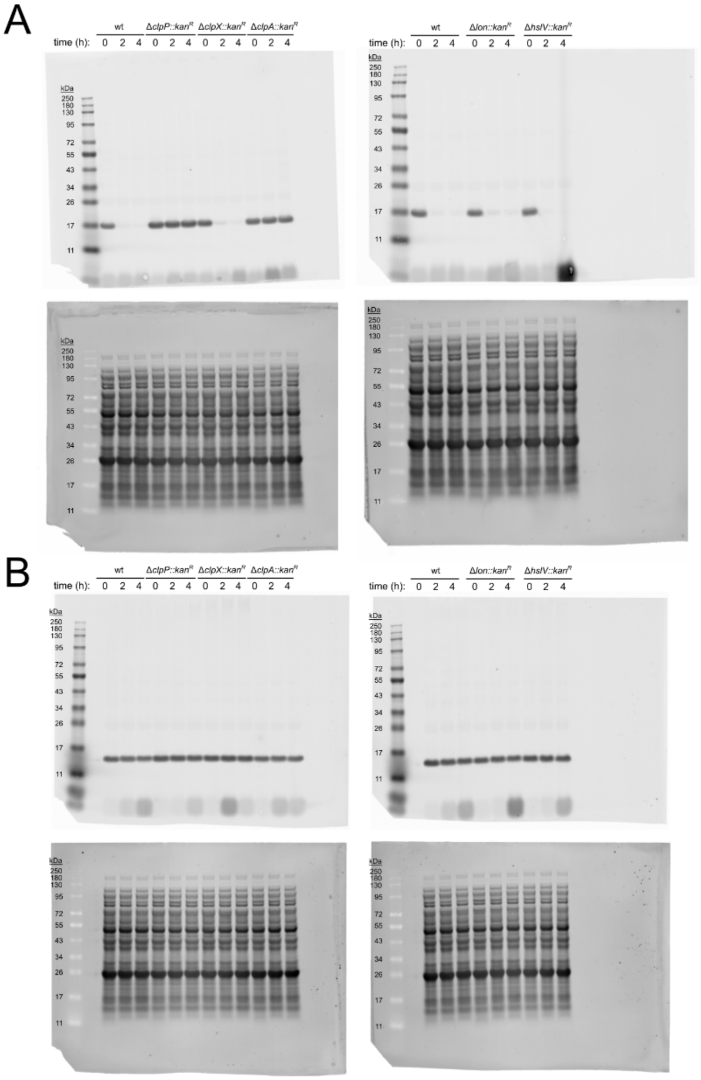


**Figure S21. Full membranes for immunoblotting featured in Fig. 4C.** (A) Membranes for immunoblotting stability analysis of ClpS-3xFLAG in wild type or protease deletion backgrounds. (B) Membranes for immunoblotting stability analysis of ΔNTE-ClpS-3xFLAG in wild type or protease deletion backgrounds.

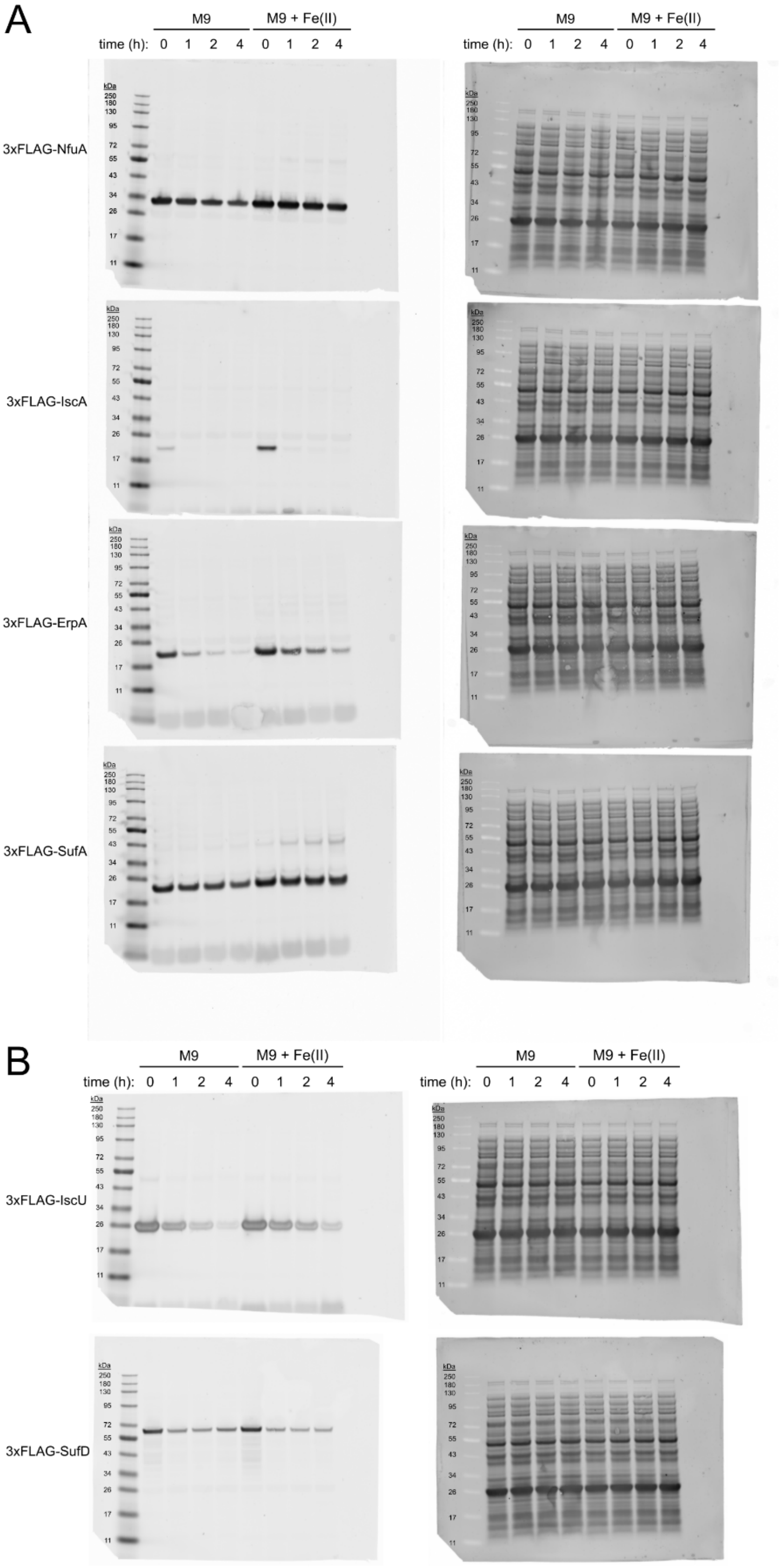


**Figure S22. Full membranes for immunoblotting featured in Fig. 4E.** (A) Membranes for the immunoblotting stability analysis of ATCs bearing an N-terminal 3xFLAG epitope without or with 20 µM $FeSO_4$ added to the growth medium. (B) Membranes for the immunoblotting stability analysis of 3xFLAG-IscU and 3xFLAG-SufD without or with 20 µM $FeSO_4$ added to the growth medium.

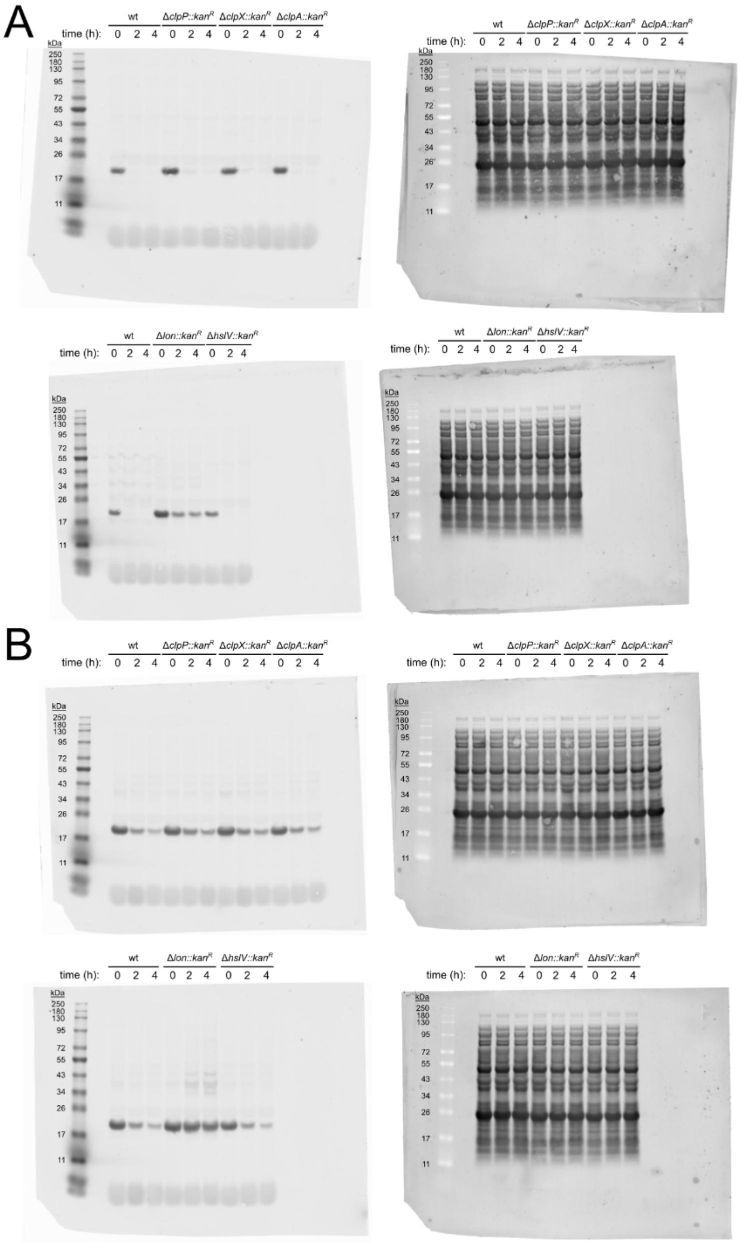


**Figure S23. Full membranes for immunoblotting featured in Fig. 4F.** (A) Membranes for the immunoblotting stability analysis of 3xFLAG-IscA in wild type or protease deletion backgrounds. (B) Membranes for the immunoblotting stability analysis of 3xFLAG-ErpA in wild type or protease deletion backgrounds.

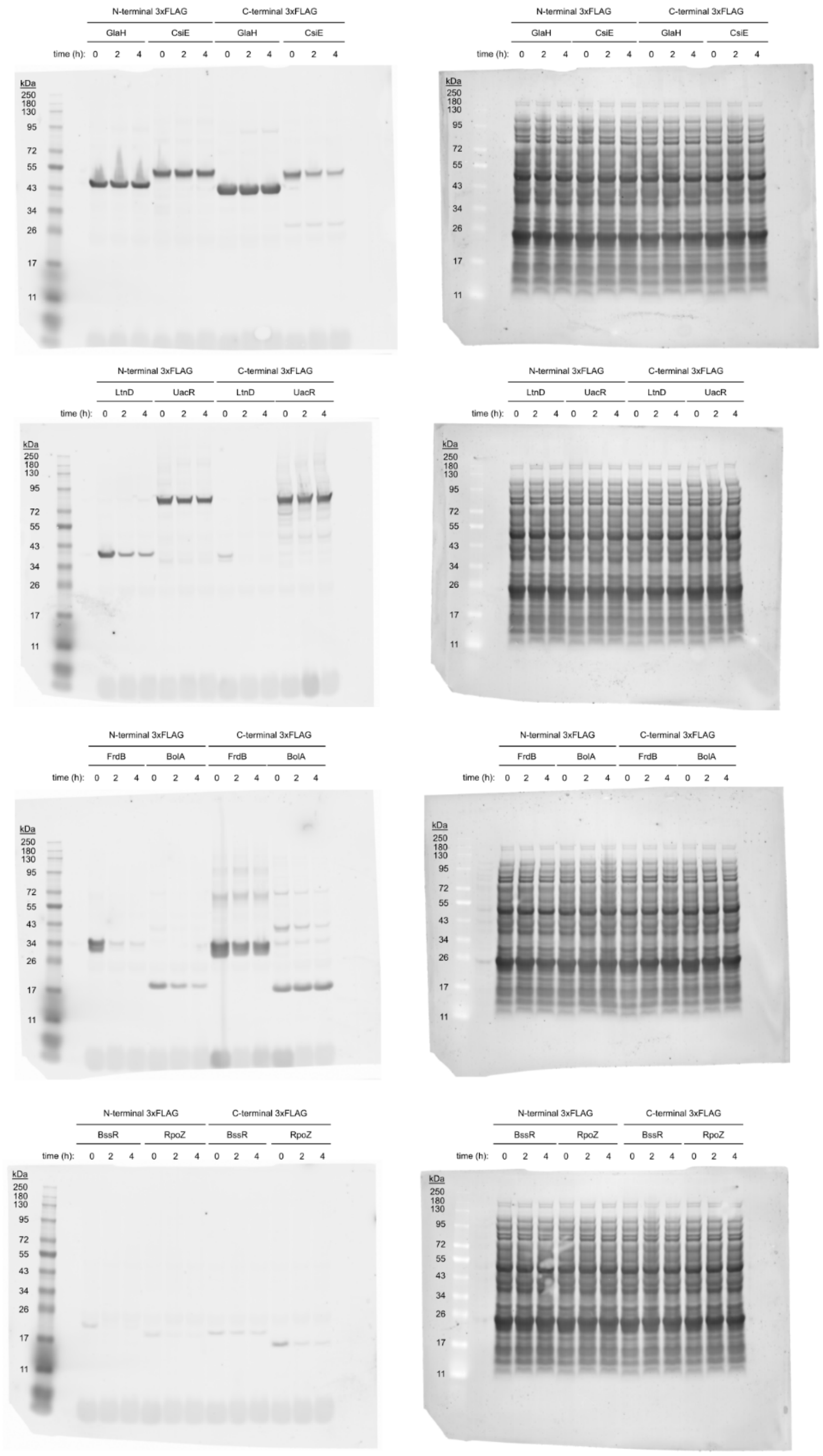


**Figure S24. Full membranes for immunoblotting featured in Fig. 5F.** Membranes for immunoblotting stability analysis of candidate substrates identified in the stationary phase degradation profiling.

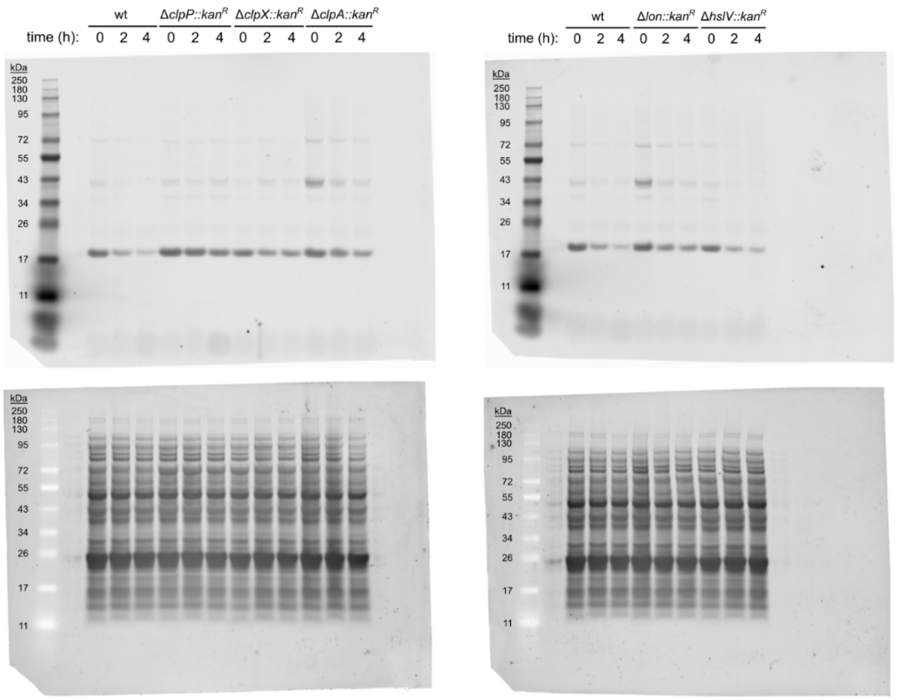


**Figure S25. Full membranes for immunoblotting featured in Figs. 5G and S16.** Membranes for immunoblotting stability analysis of 3xFLAG-BolA in wild type or protease deletion backgrounds.

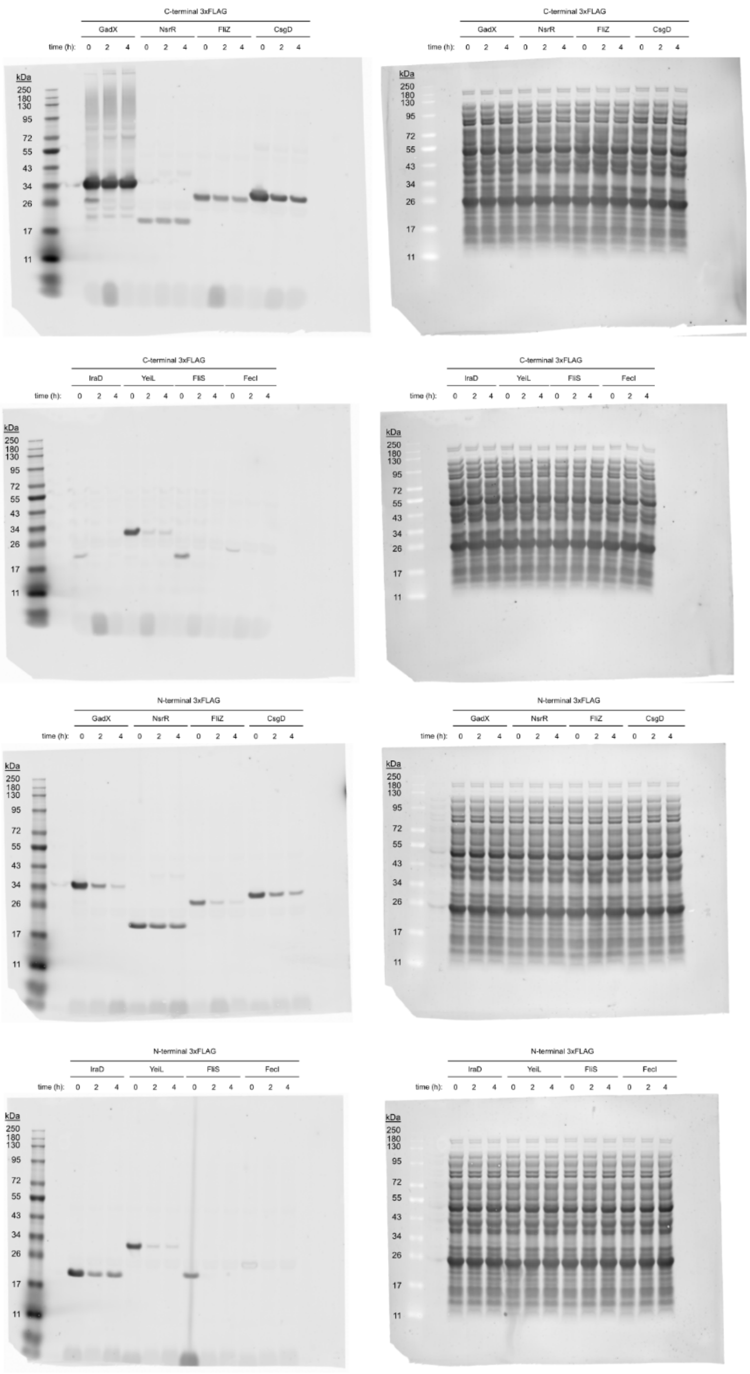


**Figure S26. Full membranes for immunoblotting featured in Fig. 6C.** Membranes for immunoblotting stability analysis of candidate substrates identified through the XGBoost model.

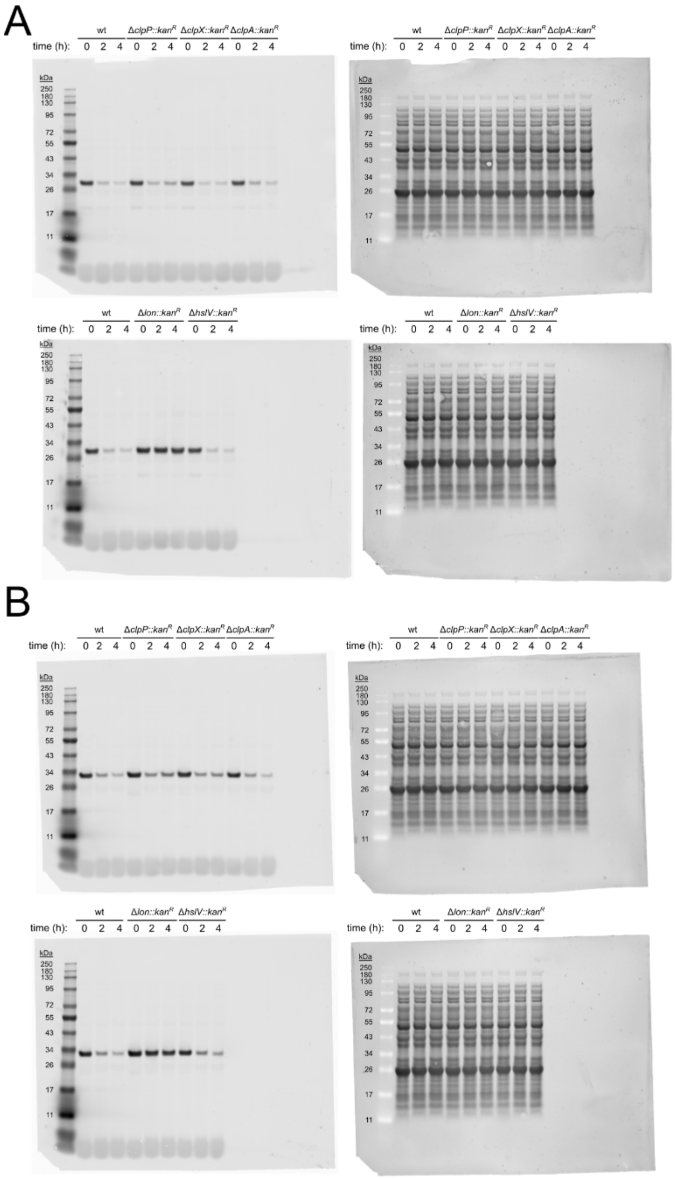


**Figure S27. Full membranes for immunoblotting featured in Fig. 7A.** Membranes for immunoblotting stability analysis of (A) 3xFLAG-FliZ and (B) 3xFLAG-CsgD in wild type or protease deletion backgrounds.

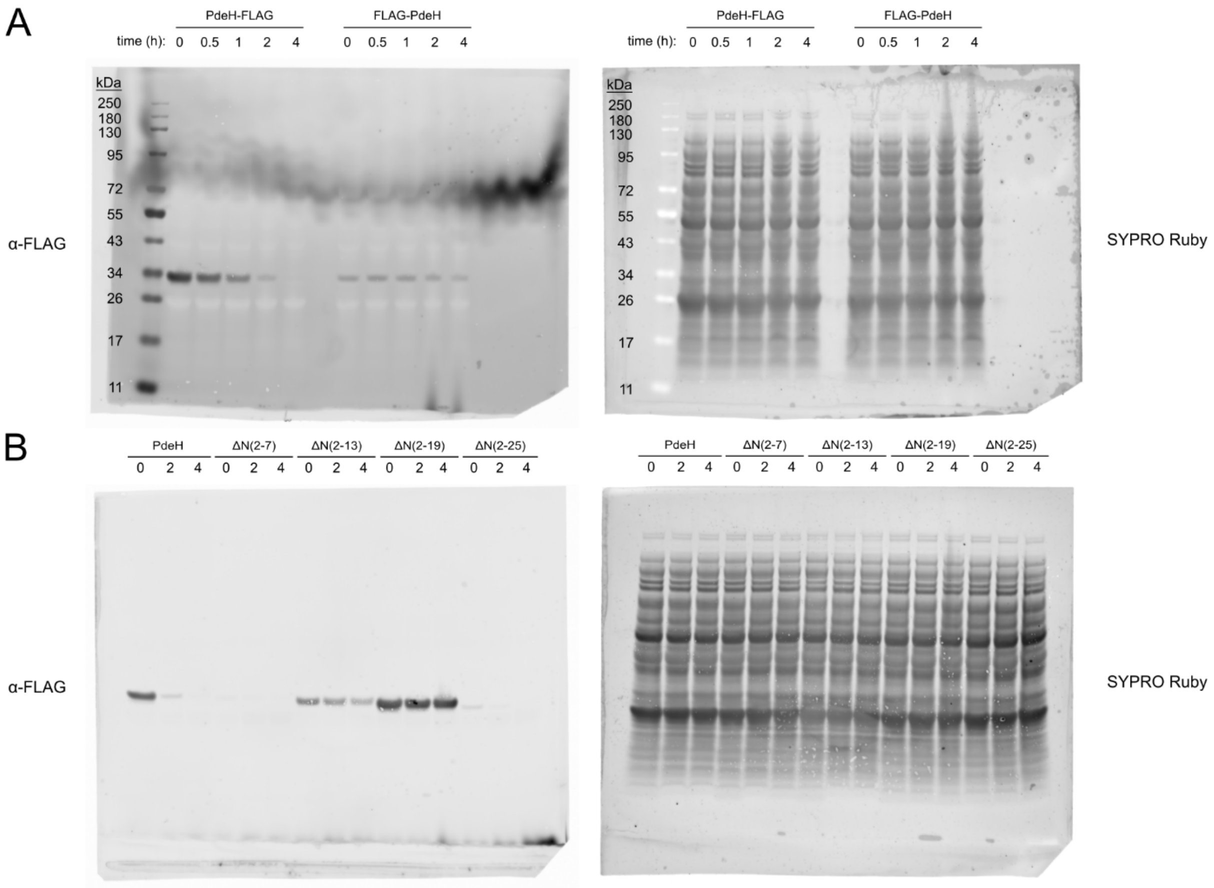


**Figure S28. Full membranes for immunoblotting featured in Fig. S6.** (A) Membranes for immunoblotting stability of PdeH-FLAG and FLAG-PdeH. (B) Membranes for immunoblotting of N-terminal truncations of PdeH-FLAG.

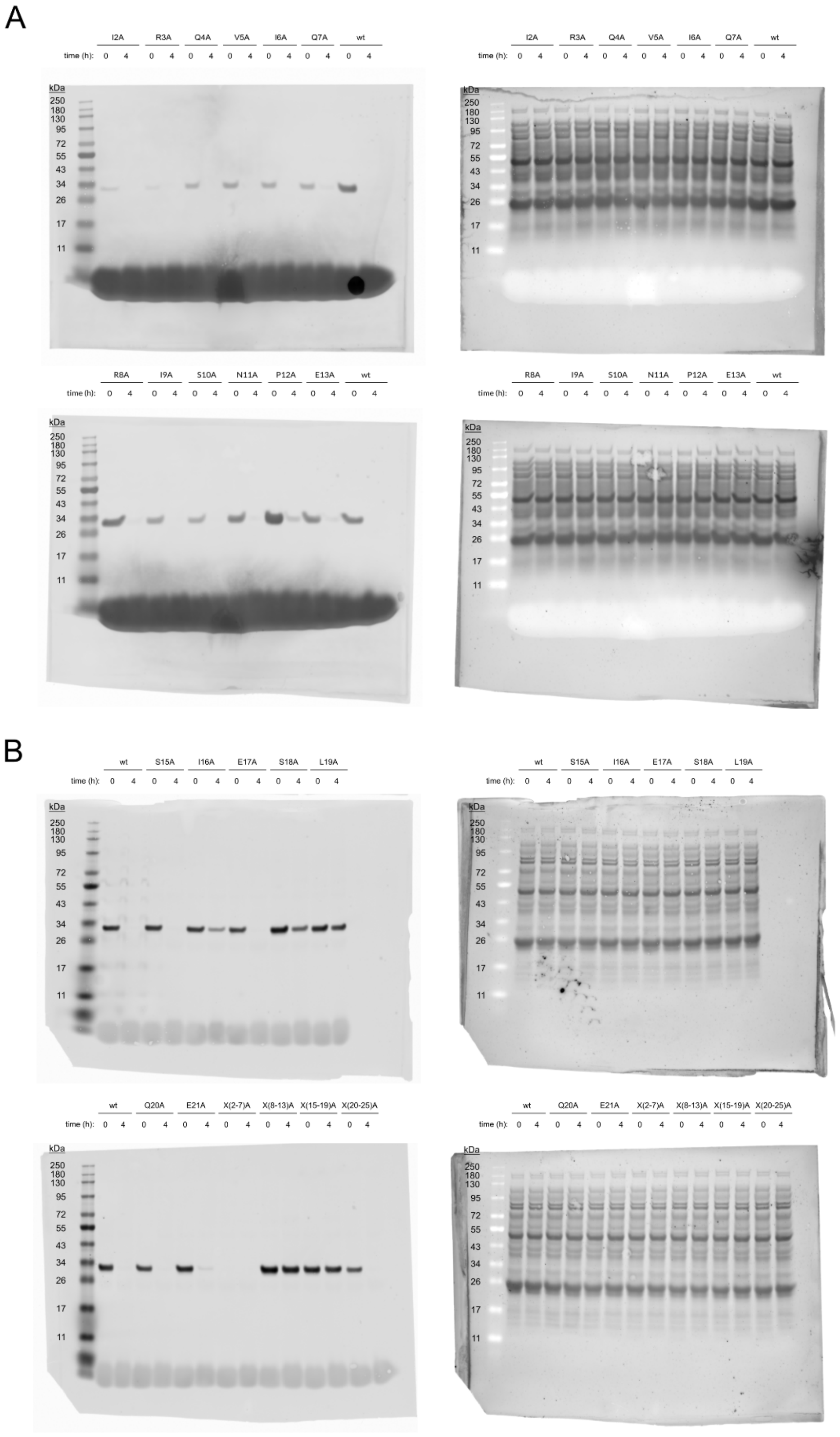


**Figure S29. Full membranes for immunoblotting featured in Fig. S7.** (A) Full membranes for immunoblotting of alanine scanning mutagenesis of residues 2-13 of the PdeH N terminus. (B) Full

membranes for immunoblotting of alanine scanning mutagenesis of residues 15-21 and alanine stretch mutagenesis of residues 2-7, 8-13, 15-19, and 20-25 of the PdeH N terminus.

**Table S1. Features used in machine learning classification of protein stability.** An asterisk serves as a placeholder for each distinct feature name associated with the feature set.

| Feature set | Source | Description | Count | Reduced set |
|---|---|---|---|---|
| Gene.Ontology.IDs_* | UniProt | Annotation of the protein with the indicated Gene Ontology term | 2056 | No |
| Regulates_* | EcoCyc | Annotation of regulation of the indicated gene by the protein | 1100 | No |
| Catalytic.activity_* | UniProt | Annotation of the protein participating in a chemical reaction with a species described by the indicated Chemical Entities of Biological Interest (ChEBI) ID | 1054 | No |
| CL:* | STRING | Annotation of the protein membership in the indicated pre-computed hierarchical STRING database cluster | 1031 | No |
| embedding_* | UniProt | Per-protein ProtT5 embedding value for the indicated embedding number | 1024 | Yes |
| Protein.families_* | UniProt | Annotation of the protein as a member of the indicated protein family | 939 | No |
| EC.number_* | UniProt | Classification of the protein under the indicated Enzyme Commission (EC) number | 759 | No |
| SubstrateInteraction_* | EcoCyc | Annotation of an interaction between the protein and the indicated molecular species | 366 | No |
| Keyword.ID | UniProt | Annotation of the protein with the indicated UniProt Keyword | 327 | No |
| RegulatedBy_* | EcoCyc | Annotation of regulation of the gene encoding the protein by the indicated molecular species | 297 | No |
| Domain..FT._* | UniProt | Annotation of the indicated protein domain in the protein | 210 | No |
| Binding.site_* | UniProt | Annotation of a binding site at protein residues for the indicated chemical entity | 196 | No |
| Rhea.ID_* | UniProt | Annotation of the protein with the indicated Rhea reaction identifier | 149 | No |
| Region_* | UniProt | Annotation of a region of interest excluded by other UniProt 'Family and Domains' classifications | 99 | No |
| Cofactor_* | UniProt | Annotation of an interaction between the protein and a cofactor described by the indicated ChEBI ID | 45 | No |
| Feature_* | UniProt | Annotation of the protein with a term under the UniProt sequence annotation feature types | 28 | No |
| Subcellular.location..CC._* | UniProt | Annotation of subcellular localization of the protein for the indicated location | 25 | No |

| | | | | |
|---|---|---|---|---|
| cterm_percent_* | Biopython | Percent composition of the 6 C-terminal residues of the protein for the indicated amino acid | 20 | Yes |
| nterm_percent_* | Biopython | Percent composition of the 12 N-terminal residues of the protein for the indicated amino acid | 20 | Yes |
| percent_* | Biopython | Percent composition of the protein by amino acid for the indicated amino acid | 20 | Yes |
| Active.site | UniProt | Annotation of an active site | 1 | No |
| Coiled.coil | UniProt | Annotation of a coiled coil in the protein | 1 | No |
| Compositional.bias | UniProt | Annotation of a region of compositional bias in the protein | 1 | No |
| Cross.link | UniProt | Annotation of residues participating in covalent linkage(s) between proteins | 1 | No |
| Disulfide.bond | UniProt | Annotation of cysteines participating in disulfide bonds in the protein | 1 | No |
| DNA.binding | UniProt | Annotation of a DNA-binding domain in the protein | 1 | No |
| Initiator.methionine | UniProt | Annotation of cleavage of the initiator methionine for the protein | 1 | No |
| Interacts.with | UniProt | Annotation of any protein-protein interaction | 1 | No |
| Length | UniProt | Length of the protein in residues | 1 | Yes |
| Lipidation | UniProt | Annotation of a lipidation event for the protein | 1 | No |
| Mass | UniProt | Mass of the protein in daltons | 1 | Yes |
| Modified.residue | UniProt | Annotation of a modification at a residue of the protein excluding lipids, glycans, and protein cross-links | 1 | No |
| Post.translational.modification | UniProt | Annotation of a covalent processing event resulting from proteolytic cleavage or addition of a modifying group | 1 | No |
| Propeptide | UniProt | Annotation of a propeptide cleaved during maturation or activation of the protein | 1 | No |
| Signal.peptide | UniProt | Annotation of a signal peptide targeting the protein toward secretion of the periplasm | 1 | No |
| Transmembrane | UniProt | Annotation of a membrane-spanning region of the protein | 1 | No |
| Zinc.finger | UniProt | Annotation of a zinc finger domain in the protein | 1 | No |
| Centisome.Position | EcoCyc | Position of the gene encoding the protein on the *E. coli* chromosome | 1 | No |
| regulatees | EcoCyc | Number of genes directly regulated by the indicated protein | 1 | No |
| regulators | EcoCyc | Number of direct regulators of the gene encoding the protein | 1 | No |

| | | | | |
|---|---|---|---|---|
| aromaticity | Biopython | Aromaticity value of the protein calculated with all amino acids | 1 | Yes |
| charge | Biopython | Charge calculated with all amino acids of the protein at pH 7.8 | 1 | Yes |
| gravy | Biopython | Sum of hydropathy values of amino acids in the protein divided by protein length (GRAVY) | 1 | Yes |
| isoelectric_point | Biopython | Isoelectric point calculated with all amino acids of the protein | 1 | Yes |
| AF_c_score | AlphaFold2 | Average pLDDT score for the 20 C-terminal residues of the protein | 1 | Yes |
| AF_mean_score | AlphaFold2 | Average pLDDT score for all residues of the protein | 1 | Yes |
| AF_n_score | AlphaFold2 | Average pLDDT score for the 20 N-terminal residues of the protein | 1 | Yes |
| c_degron | (17) | Presence of a C-terminal degron described in the literature | 1 | No |
| n_degron | (17) | Presence of an N-terminal degron described in the literature | 1 | No |

**Table S2. Hyperparameters used in grid searching during nested cross validation for each model class and for each set of features (full or reduced) used in training**

| Model | Parameter | Values (Full) | Values (Reduced) |
|---|---|---|---|
| Logistic regression | C | 1e3, 1e4, 1e5 | 1e3, 1e4, 1e5 |
| Random forest | max_depth | 5, 10, 15, None | 5, 10, 15, None |
| | min_samples_leaf | 50, 100 | 50, 100 |
| | min_samples_split | 2, 5, 10 | 2, 5, 10 |
| | n_estimators | 50, 100 | 50, 100 |
| Artificial neural network | Weight decay | 1e-2, 1e-3 | 1e-2, 1e-3 |
| | Dropout | 0.2, 0.3, 0.4, 0.5 | 0.2, 0.3, 0.4, 0.5 |
| | Hidden units | 20, 50, 100, 200 | 20, 50, 100, 200 |
| XGBoost | learning_rate | 1e-1, 1e-2 | 1e-1, 1e-2 |
| | reg_lambda | 1e-2, 1e-3, 1e-4 | 1e-5, 1e-6, 1e-7 |
| | reg_alpha | 1e-3, 5e-4, 1e-4 | 1e-6, 5e-6, 1e-7 |
| | n_estimators | 25, 10 | 25, 10 |

**Table S3. Strains used in this study.**

| Strain | Description | Reference |
|---|---|---|
| MG1655 | *Escherichia coli* K-12 derivative | (18) |
| MG1655 Δ*clpP::kan*$^R$ | ClpP knockout | This work |
| MG1655 Δ*clpX::kan*$^R$ | ClpX knockout | This work |
| MG1655 Δ*clpA::kan*$^R$ | ClpA knockout | This work |
| MG1655 Δ*lon::kan*$^R$ | Lon knockout | This work |
| MG1655 Δ*hslV::kan*$^R$ | HslV knockout | This work |
| MG1655 Δ*csgB::kan*$^R$ | CsgB knockout | This work |
| MG1655 Δ*pdeH::kan*$^R$ | PdeH knockout | This work |
| MG1655 *pdeH-3xFLAG* | C-terminal 3xFLAG insertion at *pdeH* locus | This work |
| MG1655 *pdeH(L19A)-3xFLAG* | N-terminal L19A mutation and C-terminal 3xFLAG insertion at *pdeH* locus | This work |
| MG1655 *pdeH(X(15-19)A)-3xFLAG* | N-terminal alanine stretch spanning residues 15-19 and C-terminal 3xFLAG insertion at *pdeH* locus | This work |
| MG1655 *pdeH(L19A)* | N-terminal L19A mutation at *pdeH* locus | This work |
| MG1655 *pdeH(E48A)* | E48A mutation at *pdeH* locus | This work |
| MG1655 *pdeH(X(15-19)A)* | N-terminal alanine stretch spanning residues 15-19 at *pdeH* locus | This work |

**Table S4. Plasmids used in this study.**

| Plasmid | Description | Reference |
|---|---|---|
| pKD46 | Recombinase expression for chromosomal deletion | (1) |
| pKD13 | Template plasmid for kanamycin resistance cassette insertion | (1) |
| pBAD33 | Empty vector for arabinose-inducible expression | (2) |
| pBAD33-FLAG | pBAD33 bearing FLAG epitope sequence | This work |
| pBAD33-pdeH-FLAG | Arabinose-inducible expression of PdeH-FLAG | This work |
| pBAD33-FLAG-pdeH | Arabinose-inducible expression of FLAG-pdeH | This work |
| pBAD33-ΔN(2-7)-pdeH-FLAG | Arabinose-inducible expression of N-terminal truncation of PdeH-FLAG | This work |
| pBAD33-ΔN(2-13)-pdeH-FLAG | Arabinose-inducible expression of N-terminal truncation of PdeH-FLAG | This work |
| pBAD33-ΔN(2-19)-pdeH-FLAG | Arabinose-inducible expression of N-terminal truncation of PdeH-FLAG | This work |
| pBAD33-ΔN(2-25)-pdeH-FLAG | Arabinose-inducible expression of N-terminal truncation of PdeH-FLAG | This work |
| pBAD33-pdeH(I2A)-FLAG | Arabinose-inducible expression of alanine point mutant of PdeH-FLAG | This work |
| pBAD33-pdeH(R3A)-FLAG | Arabinose-inducible expression of alanine point mutant of PdeH-FLAG | This work |
| pBAD33-pdeH(Q4A)-FLAG | Arabinose-inducible expression of alanine point mutant of PdeH-FLAG | This work |
| pBAD33-pdeH(V5A)-FLAG | Arabinose-inducible expression of alanine point mutant of PdeH-FLAG | This work |
| pBAD33-pdeH(I6A)-FLAG | Arabinose-inducible expression of alanine point mutant of PdeH-FLAG | This work |
| pBAD33-pdeH(Q7A)-FLAG | Arabinose-inducible expression of alanine point mutant of PdeH-FLAG | This work |
| pBAD33-pdeH(R8A)-FLAG | Arabinose-inducible expression of alanine point mutant of PdeH-FLAG | This work |
| pBAD33-pdeH(I9A)-FLAG | Arabinose-inducible expression of alanine point mutant of PdeH-FLAG | This work |
| pBAD33-pdeH(S10A)-FLAG | Arabinose-inducible expression of alanine point mutant of PdeH-FLAG | This work |

| | | |
|---|---|---|
| pBAD33-pdeH(N11A)-FLAG | Arabinose-inducible expression of alanine point mutant of PdeH-FLAG | This work |
| pBAD33-pdeH(P12A)-FLAG | Arabinose-inducible expression of alanine point mutant of PdeH-FLAG | This work |
| pBAD33-pdeH(E13A)-FLAG | Arabinose-inducible expression of alanine point mutant of PdeH-FLAG | This work |
| pBAD33-pdeH(S15A)-FLAG | Arabinose-inducible expression of alanine point mutant of PdeH-FLAG | This work |
| pBAD33-pdeH(I16A)-FLAG | Arabinose-inducible expression of alanine point mutant of PdeH-FLAG | This work |
| pBAD33-pdeH(E17A)-FLAG | Arabinose-inducible expression of alanine point mutant of PdeH-FLAG | This work |
| pBAD33-pdeH(S18A)-FLAG | Arabinose-inducible expression of alanine point mutant of PdeH-FLAG | This work |
| pBAD33-pdeH(L19A)-FLAG | Arabinose-inducible expression of alanine point mutant of PdeH-FLAG | This work |
| pBAD33-pdeH(Q20A)-FLAG | Arabinose-inducible expression of alanine point mutant of PdeH-FLAG | This work |
| pBAD33-pdeH(E21A)-FLAG | Arabinose-inducible expression of alanine point mutant of PdeH-FLAG | This work |
| pBAD33-pdeH-X(2-7)A-FLAG | Arabinose-inducible expression of alanine stretch mutant of PdeH-FLAG | This work |
| pBAD33-pdeH-X(8-13)A-FLAG | Arabinose-inducible expression of alanine stretch mutant of PdeH-FLAG | This work |
| pBAD33-pdeH-X(15-19)A-FLAG | Arabinose-inducible expression of alanine stretch mutant of PdeH-FLAG | This work |
| pBAD33-pdeH-X(20-25)A-FLAG | Arabinose-inducible expression of alanine stretch mutant of PdeH-FLAG | This work |
| pTarget | sgRNA expression vector | (5) |
| pCas | Cas9 expression vector | (5) |
| pTarget-pdeH(N20) | sgRNA expression targeting Cas9 to *pdeH* locus | This work |
| pTarget-pdeH-3xFLAG | sgRNA expression targeting Cas9 to *pdeH* locus with PdeH-3xFLAG donor DNA | This work |
| pTarget-pdeH(L19A)-3xFLAG | sgRNA expression targeting Cas9 to *pdeH* locus with PdeH(L19A)-3xFLAG donor DNA | This work |

| | | |
|---|---|---|
| pTarget-pdeH(E48A) | sgRNA expression targeting Cas9 to *pdeH* locus with PdeH(E48A) donor DNA | This work |
| pBAD33-clpS-3xFLAG | Arabinose-inducible expression of ClpS-3xFLAG | This work |
| pBAD33-3xFLAG-clpS | Arabinose-inducible expression of 3xFLAG-ClpS | This work |
| pBAD33-ΔNTE-clpS-3xFLAG | Arabinose-inducible expression of ΔNTE-ClpS-3xFLAG | This work |
| pBAD33-3xFLAG-iscA | Arabinose-inducible expression of 3xFLAG-IscA | This work |
| pBAD33-3xFLAG-erpA | Arabinose-inducible expression of 3xFLAG-ErpA | This work |
| pBAD33-3xFLAG-sufA | Arabinose-inducible expression of 3xFLAG-SufA | This work |
| pBAD33-3xFLAG-nfuA | Arabinose-inducible expression of 3xFLAG-NfuA | This work |
| pBAD33-3xFLAG-sufD | Arabinose-inducible expression of 3xFLAG-SufD | This work |
| pBAD33-3xFLAG-iscU | Arabinose-inducible expression of 3xFLAG-IscU | This work |
| pBAD33-iscA-3xFLAG | Arabinose-inducible expression of IscA-3xFLAG | This work |
| pBAD33-erpA-3xFLAG | Arabinose-inducible expression of ErpA-3xFLAG | This work |
| pBAD33-sufA-3xFLAG | Arabinose-inducible expression of SufA-3xFLAG | This work |
| pBAD33-nfuA-3xFLAG | Arabinose-inducible expression of NfuA-3xFLAG | This work |
| pBAD33-sufD-3xFLAG | Arabinose-inducible expression of SufD-3xFLAG | This work |
| pBAD33-iscU-3xFLAG | Arabinose-inducible expression of IscU-3xFLAG | This work |
| pBADP-NLL-MetRS | NLL-MetRS expression vector | (8) |
| pBAD33-NLL-MetRS | Arabinose-inducible expression of NLL-MetRS | This work |
| pBAD33-SD-3xFLAG-NLL-MetRS | Arabinose-inducible expression of 3xFLAG-NLL-MetRS | This work |
| pUC18T-mini-Tn7T-PrpoS-gfp | Vector encoding transcriptional fusion of GFPmut3b with the *P. aeruginosa* promoter for *rpoS* | (8) |
| pBAD33-dRBS-GFP-NLL-MetRS | Arabinose-inducible expression of GFP-3xFLAG-NLL-MetRS | This work |
| pBbS5k-dRBS-GFP-NLL-MetRS | Low-copy expression vector for GFP-3xFLAG-NLL-MetRS | This work |
| PrrnBp1-dRBS-GFP - NLL-MetRS | Low-copy expression vector for stringent expression of GFP-NLL-MetRS fusion driven by *rrnB* P1 promoter | This work |
| pBbA2c | Medium-copy expression vector for anhydrotetracycline-inducible expression | (4) |

| | | |
|---|---|---|
| pBbA2c-3xFLAG-BolA | Anhydrotetracycline-inducible expression of 3xFLAG-BolA | This work |
| pBbA2c-3xFLAG-NarP | Anhydrotetracycline-inducible expression of 3xFLAG-NarP | This work |
| pBAD33-rpoZ-3xFLAG | Arabinose-inducible expression of RpoZ-3xFLAG | This work |
| pBAD33-bolA-3xFLAG | Arabinose-inducible expression of BolA-3xFLAG | This work |
| pBAD33-glaH-3xFLAG | Arabinose-inducible expression of GlaH-3xFLAG | This work |
| pBAD33-bssR-3xFLAG | Arabinose-inducible expression of BssR-3xFLAG | This work |
| pBAD33-uacR-3xFLAG | Arabinose-inducible expression of UacR-3xFLAG | This work |
| pBAD33-csiE-3xFLAG | Arabinose-inducible expression of CsiE-3xFLAG | This work |
| pBAD33-frdB-3xFLAG | Arabinose-inducible expression of FrdB-3xFLAG | This work |
| pBAD33-ltnD-3xFLAG | Arabinose-inducible expression of LtnD-3xFLAG | This work |
| pBAD33-3xFLAG-rpoZ | Arabinose-inducible expression of 3xFLAG-RpoZ | This work |
| pBAD33-3xFLAG-bolA | Arabinose-inducible expression of 3xFLAG-BolA | This work |
| pBAD33-3xFLAG-glaH | Arabinose-inducible expression of 3xFLAG-GlaH | This work |
| pBAD33-3xFLAG-bssR | Arabinose-inducible expression of 3xFLAG-BssR | This work |
| pBAD33-3xFLAG-uacR | Arabinose-inducible expression of 3xFLAG-UacR | This work |
| pBAD33-3xFLAG-csiE | Arabinose-inducible expression of 3xFLAG-CsiE | This work |
| pBAD33-3xFLAG-frdB | Arabinose-inducible expression of 3xFLAG-FrdB | This work |
| pBAD33-3xFLAG-ltnD | Arabinose-inducible expression of 3xFLAG-LtnD | This work |
| pBAD33-3xFLAG-gadX | Arabinose-inducible expression of 3xFLAG-GadX | This work |
| pBAD33-3xFLAG-nsrR | Arabinose-inducible expression of 3xFLAG-NsrR | This work |
| pBAD33-3xFLAG-fliZ | Arabinose-inducible expression of 3xFLAG-FliZ | This work |
| pBAD33-3xFLAG-csgD | Arabinose-inducible expression of 3xFLAG-CsgD | This work |
| pBAD33-3xFLAG-fecI | Arabinose-inducible expression of 3xFLAG-FecI | This work |
| pBAD33-3xFLAG-iraD | Arabinose-inducible expression of 3xFLAG-IraD | This work |
| pBAD33-3xFLAG-yeiL | Arabinose-inducible expression of 3xFLAG-YeiL | This work |
| pBAD33-3xFLAG-fliS | Arabinose-inducible expression of 3xFLAG-FliS | This work |
| pBAD33-gadX-3xFLAG | Arabinose-inducible expression of GadX-3xFLAG | This work |
| pBAD33-nsrR-3xFLAG | Arabinose-inducible expression of NsrR-3xFLAG | This work |
| pBAD33-fliZ-3xFLAG | Arabinose-inducible expression of FliZ-3xFLAG | This work |

| | | |
|---|---|---|
| pBAD33-csgD-3xFLAG | Arabinose-inducible expression of CsgD-3xFLAG | This work |
| pBAD33-fecI-3xFLAG | Arabinose-inducible expression of FecI-3xFLAG | This work |
| pBAD33-iraD-3xFLAG | Arabinose-inducible expression of IraD-3xFLAG | This work |
| pBAD33-yeiL-3xFLAG | Arabinose-inducible expression of YeiL-3xFLAG | This work |
| pBAD33-fliS-3xFLAG | Arabinose-inducible expression of FliS-3xFLAG | This work |

**Table S5. Primers used in this study.**

| Primer | Sequence | Purpose |
|---|---|---|
| pKD13-lon-fwd | TTTTTATTAGTGCATTTTGCGCGAGGTCACTATTTTGCAGTCACAACCTGTGTAGGCTGGAGCTGCTTCG | Amplification of pKD13 resistance cassette for Lon deletion |
| pKD13-lon-rev | TCGTGTCATCTGATTACCTGGCGGAAATTAAACTAAGAGAGAGCTCTATGATTCCGGGGATCCGTCGACC | Amplification of pKD13 resistance cassette for Lon deletion |
| pKD13-clpP-fwd | AGTATAGCGGCACAGTTGCGCCTCTGGCATCAATTACGATGGGTCAGAATTGTAGGCTGGAGCTGCTTCG | Amplification of pKD13 resistance cassette for ClpP deletion |
| pKD13-clpP-rev | TTACAATCGGTACAGCAGGTTTTTTCAATTTTATCCAGGAGACGGAAATGATTCCGGGGATCCGTCGACC | Amplification of pKD13 resistance cassette for ClpP deletion |
| pKD13-hslV-fwd | CGTGGGGTCATTTCAGACATGGGAGATCCTTACGCTTTGTAGCTTAATTCTGTAGGCTGGAGCTGCTTCG | Amplification of pKD13 resistance cassette for HslV deletion |
| pKD13-hslV-rev | TTTTGTACGGGGTTTGTACTCTGTATTCGTAACCAAGGGGTCAGCTCGTGATTCCGGGGATCCGTCGACC | Amplification of pKD13 resistance cassette for HslV deletion |
| pKD13-clpA-fwd | GGACTTGACCAACCTACCTAACAATCAGATTAATGCGCTGCTTCCGCCTTTGTAGGCTGGAGCTGCTTCG | Amplification of pKD13 resistance cassette for ClpA deletion |
| pKD13-clpA-rev | CGCTAGAAAAAGCCTGAATGCAGGCATAAAAATTGGGGGAGGTGCCTATGATTCCGGGGATCCGTCGACC | Amplification of pKD13 resistance cassette for ClpA deletion |
| pKD13-clpX-fwd | TGGTTAACTAATTGTATGGGAATGGTTAATTATTCACCAGATGCCTGTTGTGTAGGCTGGAGCTGCTTCG | Amplification of pKD13 resistance cassette for ClpX deletion |

| | | |
|---|---|---|
| pKD13-clpX-rev | TTGCGTCGTCGTGTGCGGCACAAAGAACAAAGAAGAGGTTTTGACCCATGATTCCGGGGATCCGTCGACC | Amplification of pKD13 resistance cassette for ClpX deletion |
| pKD13-csgB-fwd | ATCGGATTGATTTAAAAGTCGAATGGAAATTAACGTTGTGTCACGCGAATTGTAGGCTGGAGCTGCTTCG | Amplification of pKD13 resistance cassette for CsgB deletion |
| pKD13-csgB-rev | GAAATGATTTAATTTCTTAAATGTACGACCAGGTCCAGGGTGACAACATGATTCCGGGGATCCGTCGACC | Amplification of pKD13 resistance cassette for CsgB deletion |
| pKD13-pdeH-fwd | TAGTCCAGCCAGGCGGAAAATGAGGCAGCTTATAGCGCCAGAACCGCCGTTGTAGGCTGGAGCTGCTTCG | Amplification of pKD13 resistance cassette for PdeH deletion |
| pKD13-pdeH-rev | CTTTGTCGAGTCCGGGCAGCATCACTTTTAAACACAGGACATCTTTGATGATTCCGGGGATCCGTCGACC | Amplification of pKD13 resistance cassette for PdeH deletion |
| clpX-seq-fwd | TTCGATTCTGACCCATCGTAATTGATG | Deletion screening for ClpX |
| clpX-seq-rev | GATGACACGACTGTGCTTCACG | Deletion screening for ClpX |
| clpP-seq-fwd | AAGCCTCTTTCGGTGTTAGCG | Deletion screening for ClpP |
| clpP-seq-rev | GCCATCTTTGCGTTTATCTGTCATGG | Deletion screening for ClpP |
| lon-seq-fwd | CTCTATTCTCGGCGTTGAATGTGG | Deletion screening for Lon |
| lon-seq-rev | GCAGTTATATCAGGCCAGCCATC | Deletion screening for Lon |
| hslV-seq-fwd | CAATCGGTTGAAGATGGCGGG | Deletion screening for HslV |
| hslV-seq-rev | CATGGCGCAGCTCTTCGTTG | Deletion screening for HslV |
| clpA-seq-fwd | GCGGAGTCTTTACCGCCGAG | Deletion screening for ClpA |
| clpA-seq-rev | TCCTTACCTTCCCGCAGCAC | Deletion screening for ClpA |

| | | |
|---|---|---|
| csgB-seq-fwd | AAATACAACGCGCGGGTGAG T | Deletion screening for CsgB |
| csgB-seq-rev | CCGCCACCACCGTGGTTA | Deletion screening for CsgB |
| pdeH-seq-fwd | GAGCCAGAACGTATTCCTGA AAGATTCC | Deletion screening for PdeH |
| pdeH-seq-rev | TGCAATCAAAATGATCGCCAC GAC | Deletion screening for PdeH |
| pdeH-B-FLAG-C-fwd | atacggcggttctggcgctaGACTACA AGGACGACGACGAC | Backbone amplification of pBAD33-FLAG for PdeH-FLAG expression vector |
| pdeH-B-FLAG-C-rev | cgctggataacctgccttatCATcgtttca ctcctggccttc | Backbone amplification of pBAD33-FLAG for PdeH-FLAG expression vector |
| pdeH-FLAG-C-fwd | aggccaggagtgaaacgATGataagg caggttatccagcgaataag | Chromosomal amplification of PdeH for PdeH-FLAG insert |
| pdeH-FLAG-C-rev | TCGTCGTCGTCCTTGTAGTCt agcgccagaaccgcc | Chromosomal amplification of PdeH for PdeH-FLAG insert |
| pdeH-B-FLAG-N-fwd | ATACGGCGGTTCTGGCGCTA TGAgtcgacctgcaggc | Backbone amplification of pBAD33-FLAG for FLAG-PdeH expression vector |
| pdeH-B-FLAG-N-rev | CGCTGGATAACCTGCCTTATC TTGTCGTCGTCGTCCTTGTAG | Backbone amplification of pBAD33-FLAG for FLAG-PdeH expression vector |
| pdeH-FLAG-N-fwd | ACAAGGACGACGACGACAAG ataaggcaggttatccagcgaataag | Chromosomal amplification of PdeH for FLAG-PdeH insert |
| pdeH-FLAG-N-rev | catgcctgcaggtcgacTCAtagcgcca gaaccgcc | Chromosomal amplification of PdeH for FLAG-PdeH insert |
| ΔN(2-7)-pdeH-fwd | /5Phos/cgaataagcaaccctgaagca ag | Truncation of residues 2-7 in pBAD33-pdeH-FLAG |
| ΔN(2-13)-pdeH-fwd | /5Phos/gcaagcatcgagagcttgc | Truncation of residues 2-13 in pBAD33-pdeH-FLAG |
| ΔN(2-19)-pdeH-fwd | /5Phos/caggaacggcgtttttggttg | Truncation of residues 2-19 in pBAD33-pdeH-FLAG |
| ΔN(2-25)-pdeH-fwd | /5Phos/ttgcagtgtgagcgtgc | Truncation of residues 2-25 in pBAD33-pdeH-FLAG |

| | | |
|---|---|---|
| ΔN-pdeH-rev | /5Phos/CATcgtttcactcctggcc | Backbone amplification for N-terminal truncation in pBAD33-pdeH-FLAG |
| pdeH-I2A-fwd | /5Phos/GCGaggcaggttatccagcgaataag | Site-directed mutagenesis of pBAD33-pdeH-FLAG |
| pdeH-I2A-rev | /5Phos/CATcgtttcactcctggcct | Site-directed mutagenesis of pBAD33-pdeH-FLAG |
| pdeH-R3A-fwd | /5Phos/GCGcaggttatccagcgaataagcaac | Site-directed mutagenesis of pBAD33-pdeH-FLAG |
| pdeH-R3A-rev | /5Phos/tatCATcgtttcactcctggcc | Site-directed mutagenesis of pBAD33-pdeH-FLAG |
| pdeH-Q4A-fwd | /5Phos/GCGgttatccagcgaataagcaaccc | Site-directed mutagenesis of pBAD33-pdeH-FLAG |
| pdeH-Q4A-rev | /5Phos/ccttatCATcgtttcactcctgg | Site-directed mutagenesis of pBAD33-pdeH-FLAG |
| pdeH-V5A-fwd | /5Phos/GCGatccagcgaataagcaaccctg | Site-directed mutagenesis of pBAD33-pdeH-FLAG |
| pdeH-V5A-rev | /5Phos/ctgccttatCATcgtttcactcc | Site-directed mutagenesis of pBAD33-pdeH-FLAG |
| pdeH-I6A-fwd | /5Phos/GCGcagcgaataagcaaccctgaag | Site-directed mutagenesis of pBAD33-pdeH-FLAG |
| pdeH-I6A-rev | /5Phos/aacctgccttatCATcgtttcac | Site-directed mutagenesis of pBAD33-pdeH-FLAG |
| pdeH-Q7A-fwd | /5Phos/GCGcgaataagcaaccctgaagcaag | Site-directed mutagenesis of pBAD33-pdeH-FLAG |
| pdeH-Q7A-rev | /5Phos/gataacctgccttatCATcgtttcac | Site-directed mutagenesis of pBAD33-pdeH-FLAG |
| pdeH-R8A-fwd | /5Phos/GCGataagcaaccctgaagcaagc | Site-directed mutagenesis of pBAD33-pdeH-FLAG |
| pdeH-R8A-rev | /5Phos/ctggataacctgccttatCATcgtttc | Site-directed mutagenesis of pBAD33-pdeH-FLAG |
| pdeH-I9A-fwd | /5Phos/GCGagcaaccctgaagcaagc | Site-directed mutagenesis of pBAD33-pdeH-FLAG |
| pdeH-I9A-rev | /5Phos/tcgctggataacctgccttatC | Site-directed mutagenesis of pBAD33-pdeH-FLAG |
| pdeH-S10A-fwd | /5Phos/GCGaaccctgaagcaagcatcgag | Site-directed mutagenesis of pBAD33-pdeH-FLAG |

| | | |
|---|---|---|
| pdeH-S10A-rev | /5Phos/tattcgctggataacctgccttat C | Site-directed mutagenesis of pBAD33-pdeH-FLAG |
| pdeH-N11A-fwd | /5Phos/GCGcctgaagcaagcatcga gagc | Site-directed mutagenesis of pBAD33-pdeH-FLAG |
| pdeH-N11A-rev | /5Phos/gcttattcgctggataacctgcc | Site-directed mutagenesis of pBAD33-pdeH-FLAG |
| pdeH-P12A-fwd | /5Phos/GCGgaagcaagcatcgaga gcttg | Site-directed mutagenesis of pBAD33-pdeH-FLAG |
| pdeH-P12A-rev | /5Phos/gttgcttattcgctggataacctg | Site-directed mutagenesis of pBAD33-pdeH-FLAG |
| pdeH-E13A-fwd | /5Phos/GCGgcaagcatcgagagctt gc | Site-directed mutagenesis of pBAD33-pdeH-FLAG |
| pdeH-E13A-rev | /5Phos/agggttgcttattcgctggataac | Site-directed mutagenesis of pBAD33-pdeH-FLAG |
| pdeH-S15A-fwd | /5Phos/GCGatcgagagcttgcagga acg | Site-directed mutagenesis of pBAD33-pdeH-FLAG |
| pdeH-S15A-rev | /5Phos/tgcttcagggttgcttattcgc | Site-directed mutagenesis of pBAD33-pdeH-FLAG |
| pdeH-I16A-fwd | /5Phos/GCGgagagcttgcaggaacg gc | Site-directed mutagenesis of pBAD33-pdeH-FLAG |
| pdeH-I16A-rev | /5Phos/gcttgcttcagggttgcttattcg | Site-directed mutagenesis of pBAD33-pdeH-FLAG |
| pdeH-E17A-fwd | /5Phos/GCGagcttgcaggaacggcg | Site-directed mutagenesis of pBAD33-pdeH-FLAG |
| pdeH-E17A-rev | /5Phos/gatgcttgcttcagggttgct | Site-directed mutagenesis of pBAD33-pdeH-FLAG |
| pdeH-S18A-fwd | /5Phos/GCGttgcaggaacggcgttttt g | Site-directed mutagenesis of pBAD33-pdeH-FLAG |
| pdeH-S18A-rev | /5Phos/ctcgatgcttgcttcagggttg | Site-directed mutagenesis of pBAD33-pdeH-FLAG |
| pdeH-L19A-fwd | /5Phos/GCGcaggaacggcgtttttggt tg | Site-directed mutagenesis of pBAD33-pdeH-FLAG |
| pdeH-L19A-rev | /5Phos/gctctcgatgcttgcttcagg | Site-directed mutagenesis of pBAD33-pdeH-FLAG |
| pdeH-Q20A-fwd | /5Phos/GCGgaacggcgtttttggttgc ag | Site-directed mutagenesis of pBAD33-pdeH-FLAG |

| | | |
|---|---|---|
| pdeH-Q20A-rev | /5Phos/caagctctcgatgcttgcttcag | Site-directed mutagenesis of pBAD33-pdeH-FLAG |
| pdeH-E21A-fwd | /5Phos/GCGcggcgtttttggttgcagtg | Site-directed mutagenesis of pBAD33-pdeH-FLAG |
| pdeH-E21A-rev | /5Phos/ctgcaagctctcgatgcttgc | Site-directed mutagenesis of pBAD33-pdeH-FLAG |
| pdeH-X(2-7)A-fwd | /5Phos/GCCGCAGCGcgaataag caaccctgaagcaagc | Site-directed mutagenesis of pBAD33-pdeH-FLAG |
| pdeH-X(2-7)A-rev | /5Phos/GGCGGCTGCCATcgtttc actcctggccttc | Site-directed mutagenesis of pBAD33-pdeH-FLAG |
| pdeH-X(8-13)A-fwd | /5Phos/GCCGCAGCGgcaagcat cgagagcttgca | Site-directed mutagenesis of pBAD33-pdeH-FLAG |
| pdeH-X(8-13)A-rev | /5Phos/GGCGGCTGCctggataac ctgccttatCATcgtttc | Site-directed mutagenesis of pBAD33-pdeH-FLAG |
| pdeH-X(15-19)A-fwd | /5Phos/GCCGCAGCGcaggaacg gcgtttttggttg | Site-directed mutagenesis of pBAD33-pdeH-FLAG |
| pdeH-X(15-19)A-rev | /5Phos/GGCGGCTGCttcagggttg cttattcgctgga | Site-directed mutagenesis of pBAD33-pdeH-FLAG |
| pdeH-X(20-25)A-fwd | /5Phos/GCCGCAGCGttgcagtgtg agcgtgcttac | Site-directed mutagenesis of pBAD33-pdeH-FLAG |
| pdeH-X(20-25)A-rev | /5Phos/GGCGGCTGCcaagctctc gatgcttgcttcag | Site-directed mutagenesis of pBAD33-pdeH-FLAG |
| pdeH-F1-fwd | ATGTAGTAAAGGCCCGGCAG AC | Donor DNA construction of PdeH-3xFLAG flanked by 500 bp homology |
| pdeH-F1-rev | GCTCAATCTGACGCAGGATTT TTGG | Donor DNA construction of PdeH-3xFLAG flanked by 500 bp homology |
| pdeH-F2-fwd | CCAAAAATCCTGCGTCAGATT GAGC | Donor DNA construction of PdeH-3xFLAG flanked by 500 bp homology |
| pdeH-F2-rev | TAGCGCCAGAACCGCCG | Donor DNA construction of pdeH-3xFLAG flanked by 500 bp homology |
| pdeH-F3-fwd | ATACGGCGGTTCTGGCGCTA AGCGGTGGCGGCG | Donor DNA construction of PdeH-3xFLAG flanked by 500 bp homology |

| | | |
|---|---|---|
| pdeH-F3-rev | GCGGAAAATGAGGCAGCttaCTTATATAACTCGTCCATGCCATGAGTG | Donor DNA construction of PdeH-3xFLAG flanked by 500 bp homology |
| pdeH-F4-fwd | taaGCTGCCTCATTTTCCGCCTG | Donor DNA construction of PdeH-3xFLAG flanked by 500 bp homology |
| pdeH-F4-rev | AGATTAAACGTCCAGTTATTGTTCTTTTCAGAGAG | Donor DNA construction of PdeH-3xFLAG flanked by 500 bp homology |
| B-pdeH-frag-fwd | AATAACTGGACGTTTAATCTTtcgagttcatgtgcagctcc | Insertion of PdeH-3xFLAG donor DNA into pTarget-pdeH(N20) |
| B-pdeH-frag-rev | CTGCCGGGCCTTTACTACATgtagggataacagggtaatagatctaagc | Insertion of PdeH-3xFLAG donor DNA into pTarget-pdeH(N20) |
| pdeH-val-fwd | GACTGCTCACTCTCCAGCCAG | Validation of PdeH-3xFLAG insertion at *pdeH* locus |
| pdeH-val-rev | GTCATCAATGGCGATCCGCC | Validation of PdeH-3xFLAG insertion at *pdeH* locus |
| yhjHb3525_366 N20 fwd | /5Phos/GCCAGGGAAGACGCTCAATCgttttagagctagaaatagcaagttaaaataagg | Replacement of N20 for targeting *pdeH* locus in pTarget to construct pTarget-pdeH(N20) |
| pTarget-N20-ins-rev | /5Phos/actagtattatacctaggactgagctag | Replacement of N20 for targeting *pdeH* locus in pTarget to construct pTarget-pdeH(N20) |
| F1-pdeH-X(15-19)A-fwd | ATGTAGTAAAGGCCCGGCAGAC | Amplification of pTarget-pdeH-3xFLAG for construction of PdeH(X(15-19)A)-3xFLAG donor DNA |
| F1-pdeH-X(15-19)A-rev | CGCTGCGGCGGCGGCTGCTTCAGGGTTGCTTATTCGCTGG | Amplification of pTarget-pdeH-3xFLAG for construction of PdeH(X(15-19)A)-3xFLAG donor DNA |
| F2-pdeH-X(15-19)A-fwd | GCAGCCGCCGCCGCAGCGCAGGAACGGCGTTTTTGGTTG | Amplification of pTarget-pdeH-3xFLAG for construction of PdeH(X(15-19)A)-3xFLAG donor DNA |
| F2-pdeH-X(15-19)A-rev | AGATTAAACGTCCAGTTATTGTTCTTTTCAGAGAG | Amplification of pTarget-pdeH-3xFLAG for construction of |

| | | |
|---|---|---|
| | | PdeH(X(15-19)A)-3xFLAG donor DNA |
| E48A-F1-fwd | GGCCGTGGCGCTATTAACG | Amplification of PdeH(E48A) for construction of pTarget-pdeH(E48A) |
| E48A-F1-rev | TAGCGCCAGAACCGCC | Amplification of PdeH(E48A) for construction of pTarget-pdeH(E48A) |
| E48A-B-fwd | ATACGGCGGTTCTGGCGCTAtaaGCTGCCTCATTTTCCGCC | Backbone amplification of pTarget-pdeH-3xFLAG for construction of pTarget-pdeH(E48A) |
| E48A-B-rev | CGTTAATAGCGCCACGGCC | Backbone amplification of pTarget-pdeH-3xFLAG for construction of pTarget-pdeH(E48A) |
| F1-F3-rev | TAGCGCCAGAACCGCCG | Chromosomal amplification of MG1655 *pdeH(L19A)-3xFLAG* and MG1655 *pdeH(X(15-19)A)-3xFLAG* with pdeH-F1-fwd for construction of PdeH(L19A) and PdeH(X(15-19)A) donor DNA, respectively |
| B-F4-fwd | GCGGTTCTGGCGCTAtaaGCTGCCTCATTTTCCGCC | Chromosomal amplification of MG1655 *pdeH(L19A)-3xFLAG* and MG1655 *pdeH(X(15-19)A)-3xFLAG* with pdeH-F4-rev for construction of PdeH(L19A) and PdeH(X(15-19)A) donor DNA, respectively |
| B-R-ins-3xFLAG-rev | catcgtttcactcctggccttc | Backbone amplification of pBAD33 for C-terminal 3xFLAG insertion |
| B-R-ins-3xFLAG-fwd | GACTATAAGGATCATGATGGGGATTACAAGGATCATGATATCGATTACAAAGACGACGACGATAAAtgagtcgacctgcaggcatg | Backbone amplification of pBAD33 for C-terminal 3xFLAG insertion |
| B-R-3xFLAG-ins-rev | TTTATCGTCGTCGTCTTTGTAATCGATATCATGATCCTTGTAATCCCCATCATGATCCTTATAGTCcatcgtttcactcctggccttc | Backbone amplification of pBAD33 for N-terminal 3xFLAG insertion |

| | | |
|---|---|---|
| B-R-3xFLAG-ins-fwd | tgagtcgacctgcaggcatg | Backbone amplification of pBAD33 for N-terminal 3xFLAG insertion |
| 3xFLAG-clpS-fwd | acaaagacgacgacgataaaGGTAAAACGAACGACTGGCTGG | *clpS* insertion into pBAD33 with N-terminal 3xFLAG |
| 3xFLAG-clpS-rev | catgcctgcaggtcgactcaGGCTTTTTCTAGCGTACACAGCAATG | *clpS* insertion into pBAD33 with N-terminal 3xFLAG |
| clpS-3xFLAG-fwd | aggccaggagtgaaacgatgGGTAAAACGAACGACTGGCTGG | *clpS* insertion into pBAD33 with C-terminal 3xFLAG |
| clpS-3xFLAG-rev | ccatcatgatccttatagtcGGCTTTTTCTAGCGTACACAGCAATG | *clpS* insertion into pBAD33 with C-terminal 3xFLAG |
| ClpS-NTD-rev | /5Phos/catcgtttcactcctggccttcg | Site-directed mutagenesis of pBAD33-clpS-3xFLAG for NTE removal |
| ClpS-NTD-fwd | /5Phos/TCTATGTATAAAGTGATATTAGTCAATGATGATTACACTCCG | Site-directed mutagenesis of pBAD33-clpS-3xFLAG for NTE removal |
| 3xFLAG-iscA-fwd | acaaagacgacgacgataaaTCGATTACACTGAGCGACAGTGC | *iscA* insertion into pBAD33 with N-terminal 3xFLAG |
| 3xFLAG-iscA-rev | catgcctgcaggtcgactcaAACGTGGAAGCTTTCGCCGC | *iscA* insertion into pBAD33 with N-terminal 3xFLAG |
| 3xFLAG-erpA-fwd | acaaagacgacgacgataaaAGTGATGACGTAGCACTGCCG | *erpA* insertion into pBAD33 with N-terminal 3xFLAG |
| 3xFLAG-erpA-rev | catgcctgcaggtcgactcaGATACTAAAGGAAGAACCGCAACCGC | *erpA* insertion into pBAD33 with N-terminal 3xFLAG |
| 3xFLAG-sufA-fwd | acaaagacgacgacgataaaGACATGCATTCAGGAACCTTTAACCC | *sufA* insertion into pBAD33 with N-terminal 3xFLAG |
| 3xFLAG-sufA-rev | catgcctgcaggtcgactcaTACCCCAAAGCTTTCGCCACAG | *sufA* insertion into pBAD33 with N-terminal 3xFLAG |
| 3xFLAG-nfuA-fwd | acaaagacgacgacgataaaATCCGTATTTCCGATGCTGCACAAG | *nfuA* insertion into pBAD33 with N-terminal 3xFLAG |
| 3xFLAG-nfuA-rev | catgcctgcaggtcgactcaGTAGTAGGAGTGTTCGCCGCG | *nfuA* insertion into pBAD33 with N-terminal 3xFLAG |
| iscA-3xFLAG-fwd | aggccaggagtgaaacgatgTCGATTACACTGAGCGACAGTGC | *iscA* insertion into pBAD33 with C-terminal 3xFLAG |
| iscA-3xFLAG-rev | ccatcatgatccttatagtcAACGTGGAAGCTTTCGCCGC | *iscA* insertion into pBAD33 with C-terminal 3xFLAG |

| | | |
|---|---|---|
| erpA-3xFLAG-fwd | aggccaggagtgaaacgatgAGTGATGACGTAGCACTGCCG | *erpA* insertion into pBAD33 with C-terminal 3xFLAG |
| erpA-3xFLAG-rev | ccatcatgatccttatagtcGATACTAAAGGAAGAACCGCAACCGC | *erpA* insertion into pBAD33 with C-terminal 3xFLAG |
| sufA-3xFLAG-fwd | aggccaggagtgaaacgatgGACATGCATTCAGGAACCTTTAACCC | *sufA* insertion into pBAD33 with C-terminal 3xFLAG |
| sufA-3xFLAG-rev | ccatcatgatccttatagtcTACCCCAAAGCTTTCGCCACAG | *sufA* insertion into pBAD33 with C-terminal 3xFLAG |
| nfuA-3xFLAG-fwd | aggccaggagtgaaacgatgATCCGTATTTCCGATGCTGCACAAG | *nfuA* insertion into pBAD33 with C-terminal 3xFLAG |
| nfuA-3xFLAG-rev | ccatcatgatccttatagtcGTAGTAGGAGTGTTCGCCGCG | *nfuA* insertion into pBAD33 with C-terminal 3xFLAG |
| NLL_KpnI_for | attcAAATggtACcATGAGAGGATCGCATCACCA | Amplification of NLL-MetRS from pBADP-NLL-MetRS for insertion into pBAD33 |
| NLL_SalI_rev | catgcctgcaggtcgacTCA | Amplification of NLL-MetRS from pBADP-NLL-MetRS for insertion into pBAD33 |
| SD forward | /5Phos/GGCCACGAAGGCCAGGAGTGAAACGatgagaggatcgcatcaccatcac | Insertion of Shine-Dalgarno sequence into pBAD33-NLL-MetRS |
| SD rev | /5Phos/ggtaccgagctcgaattcgctag | Insertion of Shine-Dalgarno sequence into pBAD33-NLL-MetRS |
| 3x FLAG forward | /5Phos/ATGGACTACAAGGACCACGACGGTGACTACAAGGACCACGACATCGACTACAAGGACGACGACGACAAGatgactcaagtcgcgaag | Insertion of 3xFLAG into pBAD33-NLL-MetRS |
| FLAG rev | /5Phos/cgtttcactcctggcc | Insertion of 3xFLAG into pBAD33-NLL-MetRS |
| pBAD33_rev | GGGACAACTCCAGTGAAAAGTTCTTCTCCTTTACGCATcgtttcactcctggccttcgtg | Insertion of GFPmut3b into pBAD33-SD-3xFLAG-NLL-MetRS |
| Gfp_fwd | GTACCGGCCACGAAGGCCAGGAGTGAAACGatgcgtaaaggagaagaacttttcactgg | Insertion of GFPmut3b into pBAD33-SD-3xFLAG-NLL-MetRS |

| | | |
|---|---|---|
| Gfp_rev | TAGTCACCGTCGTGGTCCTTGTAGTCCATtttgtatagttcatccatgccatgtgtaatc | Insertion of GFPmut3b into pBAD33-SD-3xFLAG-NLL-MetRS |
| 3xF-NLL_fwd | ATTACACATGGCATGGATGAACTATACAAAatggactacaaggaccacgacg | Insertion of GFPmut3b into pBAD33-SD-3xFLAG-NLL-MetRS |
| 5P-dRBS-rev | /5Phos/TTTTGGCCGACTTATAATTGTCggtaccgagctcgaattcgc | Insertion of dRBS to construct pBAD33-dRBS-GFP-NLL-MetRS |
| 5P-dRBS-fwd | /5Phos/AACGACACTATTAAGGAGGCATTTTatgcgtaaaggagaagaacttttcac | Insertion of dRBS to construct pBAD33-dRBS-GFP-NLL-MetRS |
| B-pBbS5k-dRBS-rev | TTGGCCGACTTATAATTGTCaaagttaaggatcccagatcgc | Insertion of dRBS-GFP-3xFLAG-NLL-MetRS into pBbS5k backbone |
| B-dRBS-NLL-fwd | TGGTGGAAGCCTCTAAATGAtagggatccaaactcgagtaag | Insertion of dRBS-GFP-3xFLAG-NLL-MetRS into pBbS5k backbone |
| I-pBbS5k-dRBS-fwd | GACAATTATAAGTCGGCCAAAAAACG | Insertion of dRBS-GFP-3xFLAG-NLL-MetRS into pBbS5k backbone |
| I-dRBS-NLL-rev | TCATTTAGAGGCTTCCACCAG | Insertion of dRBS-GFP-3xFLAG-NLL-MetRS into pBbS5k backbone |
| rrnB-dRBS-fwd | /5Phos/AGGCCGGAATAACTCCCTATAATGCGCCACCACTgacaattataagtcggccaaaaaacg | Insertion of rrnB P1 promoter to construct PrrnBp1-dRBS-GFP-NLL-MetRS |
| rrnB-dRBS-rev | /5Phos/GACAAGAGGAAATTTAAAATAATTTTCTGACCGCGCAACgcgcaacgcaattaatgtaagttag | Insertion of rrnB P1 promoter to construct PrrnBp1-dRBS-GFP-NLL-MetRS |
| B-A2c-3xFLAG-ins-fwd | TAAGGATCCAAACTCGAGTAAGGATCTCCAGG | Insertion of 3xFLAG-BolA and 3xFLAG-NarP into pBbA2c |
| B-A2c-3xFLAG-ins-rev | CTTGTCGTCGTCGTCCTTGTAGTCGATG | Insertion of 3xFLAG-BolA and 3xFLAG-NarP into pBbA2c |
| 3xFLAG-glaH-fwd | acaaagacgacgacgataaaAATGCACTGACCGCCGTAC | *glaH* insertion into pBAD33 with N-terminal 3xFLAG |
| 3xFLAG-glaH-rev | catgcctgcaggtcgactcaCTGATGCGTCTGGTAGTGGTTAGAG | *glaH* insertion into pBAD33 with N-terminal 3xFLAG |

| | | |
|---|---|---|
| 3xFLAG-csiE-fwd | acaaagacgacgacgataaaATGCCTACGCTTGCTCCACC | *csiE* insertion into pBAD33 with N-terminal 3xFLAG |
| 3xFLAG-csiE-rev | catgcctgcaggtcgactcaTGCTGATTCGAGCATTTTGCGG | *csiE* insertion into pBAD33 with N-terminal 3xFLAG |
| 3xFLAG-ltnD-fwd | acaaagacgacgacgataaaAAAACGGGATCTGAGTTTCATGTCGG | *ltnD* insertion into pBAD33 with N-terminal 3xFLAG |
| 3xFLAG-ltnD-rev | catgcctgcaggtcgactcaTGATTTCGCTCCCGGTAGAGTG | *ltnD* insertion into pBAD33 with N-terminal 3xFLAG |
| 3xFLAG-uacR-fwd | acaaagacgacgacgataaaGAGCTTGCTACTACGCAGTCAGTATTG | *uacR* insertion into pBAD33 with N-terminal 3xFLAG |
| 3xFLAG-uacR-rev | catgcctgcaggtcgactcaTGTGTTTAACAACTCATATTTCTTAATCTTGCGATAGAG | *uacR* insertion into pBAD33 with N-terminal 3xFLAG |
| 3xFLAG-frdB-fwd | acaaagacgacgacgataaaGCTGAGATGAAAAACCTGAAAATTGAGGTG | *frdB* insertion into pBAD33 with N-terminal 3xFLAG |
| 3xFLAG-frdB-rev | catgcctgcaggtcgactcaGCGTGGTTTCAGGGTCGC | *frdB* insertion into pBAD33 with N-terminal 3xFLAG |
| 3xFLAG-fadH-fwd | acaaagacgacgacgataaaAGCTACCCGTCGCTGTTCG | *fadH* insertion into pBAD33 with N-terminal 3xFLAG |
| 3xFLAG-fadH-rev | catgcctgcaggtcgactcaAATCTCCAGCGCCAGCCG | *fadH* insertion into pBAD33 with N-terminal 3xFLAG |
| 3xFLAG-bolA-fwd | acaaagacgacgacgataaaATGATACGTGAGCGGATAGAAGAAAAATTAAG | *bolA* insertion into pBAD33 with N-terminal 3xFLAG |
| 3xFLAG-bolA-rev | catgcctgcaggtcgactcaCGCGATGCTTCCTGCTCCA | *bolA* insertion into pBAD33 with N-terminal 3xFLAG |
| 3xFLAG-bssR-fwd | acaaagacgacgacgataaaTTCGTTGACAGACAGCGAATCGAT | *bssR* insertion into pBAD33 with N-terminal 3xFLAG |
| 3xFLAG-bssR-rev | catgcctgcaggtcgactcaGGCCTTCTCAAGCATGGCG | *bssR* insertion into pBAD33 with N-terminal 3xFLAG |
| 3xFLAG-rpoZ-fwd | acaaagacgacgacgataaaGCACGCGTAACTGTTCAGGAC | *rpoZ* insertion into pBAD33 with N-terminal 3xFLAG |
| 3xFLAG-rpoZ-rev | catgcctgcaggtcgactcaACGACGACCTTCAGCAATAGCG | *rpoZ* insertion into pBAD33 with N-terminal 3xFLAG |

| | | |
|---|---|---|
| glaH-3xFLAG-fwd | aggccaggagtgaaacgatgAATGCACTGACCGCCGTAC | *glaH* insertion into pBAD33 with C-terminal 3xFLAG |
| glaH-3xFLAG-rev | ccatcatgatccttatagtcCTGATGCGTCTGGTAGTGGTTAGAG | *glaH* insertion into pBAD33 with C-terminal 3xFLAG |
| csiE-3xFLAG-fwd | aggccaggagtgaaacgatgATGCCTACGCTTGCTCCACC | *csiE* insertion into pBAD33 with C-terminal 3xFLAG |
| csiE-3xFLAG-rev | ccatcatgatccttatagtcTGCTGATTCGAGCATTTTGCGG | *csiE* insertion into pBAD33 with C-terminal 3xFLAG |
| ltnD-3xFLAG-fwd | aggccaggagtgaaacgatgAAAACGGGATCTGAGTTTCATGTCGG | *ltnD* insertion into pBAD33 with C-terminal 3xFLAG |
| ltnD-3xFLAG-rev | ccatcatgatccttatagtcTGATTTCGCTCCCGGTAGAGTG | *ltnD* insertion into pBAD33 with C-terminal 3xFLAG |
| uacR-3xFLAG-fwd | aggccaggagtgaaacgatgGAGCTTGCTACTACGCAGTCAGTATTG | *uacR* insertion into pBAD33 with C-terminal 3xFLAG |
| uacR-3xFLAG-rev | ccatcatgatccttatagtcTGTGTTTAACAACTCATATTTCTTAATCTTGCGATAGAG | *uacR* insertion into pBAD33 with C-terminal 3xFLAG |
| frdB-3xFLAG-fwd | aggccaggagtgaaacgatgGCTGAGATGAAAAACCTGAAAATTGAGGTG | *frdB* insertion into pBAD33 with C-terminal 3xFLAG |
| frdB-3xFLAG-rev | ccatcatgatccttatagtcGCGTGGTTTCAGGGTCGC | *frdB* insertion into pBAD33 with C-terminal 3xFLAG |
| fadH-3xFLAG-fwd | aggccaggagtgaaacgatgAGCTACCCGTCGCTGTTCG | *fadH* insertion into pBAD33 with C-terminal 3xFLAG |
| fadH-3xFLAG-rev | ccatcatgatccttatagtcAATCTCCAGCGCCAGCCG | *fadH* insertion into pBAD33 with C-terminal 3xFLAG |
| bolA-3xFLAG-fwd | aggccaggagtgaaacgatgATGATACGTGAGCGGATAGAAGAAAAATTAAG | *bolA* insertion into pBAD33 with C-terminal 3xFLAG |
| bolA-3xFLAG-rev | ccatcatgatccttatagtcCGCGATGCTTCCTGCTCCA | *bolA* insertion into pBAD33 with C-terminal 3xFLAG |
| bssR-3xFLAG-fwd | aggccaggagtgaaacgatgTTCGTTGACAGACAGCGAATCGAT | *bssR* insertion into pBAD33 with C-terminal 3xFLAG |
| bssR-3xFLAG-rev | ccatcatgatccttatagtcGGCCTTCTCAAGCATGGCG | *bssR* insertion into pBAD33 with C-terminal 3xFLAG |

| | | |
|---|---|---|
| rpoZ-3xFLAG-fwd | aggccaggagtgaaacgatgGCACGCGTAACTGTTCAGGAC | *rpoZ* insertion into pBAD33 with C-terminal 3xFLAG |
| rpoZ-3xFLAG-rev | ccatcatgatccttatagtcACGACGACCTTCAGCAATAGCG | *rpoZ* insertion into pBAD33 with C-terminal 3xFLAG |
| 3xFLAG-gadX-fwd | acaaagacgacgacgataaaCAATCACTACATGGGAATTGTCTAATTGCGTATG | *gadX* insertion into pBAD33 with N-terminal 3xFLAG |
| 3xFLAG-gadX-rev | catgcctgcaggtcgactcaTAATCTTATTCCTTCCGCAGAACGGTC | *gadX* insertion into pBAD33 with N-terminal 3xFLAG |
| 3xFLAG-nsrR-fwd | acaaagacgacgacgataaaCAGTTAACGAGTTTCACTGATTACGGATTACG | *nsrR* insertion into pBAD33 with N-terminal 3xFLAG |
| 3xFLAG-nsrR-rev | catgcctgcaggtcgactcaCTCCACCAGCAATAATTTATAAAGCGGTTG | *nsrR* insertion into pBAD33 with N-terminal 3xFLAG |
| 3xFLAG-fliZ-fwd | acaaagacgacgacgataaaATGGTGCAGCACCTGAAAAGACG | *fliZ* insertion into pBAD33 with N-terminal 3xFLAG |
| 3xFLAG-fliZ-rev | catgcctgcaggtcgactcaATATATATCAGAAGAAGGCAGGCTGGAGG | *fliZ* insertion into pBAD33 with N-terminal 3xFLAG |
| 3xFLAG-csgD-fwd | acaaagacgacgacgataaaTTTAATGAAGTCCATAGTATTCATGGTCATACATTATTGTTG | *csgD* insertion into pBAD33 with N-terminal 3xFLAG |
| 3xFLAG-csgD-rev | catgcctgcaggtcgactcaTCGCCTGAGGTTATCGTTTGCC | *csgD* insertion into pBAD33 with N-terminal 3xFLAG |
| 3xFLAG-fecI-fwd | acaaagacgacgacgataaaTCTGACCGCGCCACTACC | *fecI* insertion into pBAD33 with N-terminal 3xFLAG |
| 3xFLAG-fecI-rev | catgcctgcaggtcgactcaTAACCCATACTCCAGACGGAACAGC | *fecI* insertion into pBAD33 with N-terminal 3xFLAG |
| 3xFLAG-iraD-fwd | acaaagacgacgacgataaaATGCGACAATCACTTCAGGCTGTTTTAC | *iraD* insertion into pBAD33 with N-terminal 3xFLAG |
| 3xFLAG-iraD-rev | catgcctgcaggtcgactcaGCTGACATTCTCCAGCGTCGC | *iraD* insertion into pBAD33 with N-terminal 3xFLAG |
| 3xFLAG-yeiL-fwd | acaaagacgacgacgataaaAGTGAATCCGCGTTTAAGGATTGC | *yeiL* insertion into pBAD33 with N-terminal 3xFLAG |
| 3xFLAG-yeiL-rev | catgcctgcaggtcgactcaCTGCATCATCCCGGAGAATTTATTCTCC | *yeiL* insertion into pBAD33 with N-terminal 3xFLAG |

| | | |
|---|---|---|
| 3xFLAG-fliS-fwd | acaaagacgacgacgataaaTACGCGGCAAAAGGCACCC | *fliS* insertion into pBAD33 with N-terminal 3xFLAG |
| 3xFLAG-fliS-rev | catgcctgcaggtcgactcaGACTGGGTCCTGAATCAAAGAAGGG | *fliS* insertion into pBAD33 with N-terminal 3xFLAG |
| gadX-3xFLAG-fwd | aggccaggagtgaaacgatgCAATCACTACATGGGAATTGTCTAATTGCGTATG | *gadX* insertion into pBAD33 with C-terminal 3xFLAG |
| gadX-3xFLAG-rev | ccatcatgatccttatagtcTAATCTTATTCCTTCCGCAGAACGGTC | *gadX* insertion into pBAD33 with C-terminal 3xFLAG |
| nsrR-3xFLAG-fwd | aggccaggagtgaaacgatgCAGTTAACGAGTTTCACTGATTACGGATTACG | *nsrR* insertion into pBAD33 with C-terminal 3xFLAG |
| nsrR-3xFLAG-rev | ccatcatgatccttatagtcCTCCACCAGCAATAATTTATAAAGCGGTTG | *nsrR* insertion into pBAD33 with C-terminal 3xFLAG |
| fliZ-3xFLAG-fwd | aggccaggagtgaaacgatgATGGTGCAGCACCTGAAAAGACG | *fliZ* insertion into pBAD33 with C-terminal 3xFLAG |
| fliZ-3xFLAG-rev | ccatcatgatccttatagtcATATATATCAGAAGAAGGCAGGCTGGAGG | *fliZ* insertion into pBAD33 with C-terminal 3xFLAG |
| csgD-3xFLAG-fwd | aggccaggagtgaaacgatgTTTAATGAAGTCCATAGTATTCATGGTCATACATTATTGTTG | *csgD* insertion into pBAD33 with C-terminal 3xFLAG |
| csgD-3xFLAG-rev | ccatcatgatccttatagtcTCGCCTGAGGTTATCGTTTGCC | *csgD* insertion into pBAD33 with C-terminal 3xFLAG |
| fecI-3xFLAG-fwd | aggccaggagtgaaacgatgTCTGACCGCGCCACTACC | *fecI* insertion into pBAD33 with C-terminal 3xFLAG |
| fecI-3xFLAG-rev | ccatcatgatccttatagtcTAACCCATACTCCAGACGGAACAGC | *fecI* insertion into pBAD33 with C-terminal 3xFLAG |
| iraD-3xFLAG-fwd | aggccaggagtgaaacgatgATGCGACAATCACTTCAGGCTGTTTTAC | *iraD* insertion into pBAD33 with C-terminal 3xFLAG |
| iraD-3xFLAG-rev | ccatcatgatccttatagtcGCTGACATTCTCCAGCGTCGC | *iraD* insertion into pBAD33 with C-terminal 3xFLAG |
| yeiL-3xFLAG-fwd | aggccaggagtgaaacgatgAGTGAATCCGCGTTTAAGGATTGC | *yeiL* insertion into pBAD33 with C-terminal 3xFLAG |
| yeiL-3xFLAG-rev | ccatcatgatccttatagtcCTGCATCATCCCGGAGAATTTATTCTCC | *yeiL* insertion into pBAD33 with C-terminal 3xFLAG |

| | | |
|---|---|---|
| fliS-3xFLAG-fwd | aggccaggagtgaaacgatgTACGCGGCAAAAGGCACCC | *fliS* insertion into pBAD33 with C-terminal 3xFLAG |
| fliS-3xFLAG-rev | ccatcatgatccttatagtcGACTGGGTCCTGAATCAAAGAAGGG | *fliS* insertion into pBAD33 with C-terminal 3xFLAG |

**Dataset S1. Protein instability annotations from manual curation and text mining of the PubMed and EcoCyc databases.**

This dataset contains all annotations describing direct or inferential evidence of active protein degradation for the protein.

On sheet 1 ("PubMed annotations"), Column 1 reports the PubMed ID, Column 2 reports the year of publication, Column 3 reports the journal title, Column 4 reports the title of the publication, and Column 5 reports the sentence describing evidence of protein degradation for the indicated protein labeled by NCBI Gene Symbol in Column 6.

On sheet 2 ("EcoCyc annotations"), Column 1 reports the name of the gene summary page evaluated for the presence of an annotation, Column 2 reports the description of the gene being described in the gene summary, and Column 3 reports the sentence describing evidence of protein degradation for the indicated protein labeled by NCBI Gene Symbol in Column 4.

On sheet 3 ("Manual annotations"), Column 1 reports the PubMed ID of the publication reporting evidence of instability for the protein indicated by NCBI Gene Symbol in Column 2.

On sheet 4 ("Annotated substrates"), Column 1 reports the 364 distinct annotated substrates identified by merging the text mining and manual curation datasets.

On sheet 5 ("Functional enrichment"), Column 1 reports the ontology category associated with the term in Column 2 and its description in Column 3. The foreground count, background count, enrichment score, enrichment signal, and false discovery rate as computed on the STRING enrichment server are described in Columns 4-8. The identifiers for the enriched proteins are provided in Columns 9-10.

**Dataset S2. Proteomic results for exponential phase degradation profiling.**

This dataset describes all proteins identified through LC-MS/MS analysis of BONCAT-enriched proteins from the exponential phase stability screen.

On sheet 1 ("Exponential phase (all)"), Column 1 provides the UniProtKB accession ID for each protein. Column 2 provides the corresponding NCBI Gene Symbol for the protein. Column 3 reports the number of unique peptides identified for each protein. Columns 4-18 provide the raw TMT reporter ion abundances for each sample. Column 19 provides the estimated degradation rate in base 2. Columns 20 and 21 provide the *p*-value and FDR-adjusted *p*-value for the estimated degradation rate, respectively. Columns 22 and 23 provide the lower and upper bounds for the 95% confidence interval for the linear model coefficient, respectively.

On sheet 2 ("Exponential phase (top)"), we report the 88 proteins meeting fold change and adjusted *p*-value criteria (estimated half-life < 4 h, adjusted *p*-value < 0.05) for stronger evidence of instability. Column 1 provides the UniProtKB accession ID for each protein. Column 2 provides the corresponding NCBI Gene Symbol for the protein. Column 3 reports the UniProtKB description for the protein. Column 4 provides the estimated degradation rate in base 2. Columns 5 and 6 provide the *p*-value and FDR-adjusted *p*-value for the estimated degradation rate, respectively. Columns 7 and 8 provide the lower and upper bounds for the 95% confidence interval for the linear model coefficient, respectively.

**Dataset S3. Proteomic results for stationary phase degradation profiling.**

This dataset describes all proteins identified through LC-MS/MS analysis of BONCAT-enriched proteins from the stationary phase stability screen.

On sheet 1 ("Stationary phase (all)"), Column 1 provides the UniProtKB accession ID for each protein. Column 2 provides the corresponding NCBI Gene Symbol for the protein. Column 3 reports the number of

unique peptides identified for each protein. Columns 4-18 provide the raw TMT reporter ion abundances for each sample. Column 19 provides the estimated degradation rate in base 2. Columns 20 and 21 provide the *p*-value and FDR-adjusted *p*-value for the estimated degradation rate, respectively. Columns 22 and 23 provide the lower and upper bounds for the 95% confidence interval for the linear model coefficient, respectively.

On sheet 2 ("Stationary phase (top)"), we report the 56 proteins meeting fold change and adjusted *p*-value criteria (estimated half-life < 8 h, adjusted *p*-value < 0.05) for stronger evidence of instability. Column 1 provides the UniProtKB accession ID for each protein. Column 2 provides the corresponding NCBI Gene Symbol for the protein. Column 3 reports the UniProtKB description for the protein. Column 4 provides the estimated degradation rate in base 2. Columns 5 and 6 provide the *p*-value and FDR-adjusted *p*-value for the estimated degradation rate, respectively. Columns 7 and 8 provide the lower and upper bounds for the 95% confidence interval for the linear model coefficient, respectively.

**Dataset S4. Machine learning analysis results.**

This dataset provides the machine learning training set and instability probabilities for all proteins in the analysis. Column 1 provides the UniProtKB accession ID. Column 2 provides the UniProtKB protein name. Column 3 provides the official NCBI Gene Symbol for the protein. Column 4 provides the UniProtKB protein description. Column 5 provides the instability probability produced by the trained XGBoost model. Column 6 reports the class label used during training if the indicated protein was present in the training data.

## SI References


1. K. A. Datsenko, B. L. Wanner, One-step inactivation of chromosomal genes in Escherichia coli K-12 using PCR products. *Proc Natl Acad Sci U S A* **97**, 6640–6645 (2000).

2. L. M. Guzman, D. Belin, M. J. Carson, J. Beckwith, Tight regulation, modulation, and high-level expression by vectors containing the arabinose PBAD promoter. *J Bacteriol* **177**, 4121–4130 (1995).

3. D. G. Gibson, *et al.*, Enzymatic assembly of DNA molecules up to several hundred kilobases. *Nat Methods* **6**, 343–345 (2009).

4. T. S. Lee, *et al.*, BglBrick vectors and datasheets: A synthetic biology platform for gene expression. *J Biol Eng* **5**, 12 (2011).

5. Y. Jiang, *et al.*, Multigene editing in the Escherichia coli genome via the CRISPR-Cas9 system. *Appl Environ Microbiol* **81**, 2506–2514 (2015).

6. J. Guo, *et al.*, Improved sgRNA design in bacteria via genome-wide activity profiling. *Nucleic Acids Res* **46**, 7052–7069 (2018).

7. T. Wang, *et al.*, Pooled CRISPR interference screening enables genome-scale functional genomics study in bacteria with superior performance. *Nat Commun* **9**, 2475 (2018).

8. B. M. Babin, *et al.*, Selective proteomic analysis of antibiotic-tolerant cellular subpopulations in pseudomonas aeruginosa biofilms. *mBio* **8**, 10.1128/mbio.01593-17 (2017).

9. H. M. Salis, E. A. Mirsky, C. A. Voigt, Automated design of synthetic ribosome binding sites to control protein expression. *Nat. Biotechnol.* **27**, 946–950 (2009).

10. V. Hong, S. I. Presolski, C. Ma, M. G. Finn, Analysis and optimization of copper-catalyzed azide-alkyne cycloaddition for bioconjugation. *Angew Chem Int Ed Engl* **48**, 9879–9883 (2009).

11. F. Pedregosa, *et al.*, Scikit-learn: Machine learning in python. *Journal of Machine Learning Research* **12**, 2825–2830 (2011).

12. A. Paszke, *et al.*, PyTorch: An imperative style, high-performance deep learning library in *Advances in Neural Information Processing Systems*, H. Wallach, *et al.*, Eds. (Curran Associates, Inc., 2019).

13. M. D. Zeiler, ADADELTA: An adaptive learning rate method. (2012).

14. S. M. Lundberg, S.-I. Lee, A unified approach to interpreting model predictions in *Proceedings of the 31st International Conference on Neural Information Processing Systems*, NIPS'17., (Curran Associates Inc., 2017), pp. 4768–4777.

15. D. Szklarczyk, *et al.*, The STRING database in 2023: protein-protein association networks and functional enrichment analyses for any sequenced genome of interest. *Nucleic Acids Res* **51**, D638–D646 (2023).

16. C. von Mering, *et al.*, STRING: known and predicted protein-protein associations, integrated and transferred across organisms. *Nucleic Acids Res.* **33**, D433-7 (2005).

17. J. M. Flynn, S. B. Neher, Y. I. Kim, R. T. Sauer, T. A. Baker, Proteomic discovery of cellular substrates of the ClpXP protease reveals five classes of ClpX-recognition signals. *Mol Cell* **11**, 671–683 (2003).

18. F. R. Blattner, *et al.*, The complete genome sequence of Escherichia coli K-12. *Science* **277**, 1453–1462 (1997).
